# Application of exome sequencing for prenatal diagnosis of fetal structural anomalies: clinical experience and lessons learned from a cohort of 1618 fetuses

**DOI:** 10.1186/s13073-022-01130-x

**Published:** 2022-10-28

**Authors:** Fang Fu, Ru Li, Qiuxia Yu, Dan Wang, Qiong Deng, Lushan Li, Tingying Lei, Guilan Chen, Zhiqiang Nie, Xin Yang, Jin Han, Min Pan, Li Zhen, Yongling Zhang, Xiangyi Jing, Fucheng Li, Fatao Li, Lina Zhang, Cuixing Yi, Yingsi Li, Yan Lu, Hang Zhou, Ken Cheng, Jian Li, Lina Xiang, Jing Zhang, Sha Tang, Ping Fang, Dongzhi Li, Can Liao

**Affiliations:** 1 Department of Prenatal Diagnostic Center, Guangzhou Women and Children’s Medical Center, Guangzhou Medical University, Guangzhou, 510623 Guangdong China; 2 Department of Epidemiology Division, Guangdong Cardiovascular Institute, Guangdong General Hospital, Guangzhou, 510080 Guangdong China; 3 KingMed Diagnostics, Guangzhou, Guangdong China

**Keywords:** Prenatal diagnosis, Genotype-driven, Multidisciplinary model, Exome sequencing, Structural anomaly, pES, Ultrasound

## Abstract

**Background:**

Exome sequencing (ES) is becoming more widely available in prenatal diagnosis. However, data on its clinical utility and integration into clinical management remain limited in practice. Herein, we report our experience implementing prenatal ES (pES) in a large cohort of fetuses with anomalies detected by ultrasonography using a hospital-based in-house multidisciplinary team (MDT) facilitated by a three-step genotype-driven followed by phenotype-driven analysis framework.

**Methods:**

We performed pES in 1618 fetal cases with positive ultrasound findings but negative for karyotyping and chromosome microarray analysis between January 2014 and October 2021, including both retrospective (*n*=565) and prospective (*n*=1053) cohorts. The diagnostic efficiency and its correlation to organ systems involved, phenotypic spectrum, and the clinical impacts of pES results on pregnancy outcomes were analyzed.

**Results:**

A genotype-driven followed by phenotype-driven three-step approach was carried out in all trio pES. Step 1, a genotype-driven analysis resulted in a diagnostic rate of 11.6% (187/1618). Step 2, a phenotype-driven comprehensive analysis yielded additional diagnostic findings for another 28 cases (1.7%; 28/1618). In the final step 3, data reanalyses based on new phenotypes and/or clinical requests found molecular diagnosis in 14 additional cases (0.9%; 14/1618). Altogether, 229 fetal cases (14.2%) received a molecular diagnosis, with a higher positive rate in the retrospective than the prospective cohort (17.3% vs. 12.4%, *p*<0.01). The diagnostic rates were highest in fetuses with skeletal anomalies (30.4%) and multiple organ involvements (25.9%), and lowest in fetuses with chest anomalies (0%). In addition, incidental and secondary findings with childhood-onset disorders were detected in 11 (0.7%) cases. Furthermore, we described the prenatal phenotypes for the first time for 27 gene-associated conditions (20.0%, 27/135) upon a systematic analysis of the diagnosed cases and expanded the phenotype spectrum for 26 (19.3%) genes where limited fetal phenotypic information was available. In the prospective cohort, the combined prenatal ultrasound and pES results had significantly impacted the clinical decisions (61.5%, 648/1053).

**Conclusions:**

The genotype-driven approach could identify about 81.7% positive cases (11.6% of the total cohort) with the initial limited fetal phenotype information considered. The following two steps of phenotype-driven analysis and data reanalyses helped us find the causative variants in an additional 2.6% of the entire cohort (18.3% of all positive findings). Our extensive phenotype analysis on a large number of molecularly confirmed prenatal cases had greatly enriched our current knowledge on fetal phenotype-genotype correlation, which may guide more focused prenatal ultrasound in the future. This is by far the largest pES cohort study that combines a robust trio sequence data analysis, systematic phenotype-genotype correlation, and well-established MDT in a single prenatal clinical setting. This work underlines the value of pES as an essential component in prenatal diagnosis in guiding medical management and parental decision making.

**Supplementary Information:**

The online version contains supplementary material available at 10.1186/s13073-022-01130-x.

## Background

Ultrasound detectable significant fetal abnormalities occur in 2–3% of pregnancies. Fetal congenital anomalies (CA) increase infant morbidity and mortality but also cause intangible suffering to the family [1]. Therefore, it is crucial to adopt timely and accurate diagnoses as well as appropriate interventions for congenital anomalies. The diagnosis of complicated fetal conditions is becoming increasingly sophisticated and has prompted the emergence of an entirely new clinical field. Fetal medicine differs from obstetrics, or maternal-fetal medicine, in that the fetus is the principal focus of attention. Managing the fetal patient requires the expertise of various clinicians, including fetal medicine experts, prenatal imaging practitioners, genetic counselors to neonatologists, and pediatric surgical and medical subspecialists [2].

Fetal structural anomalies are the main indication for invasive prenatal genetic testing, traditionally completed by G-banding karyotype analysis and chromosome microarray analysis (CMA). The results relate to clinical prognosis assessment, perinatal management, recurrence risk assessment, and future family planning. However, in more than half of the fetal structural anomaly cases, the molecular etiology is unknown, resulting in challenges in parental counseling. Next-generation sequencing technology has been proven to be a powerful tool for the clinical diagnosis of Mendelian disorders [3, 4]. Exome sequencing (ES) has become a first-tier clinical diagnostic test for children with neurodevelopmental disorders [5]. The most recent clinical guideline of the American College of Medical Genetics and Genomics (ACMG) recommended exome and genome sequencing as a first-tier or second-tier test for patients with one or more CAs prior to 1 year of age or for patients with developmental delay or intellectual disability with onset prior to 18 years of age [6]. Given the success in postnatal patient populations and the limitations of current genetic testing for prenatal cases, ES is now applied to prenatal diagnosis (pES) more widely. There have been several reports on the application of ES in prenatal diagnosis in relatively large sample sizes (*n*>100) [7–13], with diagnostic rates ranging from 8.5 to 35% [14]. However, data on integrating ES into clinical practice and returning results during pregnancy remain limited.

We report our 8-year clinical experience from a single clinical hospital applying pES in a cohort of 1618 fetuses with structural anomalies. ES data analyses were carried out in a three-step approach. In brief, the first genotype-driven step prioritized the variants based on pathogenicity and allele origin/zygosity without phenotypic data, the second phenotype-driven comprehensive analysis step was performed based on initial indications, and the third data reanalyses step were implemented in cases with new phenotypic information and/or upon the physicians’ request. In particular, we evaluate the diagnostic efficiency of pES, delineate the disease spectrum of the study cohort, and assess the clinical impacts of prenatal ES on medical management changes, including delivery plan modifications and neonatal management.

## Methods

### Study cohort

This study was approved by the Institutional Review Board of the Ethics Committee in the Guangzhou Women and Children’s Medical Center, and written informed consent was obtained from the expecting couples for invasive prenatal diagnosis. The study cohort was recruited between January 2014 and October 2021. The involved patients came from the Multidisciplinary Clinic of Fetal Medicine at Guangzhou Women’s and Children’s Medical Center, the largest specialist hospital serving patients in Southern China and throughout the country. The Fetal Medicine Multidisciplinary Clinic was established in 2010, and the multidiscipline team met with couples with abnormal fetuses regularly, providing couples with valuable information on the diagnosis or treatment of fetal abnormalities, as well as subsequent clinical pathways and technical team support.

Fetuses in accordance with the following criteria were included: (1) fetuses were diagnosed with increased nuchal translucency (NT >3.5mm), fetal hydrops, and other structural anomalies by prenatal imaging; (2) samples for both parents were available; (3) the quantity/quality of fetal DNA sample was sufficient for ES test. All fetuses underwent karyotype and/or CMA (Cytocan 750K/HD, Affymetrix) testing before pES, and those with aneuploidies, chromosome rearrangement, and clinically significant copy number variations detected were excluded from this cohort. In total, ES was performed successfully in 1618 fetuses (chorionic villi *n*=139, amniocytes *n*=971, and cord blood *n*=508) and their parents (and other informative 1st or 2nd degree relatives including siblings, uncles, aunts, or grandparents, *n*=126). According to the time point of the ES test, 1618 cases were divided into the retrospective cohort (WES was performed at the end of a pregnancy, with no impacts on pregnancy decisions and as scientific research subsidized by research funding with no cost to the patients’ families, between January 2014 and July 2017, *n*= 565) and the prospective cohort (WES was performed during an ongoing pregnancy, as routine fee-for-service in clinical settings, between August 2017 and October 2021, *n*= 1053).

### Exome sequencing, analysis, and interpretation

**Fig. 1:**
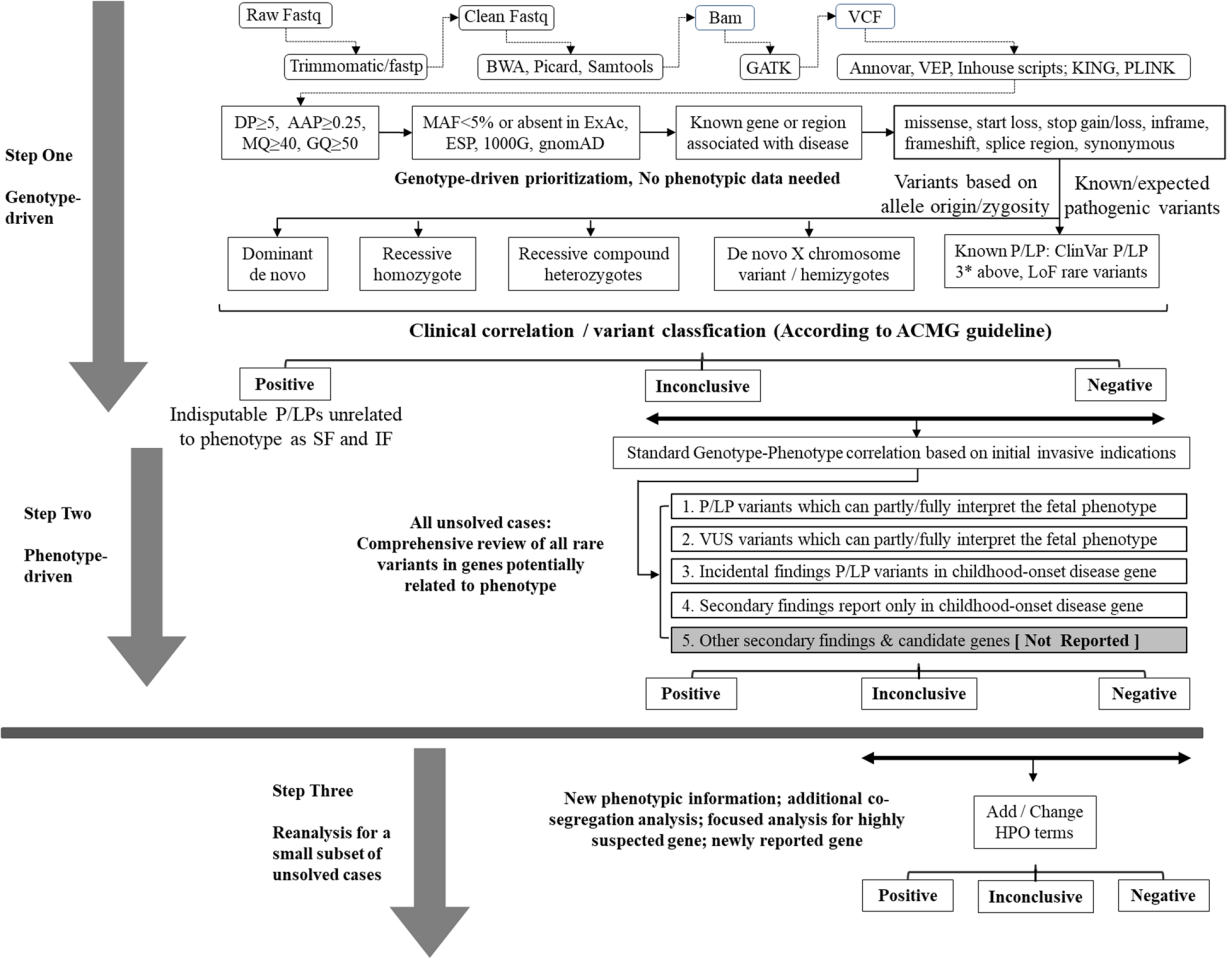
The pipeline of prenatal ES data analysis in our cohort study

Fetal samples were collected from chorionic villi, amniocytes, or cord blood depending on gestational age, and samples from parents and other relatives were obtained from peripheral blood. All genomic DNA was extracted using a Qiagen DNA Blood Mini kit (Qiagen, Germany) following the manufacturer’s protocol. Agilent or Integrated DNA Technologies kits were used for target enrichments, followed by 150-base pair reads sequenced using Illumina HiSeq2500, HiSeq Xten, or NovaSeq platforms. An overview of pES data analysis and interpretation logistics is summarized in Fig. 1. Detailed information for methods in this process is provided in Additional file 1. In brief, raw fastq data were analyzed with an in-house pipeline and local reference samples (more than 10,000 individuals, including patients and healthy individuals), briefly including mapping, realignment, variant calling, quality control, variant filtration, annotation, sex, and family pedigree relationship confirmation. All the single-nucleotide variants (SNVs) and indels detected were classified into five levels (pathogenic/P, likely pathogenic/LP, uncertain/VUS, likely benign/LB, and benign/B) based on the ACMG and ClinGen variant curation expert panel guidelines [15–17].

ES data analyses were carried out in a stepwise model (Fig. 1). The first genotype-driven step prioritized the variants based on pathogenicity and allele origin/zygosity without phenotypic data. Variants captured in this step included known/expected disease-causing alleles (reported indisputable P/LPs and extremely rare null changes) and/or rare variants most likely to be causative based on inheritance pattern (for example, de novo, homozygous, compound heterozygous, and hemizygous variants). The resultant short gene/variant list for each trio was reviewed for brief clinical correlation, in silico prediction, minor allele frequency, relevant literature, and annotation in ClinVar and Human Gene Mutation Database (HGMD) databases, and the ACMG and ClinGen expert panel recommendations were utilized in variant classification. In the second phenotype-driven analysis step, a comprehensive review of rare variants in genes potentially related to initial clinical indications for prenatal diagnosis was performed. In the third analysis step, data reanalyses were implemented in cases with new phenotypic information acquired during the prenatal and/or postnatal period and/or upon the physicians’ request. The standard Human Phenotype Ontology (HPO) terms were matched to the clinical synopsis of the P/LP/VUS disease genes, and ES results were finally classified into five tiers: (1) Positive diagnostic result: P/LP variants identified in a disease gene that can explain (partially or fully) the fetal phenotype; (2) Inconclusive: variant of unknown significance (VUS) identified in a disease gene which can explain (partially or fully) the fetal phenotype; (3) Incidental findings (IFs): P/LP variants identified in childhood-onset disease gene, unrelated to fetal phenotype; (4) Secondary findings (SFs): P/LP variants identified in ACMG recommended SF2.0 gene list, unrelated to fetal phenotype [18, 19]; (5) Candidate genes: variants (mostly de novo) predicted to be deleterious and absent in general population, identified in undefined disease genes that have a paralog gene or previously published data to support the association with fetal anomalies, or based on animal model and tissue expression [20, 21]. All diagnostic de novo variants were validated by Sanger sequencing.

The ES report included positive and inconclusive results related to primary prenatal indications. Incidental and secondary findings with a childhood-onset disease were also included in the report, based on consensus between laboratory and clinicians. Secondary findings for a late-onset disease were not routinely reported. For the retrospective cohort, the results were reported to the couples as scientific research data postnatally. For the prospective cohort, the results were reported once the test was concluded.

The pregnancy outcome and clinical follow-up data were collected until December 2021. Clinical impacts were assessed both in retrospective and prospective cohorts. Statistical analyses were performed using Fisher’s exact test, Pearson’s correlation test, or analysis of variance (ANOVA).

The resources and datasets used in this study included 1000 Genome Project (https://www.internationalgenome.org/), Genome Aggregation Database ( https://gnomad.broadinstitute.org/), Exome Aggregation Consortium (https://exac.broadinstitute.org/), Exome Sequencing Project (https://evs.gs.washington.edu/EVS/), ClinVar (https://www.ncbi.nlm.nih.gov/clinvar/), ClinGen (https://clinicalgenome.org/), Human Gene Mutation Database (https://www.hgmd.cf.ac.uk/ac/index.php), Online Mendelian Inheritance in Man (https://omim.org/), Orphanet (https://www.orpha.net/consor/cgi-bin/index.php), UniProtKB (https://www.uniprot.org/help/uniprotkb), Human Protein Reference Database (http://www.hprd.org/), Geneimprint (https://www.geneimprint.com/), MetaImprint (http://bioinfo.hrbmu.edu.cn/MetaImprint), UCSC Genome Browser (http://genome.ucsc.edu/), Human Phenotype Ontology (https://hpo.jax.org/). The detailed information for tools and software used is provided in Additional file 1.

## Results

### Cohort characteristics

Trio exome sequencing was performed in 1618 fetuses. At testing time, the median maternal age was 29 years (range 18–47), and the median gestational age was 25 weeks (range 11–35). Based on the systems involved, fetal malformations were classified into 8 categories (central nervous, facial, chest, cardiovascular, abdominal, urogenital, skeletal, and multisystem). Isolated hydrops fetalis, fetal growth retardation (FGR), and increased NT were classified into separate categories. For fetuses with increased NT, if a new phenotype appeared in late pregnancy, they would be reclassified into the corresponding malformation categories. The most frequently affected organ referred for prenatal ES was the cardiovascular system (17.9%), followed by the central nervous (16.2%), skeletal (12.8%), and urogenital (11.4%) systems, altogether comprising more than half of all cases. The detailed clinical characteristics of the fetal cases are summarized in Additional file 2: Table S1. The turn-around time of prenatal ES was 4–10 weeks in the retrospective cohort and 1–4 weeks in the prospective cohort.

### Positive diagnostic results and data reanalysis

In genotype-centric analysis step 1, P/LP variants were identified in 187 cases, representing an 11.6% diagnostic rate (187/1618). VUSes were identified in 55 cases (3.4%, 55/1618). Step 2 shows phenotype-driven analysis resulted in additional positive diagnostic variants in 28 cases (1.7%, 28/1618). In addition, VUSes potentially related to phenotypes were identified in 68 cases (4.2%, 68/1618).

In step 3 for data reanalysis, 295 cases obtained additional new phenotypes during the prenatal or perinatal period. One case received upgrades from inconclusive to positive based on further phenotypic information. After reanalysis, P/LP variants previously interpreted as incidental findings in steps 1 and 2 in 7 cases (0.4%, 7/1618) were reclassified as disease-related based on additional phenotypes in late pregnancy. The detailed information of fetuses with diagnostic or VUS results with new phenotypes is summarized in Additional file 2: Table S2. In addition, 3 cases (0.2%, 3/1618) were upgraded from negative to P/LP due to a new disease gene identified in reanalysis upon special clinical requests, 2 cases with intragenic copy number variants, and 1 case with a second allele identified by Sanger sequencing were revealed by reanalysis. Altogether, data reanalysis yielded an increased diagnostic rate of 0.9% (14/1618), of which 50.0% (7/14) attributed to new phenotypes reclassified from IFs to positive diagnoses, and 21.4% (3/14) attributed to new disease genes identified. In addition, eight cases were upgraded from negative to inconclusive based on the newly provided phenotype, yielding a VUS rate of 0.5% (8/1618). The overall positive diagnostic and VUS rates of each analysis step are summarized in Table 1.

**Table 1:** The overall positive diagnostic and inconclusive rates in each analysis step

Analysis step	Diagnostic results	Inconclusive results
**Step 1** (Genotype-driven)	**187/1618 (11.6%)**	**55/1618 (3.4%)**
**Step 2** (Genotype-Phenotype correlation based on initial invasive indications)	**28/1618 (1.7%)**	**68/1618 (4.2%)**
	19 due to genotype-phenotype correlations	
	9 due to family co-segregation	
**Step 3** (Reanalysis due to new phenotypes or physician’s requests)	**14/1618 (0.9%)**	**8/1618 (0.5%)**
	1 due to new phenotypes upgrading from VUS to LP	
	7 due to new phenotypes reclassified from IFs to positive diagnoses	
	3 due to new disease genes identified	
	2 with intragenic copy number variants	
	1 with focused Sanger analysis for a disease gene possessing pseudogene sequence	
**Total**	**229/1618 (14.2%)**	**131/1618 (8.1%)**

In total, 253 different variants across 135 unique genes were identified as positive diagnoses in 229 fetal cases (Additional file 2: Table S3), with an overall diagnostic rate of 14.2% (229/1618). Of these, 98 diagnostic variants (38.7%, 98/253) were not previously reported. Of the 135 genes identified, 26 (19.3%) were revealed to expand the previously reported fetal phenotype spectrum; 11 were outside the list in the PAGE study [8]. Twenty-seven genes (20.0%, 27/135) were reported in prenatal cases for the first time, of which potential fetal phenotype expansion was identified in 13 genes (Table 2). Among the positive diagnostic cases, 172 (75.1%), 40 (17.5%), and 17 (7.4%) were associated with autosomal dominant, recessive, and X-linked disorders, respectively (Table 3). In our cohort, 134 cases had a family history record with prior affected pregnancies or relatives, including 100 with similar phenotypes (significant family history) and 34 with different phenotypes. The diagnostic rate was 48.0% (48/100) in cases with a significant family history, significantly higher than that for sporadic cases (11.8%, *p*<0.01).

**Table 2:** The 27 genes firstly reported in prenatal cases

Case ID	Gender	Ultrasound findings	Gene(OMIM ID)	Transcript	Nucleotide change	Amino acid change	Variant type	Zygosity	Classification	ACMG codes	Origin	Inheritance	Disease (OMIM ID)	Evidence for causality	Possible expansion of fetal phenotypes
**4**	Female	Agenesis of corpus callosum, ventriculomegaly	NFIA(600727)	NM_001145512.1	c.1112C>A^b^	p.(Ser371Ter)	Nonsense	Het	P	PVS1, PS2, PM2	De novo	AD	BRAIN MALFORMATIONS WITH OR WITHOUT URINARY TRACT DEFECTS(613735)	Matching HPO entry: Agenesis of corpus callosum, Ventriculomegaly	─
**19**	Male	Increased nuchal translucency, agenesis of corpus callosum, ventriculomegaly	NFIA(600727)	NM_001145512.1	c.483A>C^b^	p.(Arg161Ser)	Missense	Het	LP	PS2, PM1, PM2, PP3	De novo	AD	BRAIN MALFORMATIONS WITH OR WITHOUT URINARY TRACT DEFECTS(613735)	Matching HPO entry: Agenesis of corpus callosum, Ventriculomegaly	Increased nuchal translucency
**7**	Male	Widened posterior fossa	EZH2(601573)	NM_004456.4	c.2050C>T	p.(Arg684Cys)	Missense	Het	P	PS2, PS3_PM, PS4_PP, PM2, PM5, PP3	De novo	AD	WEAVER SYNDROME(277590)	Matching central nervous system	Widened posterior fossa
**108**	Male	Hypoplasia of the ulna, radial dysplasia, abnormality of digit	EZH2(601573)	NM_004456.4	c.47G>A^b^	p.(Arg16Gln)	Missense	Het	LP	PS2_PM, PM1_PP, PM2, PP3	De novo	AD	WEAVER SYNDROME(277590)	Matching HPO entry: Abnormality of digit	Hypoplasia of the ulna, Radial dysplasia
**12**	Female	Hypoplasia of the corpus callosum, ventriculomegaly	PPP2R1A(605983)	NM_014225.5	c.775G>A	p.(Val259Ile)	Missense	Het	LP	PS2, PM2, PP3	De novo	AD	MENTAL RETARDATION, AUTOSOMAL DOMINANT 36; MRD36(616362)	Matching HPO entry: Hypoplasia of the corpus callosum, Ventriculomegaly	─
**30**	Male	Hydrocephalus, hypoplasia of the corpus callosum	PPP2R1A(605983)	NM_014225.5	c.544C>T	p.(Arg182Trp)	Missense	Het	P	PM1, PM2, PS3_PP, PS2_PVS	De novo	AD	MENTAL RETARDATION, AUTOSOMAL DOMINANT 36 (616362)	Matching HPO entry: Hydrocephalus, Hypoplasia of the corpus callosum	─
**21**	Male	Increased nuchal translucency, ventriculomegaly	ADNP(611386)	NM_015339.4	c.2161C>T^b^	p.(Gln721Ter)	Nonsense	Het	LP	PVS1_PS, PS2_PM, PM2	De novo	AD	HELSMOORTEL-VAN DER AA SYNDROME(615873)	Matching HPO entry: Ventriculomegaly	Increased nuchal translucency
**46**	Male	Tetralogy of Fallot	ADNP(611386)	NM_015339.4	c.2156dupA	p.(Tyr719Ter)	Frameshift	Het	P	PVS1_PS, PS2, PM2	De novo	AD	HELSMOORTEL-VAN DER AA SYNDROME(615873)	Matching cardiovascular system	─
**18**	Female	Dilation of lateral ventricles, polyhydramnios	ARV1(611647) ^a^	NM_022786.3	c.409delG^b^	p.(Glu137AsnfsTer13)	Frameshift	Het	P	PVS1, PM2, PM3	Pat	AR	EPILEPTIC ENCEPHALOPATHY, EARLY INFANTILE, 38 (617020)	Matching central nervous system	Dilation of lateral ventricles, Polyhydramnios
				NM_022786.3	c.518dupA	p.(Pro174AlafsTer14)	Frameshift	Het	P	PVS1, PM2_PP, PM3	Mat				
**31**	Male	Bilateral choroid plexus cyst, single umbilical artery	ZMYM2(602221) ^a^	NM_003453.4	c.534dupA^b^	p.(Asp179ArgfsTer3)	Frameshift	Het	P	PVS1, PS2_PP, PM2	De novo	AD	Neurodevelopmental craniofacial syndrome with variable renal and cardiac abnormalities(619522)	Matching central nervous system	Bilateral choroid plexus cyst, Single umbilical artery
**38**	Male	Micrognathia, cleft palate	KCNK9(605874)	NM_001282534.1	c.706G>C	p.(Gly236Arg)	Missense	Het	P	PS2_PVS, PS3_PP, PM2	De novo	AD	BIRK-BAREL SYNDROME(612292)	Matching HPO entry: Micrognathia, Cleft palate	─
**40**	Male	Congenital cataract	CRYAA(123580)	NM_000394.3	c.34C>T	p.(Arg12Cys)	Missense	Het	P	PS4, PM2, PP1_PS, PP3	Pat(affected)	AD	CATARACT 9, MULTIPLE TYPES; CTRCT9(604219)	Matching HPO entry: Congenital cataract	─
**65**	Female	Transposition of the great arteries	MAPK1(176948) ^a^	NM_002745.4	c.1061T>G	p.(Phe354Cys)	Missense	Het	LP	PS2, PM2, PP3, PP2	De novo	AD	Noonan syndrome 13 (619087)	Matching cardiovascular system	─
**102**	Male	Short long bone, wind-swept deformity of the knees	GDF5(601146)	NM_000557.5	c.1335T>G	p.(Asn445Lys)	Missense	Het	P	PS2_PM, PM1_PP, PM2, PP3	De novo	AD	MULTIPLE SYNOSTOSES SYNDROME 2 (610017)	Matching HPO entry: Short long bone	─
**107**	Male	Redundant neck skin, abnormal posturing, flexion contracture	SCN4A(603967)	NM_000334.4	c.3502delC^b^	p.(Leu1168SerfsTer5)	Frameshift	Het	LP	PVS1, PM2	Mat	AR	MYASTHENIC SYNDROME, CONGENITAL, 16 (614198)	Matching HPO entry: Flexion contracture	─
				NM_000334.4	c.3395G>C^b^	p.(Arg1132Pro)	Missense	Het	LP	PM2, PM3_PP, PM5, PP3	De novo				
**110**	Female	Abnormality of the middle and the distal phalanx of the 3rd finger, abnormality of the index finger, split foot	PUF60(604819)	NM_078480.2	c.24+3A>T^b^	-	Splice region	Het	LP	PVS1_PM, PS2_PM, PM2	De novo	AD	VERHEIJ SYNDROME(615583)	Matching skeletal system	Split foot
**114**	Female	Talipes equinovarus, abnormality of the hand	ZC4H2(300897)	NM_018684.3	c.562-1G>T^b^	-	Splice acceptor	Het	P	PVS1_PS, PS2	De novo	XL	WIEACKER-WOLFF SYNDROME, FEMALE-RESTRICTED(301041)	Matching HPO entry: Talipes equinovarus, Abnormality of the hand	─
**116**	Female	Short long bone	SMAD4(600993)	NM_005359.5	c.1498A>G	p.(Ile500Val)	Missense	Het	P	PS2, PS4, PM2_PP, PM5, PP3	De novo	AD	MYHRE SYNDROME(139210)	Matching HPO entry: Short long bone	─
**118**	Female	Talipes equinovarus, hand clenching	TPM2(190990)	NM_003289.4	c.463G>A	p.(Ala155Thr)	Missense	Het	LP	PS3_PP, PS4_PM, PM2, PP3	Mat (affected)	AD	ARTHROGRYPOSIS, DISTAL, TYPE 1A(108120)	Matching HPO entry: Talipes equinovarus, Hand clenching	─
**159**	Female	Talipes equinovarus	BRPF1(602410)	NM_001003694.1	c.1723-1G>C^b^	-	Splice acceptor	Het	P	PS2, PM2, PVS1_PS	De novo	AD	INTELLECTUAL DEVELOPMENTAL DISORDER WITH DYSMORPHIC FACIES AND PTOSIS (617333)	Matching HPO entry: Talipes equinovarus	─
**163**	Male	Intrauterine growth retardation	KAT6A(601408)	NM_006766.4	c.751C>T^b^	p.(Arg251Ter)	Nonsense	Het	P	PVS1, PS2, PM2	De novo	AD	ARBOLEDA-THAM SYNDROME(616268)	Matching HPO entry: Intrauterine growth retardation	─
**169**	Male	Increased nuchal translucency	KMT2C(606833)	NM_170606.2	c.12906delT^b^	p.(Ala4303ProfsTer23)	Frameshift	Het	P	PVS1, PS2, PM2	De novo	AD	KLEEFSTRA SYNDROME 2 (261652)	Not reported previously. Homologous genes KMT2A/KMT2D matching HPO entry: Increased nuchal translucency	Increased nuchal translucency
**170**	Male	Increased nuchal translucency, bilateral choroid plexus cysts	KCNT1(608167)	NM_020822.2	c.1420C>T	p.(Arg474Cys)	Missense	Het	P	PM2, PP3, PS2_PVS	De novo	AD	DEVELOPMENTAL AND EPILEPTIC ENCEPHALOPATHY 14 (614959)	Not reported previously	Increased nuchal translucency, Bilateral choroid plexus cysts
**172**	Female	Increased nuchal translucency, cystic hygroma	NFIB(600728)	NM_001190737.1	c.376A>G	p.(Lys126Glu)	Missense	Het	LP	PM1, PP3, PS3_PP, PS2_PP, PM2	De novo	AD	MACROCEPHALY, ACQUIRED, WITH IMPAIRED INTELLECTUAL DEVELOPMENT(618286)	Not reported previously	Increased nuchal translucency, cystic hygroma
**189**	Male	Micrognathia, arthrogryposis multiplex congenita, FGR	UBA1(314370)	NM_153280.2	c.1617G>T	p.(Met539Ile)	Missense	Hemi	LP	PM1, PM2, PP3, PP4	Mat	XR	SPINAL MUSCULAR ATROPHY, X-LINKED 2 (301830)	Matching HPO entry: Micrognathia, Arthrogryposis multiplex congenita	─
**206**	Female	Increased nuchal translucency, abnormality of the hand, intracranial hemorrhage, widened posterior fossa, porencephalic cyst	PLOD3(603066)	NM_001084.4	c.1890T>G	p.(Tyr630Ter)	Nonsense	Het	LP	PVS1,PM2	Mat	AR	BONE FRAGILITY WITH CONTRACTURES, ARTERIAL RUPTURE, AND DEAFNESS(612394)	Matching HPO entry: Abnormality of the hand, Intracranial hemorrhage	Increased nuchal translucency, Widened posterior fossa, Porencephalic cyst
				NM_001084.4	c.1354C>T	p.(Arg452Ter)	Nonsense	Het	LP	PVS1,PM2	Pat				
**218**	Female	Ventriculomegaly, Tetralogy of Fallot, right aortic arch	MTOR(601231)	NM_004958.3	c.7255G>A	p.(Glu2419Lys)	Missense	Het	P	PS2, PM2, PP3, PP2	De novo	AD	SMITH-KINGSMORE SYNDROME(616638)	Matching HPO entry: Ventriculomegaly	Tetralogy of Fallot, Right aortic arch
**219**	Male	Polyhydramnios, short long bone, cardiomegaly	KMT2A(159555)	NM_001197104.1	c.4219-1G>A^b^	-	Splice acceptor	Het	P	PM2, PVS1, PS2_PM	De novo	AD	WIEDEMANN-STEINER SYNDROME(605130)	Matching HPO entry: Short long bone	Polyhydramnios, Cardiomegaly
**221**	Male	Aplasia of the nasal bone, abnormality of ductus venosus blood flow, single umbilical artery, ventriculomegaly, Dandy-Walker malformation, congenital diaphragmatic hernia	ARID1A(603024)	NM_006015.4	c.5853dupC^b^	p.(Ile1952HisfsTer11)	Frameshift	Het	P	PS2, PM2, PVS1_PS	De novo	AD	COFFIN-SIRIS SYNDROME 2 (614607)	Matching HPO entry: Ventriculomegaly, Dandy-Walker malformation	─
**225**	Male	Supraventricular tachycardia, enlarged cisterna magna, ascites, pleural effusion, polyhydramnios	CSNK2A1(115440)	NM_001895.3	c.838C>T^b^	p.(Arg280Ter)	Nonsense	Het	LP	PVS1_PS, PM1, PM2	De novo	AD	OKUR-CHUNG NEURODEVELOPMENTAL SYNDROME(617062)	Matching cardiovascular system; PMID: 29568000	Supraventricular tachycardia, Pleural effusion, Polyhydramnios
**229**	Male	Large for gestational age, polyhydramnios	CLCN5(300008) ^a^	NM_001127898.3	c.934-1G>T^b^	-	Splice acceptor	Hemi	LP	PVS1_PS, PM1, PM2	Mat	XR	DENT DISEASE 1 (300009)	PMID: 18540256, 27174143	─

^a^Genes outside the list in PAGE study (DDG2P 1856 genes downloaded in July 2019 plus 117 prenatal associated genes)

^b^Novel variants identified in this study

**Table 3:** Inheritance patterns of positive diagnostic and VUS cases

Inheritance patterns	Total	AD		AR	XL	
		De novo	Inherited from the parent		De novo	Maternally inherited
**Positive diagnostic cases**	**229**	**145**	**27**	**40**	**7**	**10**
Retrospective cohort	98	70	5	12	5	6
Significant history/family history	11/12	0/1	5/5	4/4	0/0	2/2
Prospective cohort	131	75	22	28	2	4
Significant history/family history	37/42	1/5	15/15	17/18	0/0	4/4
**VUS cases**	**131**	**36**	**17**	**60**	**3**	**15**
Retrospective cohort	48	26	0	16	2	4
Significant history/family history	0/1	0/0	0/0	0/0	0/0	0/1
Prospective cohort	83	10	17	44	1	11
Significant history/family history	13/14	0/0	4/5	8/8	0/0	1/1
**Negative cases**	**1246**					
Retrospective cohort	411					
Significant history/family history	12/13					
Prospective cohort	835					
Significant history/family history	27/52					

**Fig. 2:**
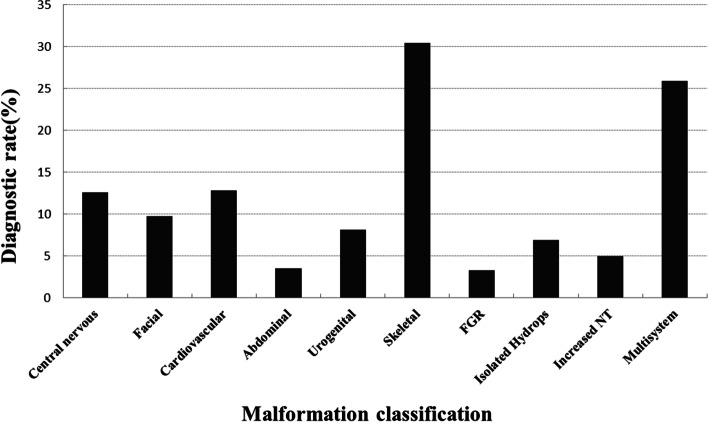
Diagnostic rates based on malformation classification. The highest diagnostic rates were obtained in fetuses with multiple organ and skeletal anomalies and the lowest in fetuses with chest anomalies

The diagnostic rate in the retrospective cohort was significantly higher than that in the prospective cohort (17.3% vs. 12.4%, *p*<0.01). Significantly higher diagnostic rates were obtained in fetuses with abnormalities in skeletal systems (30.4%) and multiple organ systems (25.9%) than in other subgroups (*p*< 0.05) (Fig. 2, Additional file 2: Table S4). No molecular diagnosis was made by pES for the 46 fetuses with chest malformations.

Among the diagnosed cases, genes associated with neurodevelopmental disorders were found in all patients with central nervous anomalies (*n*=33). Positive cases with multisystem or cardiovascular abnormalities showed significantly higher probability (85.7% and 86.5%) of harboring neurodevelopmental disorder-related variants than cases with skeletal (44.4%), facial (25.0%), and urogenital (6.7%) anomalies (*p*<0.05).

### Genotype-phenotype correlation analysis

In this study, clinical features were converted into standard HPO terms for all cases to facilitate the genotype-phenotype correlation analysis. A disease gene was considered potentially relevant to the fetal anomalies if its associated clinical phenotypes meet one of the following criteria: (1) match HPO entry of the fetal phenotype; (2) match the superclass based on HPO or clinical synopsis in Online Mendelian Inheritance in Man (OMIM) database; (3) be reported in previous cases manifesting the same or similar phenotypes of the fetuses.

Among the 229 diagnostic cases, 195 disease genes (85.2%, 195/229) matched at least one HPO term, and 27 (11.8%, 27/229) fit the superclass based on HPO or clinical synopsis in OMIM, showing atypical phenotypes. Variants in 4 genes (1.7%, 4/229) were initially considered as incidental findings and reclassified as diagnostic results due to similar phenotypes reported in the literature (cases 29, 39, 208, and 229). Three genes (*KMT2C, KCNT1, NFIB*) identified in fetuses with increased NT have not been reported prenatally (cases 169, 170, and 172).

In the 229 diagnosed cases, 49 had additional new phenotypes during prenatal and/or postnatal periods. P/LP variants identified in 7 fetuses (3.1%, 7/229) were considered as incidental findings based on initial fetal anomalies and reclassified as diagnostic variants due to new phenotypes in data reanalysis (Additional file 2: Table S2). One case (0.4%, 1/229) was upgraded from VUS to LP due to new phenotypes (case 207).

### Frequent molecular diagnosis of disease genes

**Fig. 3:**
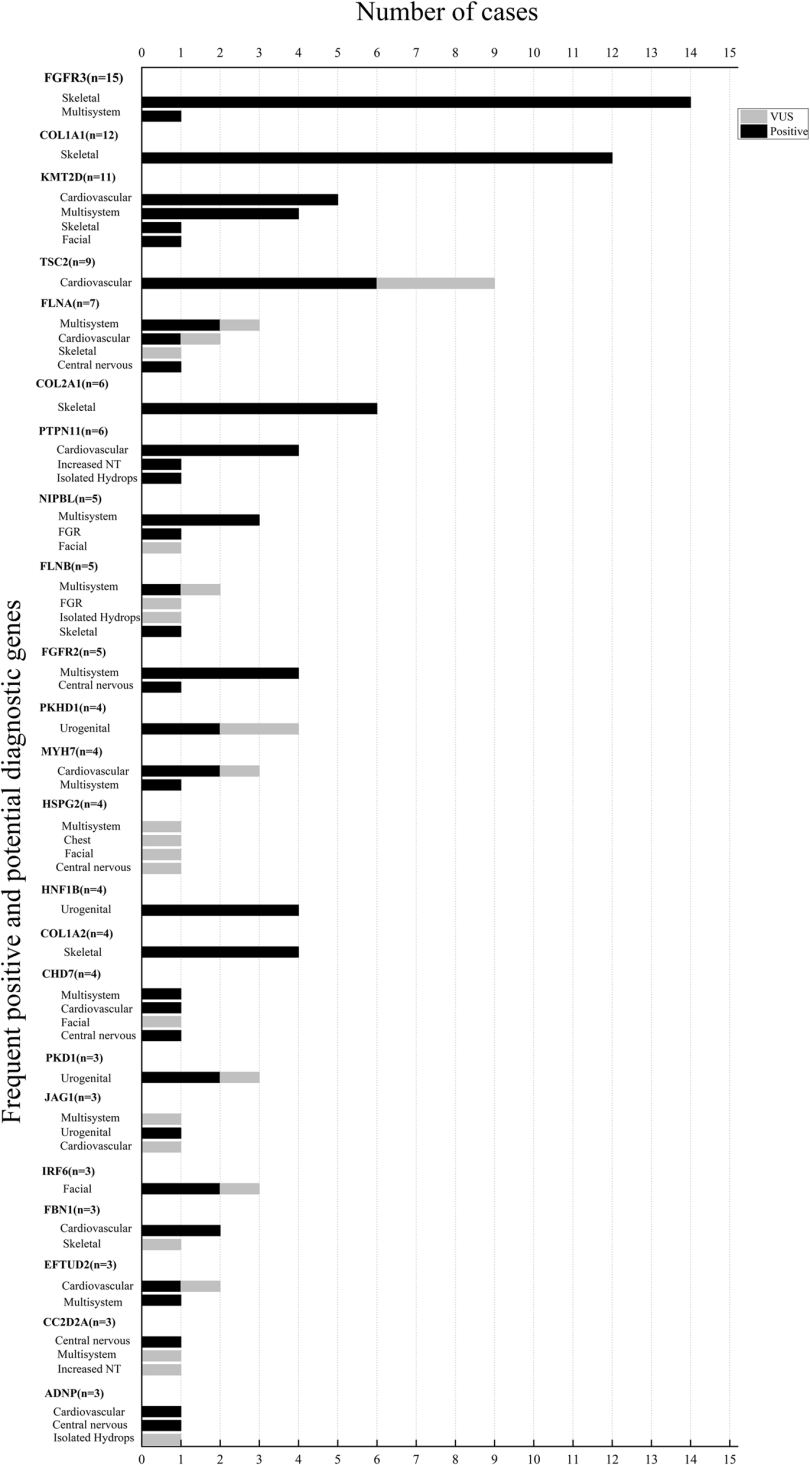
Frequent positive and potential diagnostic genes. The number of cases by disease genes in different malformation categories is shown

The most frequent diagnostic genes in 4 or more cases were *FRFR3* (*n*=15), *COL1A1* (*n*=12), *KMT2D* (*n*=11), *COL2A1* (*n*=6), *PTPN11* (*n*=6), *TSC2* (*n*=6), *FGFR2* (*n*=5), *FLNA* (*n*=4), *NIPBL* (*n*=4), *HNF1B* (*n*=4), and *COL1A2* (*n*=4) (Fig. 3).

### Intragenic copy number variants (CNVs)

While gross copy number abnormalities were ruled out by karyotype/CMA for this cohort and CNV analysis was not part of the routine pES testing, focused CNV analysis on highly suspected genes based on phenotypes was performed for selected cases upon clinicians’ requests in the reanalysis step. The potential significant CNVs were confirmed by orthologous methods for a definitive diagnosis. Such assessments resulted in 2 additional positive cases involving intragenic deletions in the *ATRX* and *SHOX* genes (0.3%; case 126 and case 162).

### Diagnostic rates in relation to NT measurement

In our cohort, NT measurement results were available for 690 cases, including 121 patients with isolated increased NT (≥3.5 mm) without concomitant anomalies (the isolated group) and 569 cases with increased NT (47 with NT ≥3.5 mm, and 522 with NT between 3.0 and 3.4 mm) associated with other structural abnormalities (the associated group; Additional file 2: Table S5). The pES detection rates were 5.0% in the isolated group and 25.5% in the associated group with NT ≥3.5 mm, respectively (*p*<0.05). For cases with isolated increased NT between 3.5 and 4.9 mm, the total diagnostic rate was 3.8% (3/78). The diagnostic rates increased with increasing NT measurement, although the correlation was not statistically significant (*p*=0.08). No such correlation was observed in the associated group.

### Number of candidate variants analyzed

The numbers of potential diagnostic/candidate variants in step 1 (genotype-driven) and step 2 (phenotype-driven) in 525 representative cases (including all or at least 50 cases from each malformation class) are analyzed and listed in Additional file 2: Table S6. The numbers of candidate variants based on malformation class and overall result category (positive, inconclusive, and negative), are summarized in Additional file 2 Table S7. The average total number of variants closely reviewed per case was 1.7, and the mean number of variants interpreted as irrelevant after a quick review was 34.3. Overall, the negative cases had a significantly lower number of variants analyzed for all 6 statistical indexes, with either candidate variants ruled out after close review or no candidates for review (*p*<0.01). Moreover, cases with multisystem malformations had the highest number of variants closely reviewed (mean 3.1) and the highest number of variants quickly ruled out (mean 55.3).

The amount of time for reviewing these candidate variant(s) for each case was approximately 15 min on average, ranging from 5 to 30 min for the vast majority of cases.

### Inconclusive results

Variants of uncertain significance were detected in 131 fetuses (Additional file 2: Table S8), including 2 cases combined with positive diagnostic variants and 15 with significant family history. ES reanalysis resulted in upgrades from negative to inconclusive for 8 cases due to new phenotypes. Therefore, the inconclusive rate in this study was 8.1% (131/1618). Twenty-four inconclusive cases (18.3%, 24/131) with fetal phenotype consistency and variants predicted to be deleterious or reported in previous cases were considered as high-risk inconclusive, including 5 cases (cases 244, 267, 347, 346, and 357) with prior similarly affected fetuses in each family.

### Incidental and secondary findings

Incidental findings with childhood-onset disease gene were revealed in 8 fetuses (0.5%, 8/1618) and secondary findings were detected in 13 fetuses (0.8%, 13/1618) according to ACMG recommended list (Additional file 2: Table S9). All the incidental findings were reported based on consensus between the laboratory and clinicians. Of note, secondary findings with childhood-onset diseases in 3 fetuses (3/13, 23.1%) were also included in the reports.

### Candidate genes

Candidate gene analyses focused on de novo etiology and prioritized 33 variants based on the combined considerations of gene function and variant type (1 nonsense, 4 frameshift, and 28 missense changes) in 31 cases (Additional file 2: Table S10). In addition, compound heterozygous missense variants in the *ASXL3* gene were identified in a family having 3 children with congenital heart defects [22]. Of these 32 cases, 16 had cardiovascular anomalies, 1 had fetal hydrops, 5 had urogenital anomalies, 4 had skeletal abnormalities, and 2 had central nervous or chest or multisystem anomalies, respectively.

### Pregnancy outcomes and assessment of the clinical impact

Pregnancy outcomes were available in 1462 (90.4%) of the 1618 cases. Ninety-five cases were lost to follow-up (5.9%, 95/1618), and 61 were still in pregnancy until February 2022. For the remaining cases, 579 were terminated, 5 were fetal demise, and 878 were live birth, including 19 with neonatal death (Additional file 2: Table S11).

In the retrospective cohort, 98 cases (17.3%, 98/565) obtained molecular diagnosis contributing to recurrence risk assessment and reproductive planning. For the 5 cases with incidental or secondary findings with childhood-onset disease genes in the retrospective cohort, 2 were terminated due to fetal anomalies, and 3 (0.5%, 3/565) were live birth with the ES results implicated for future clinical surveillance and medical management.

In the prospective cohort, clinical impacts were evaluated in all cases (Table 4). For terminated cases with diagnostic findings, 25.7% (27/105) were terminated due to fetal anomalies prior to ES results, and 74.3% (78/105) made the decision based on positive ES results. For diagnostic cases with live birth, the clinical decision of continuation of pregnancy was made in 35.0% of cases (7/20) due to non-neurodevelopmental consequences, 20.0% (4/20) due to inherited from either parent, and 40.0% (8/20) due to both effects, respectively. Overall, positive ES results contributed to clinical decision making on termination (59.5%) or continuation of pregnancy (14.5%) in 97 cases (74.0%, 97/131). Inconclusive results had a predominantly clinical impact in one case (1.2%, 1/81), manifesting increased NT and pleural effusion with decision making on termination (reported in the previous case with intellectual disability and behavior disorder [23]), and 10 cases (12.3%, 10/81) with decision making on the continuation of pregnancy due to inherited from either parent. Incidental findings had a clinical impact in 2 cases (40%, 2/5) with decision making on the continuation of pregnancy due to non-neurodevelopmental consequences and implications for clinical management. Secondary finding with a childhood-onset disease was reported in 1 case, contributing to clinical surveillance and management with live birth. For negative cases, the clinical decision to continue pregnancy was made in 60.2% of cases (502/834) based on negative ES results and severity and treatability of fetal anomalies. In total, ES results showed an overall clinical impact of 61.5% (648/1053) on decision making regarding termination or continuation of pregnancy in the prospective cohort.

**Table 4:** Clinical impacts of ES results in the prospective cohort

Clinical impacts	No. of cases	Percentage (%)
**Diagnostic cases**	**131**	**12.4 (131/1053)**
1. Termination	105	80.2 (105/131)
(1) Decision making on termination due to positive ES result	78	74.3 (78/105)
(2) Termination due to fetal anomaly prior to ES result	27	25.7 (27/105)
2. Continuation of pregnancy and implication for clinical management	20	15.3 (20/131)
(1) Decision making due to non-neurodevelopmental phenotype	7	35.0 (7/20)
(2) Decision making due to inherited from either parent	4	20.0 (4/20)
(3) Decision making due to non-neurodevelopmental phenotype and inherited from the parent	8	40.0 (8/20)
(4) Initial negative result becoming positive due to new disease gene identified	1	5.0 (1/20)
3. Fetal birth before ES report returned	3 (1 with neonatal demise)	2.3 (3/131)
4. Lost to follow-up	1	0.8 (1/131)
5. In pregnancy	2	1.5 (2/131)
**VUS cases**	**81**	**7.7 (81/1053)**
1. Termination	41	50.6 (41/81)
(1) Termination due to fetal anomaly prior to ES result	28	68.3 (28/41)
(2) Termination due to VUS results and fetal anomaly	12	29.3 (12/41)
(3) Termination due to VUS results predominantly	1	2.4 (1/41)
2. Continuation of pregnancy	33	40.7 (33/81)
(1) Decision making due to VUS result and fetal anomaly	22 (1 with neonatal demise)	66.7 (22/33)
(2) Decision making due to VUS inherited from either parent	10	30.3 (10/33)
(3) Decision making due to precious IVF fetus	1	3.0 (1/33)
3. Lost to follow-up	2	2.5 (2/81)
4. In pregnancy	5	6.2 (5/81)
**Cases of IFs with childhood-onset disease**	**5**	**0.5 (5/1053)**
1. Termination due to fetal anomaly prior to ES result	1	20.0 (1/5)
2. Decision making on continuation of pregnancy due to non-neurodevelopmental phenotype of positive and IF result and implication for clinical management	1	20.0 (1/5)
3. Decision making on termination due to positive and IF results	1	20.0 (1/5)
4. Decision making on continuation of pregnancy due to non-neurodevelopmental phenotype of IF result and implication for clinical management	1	20.0 (1/5)
5. In pregnancy	1	20.0 (1/5)
**Cases of SFs with childhood-onset disease**	**1**	**0.1 (1/1053)**
1. Decision making on continuation of pregnancy and implication for clinical management	1	
**Negative cases**	**834**	**79.2 (834/1053)**
1. Termination due to fetal anomaly prior to ES result	205	24.6 (205/834)
2. Decision making on continuation of pregnancy due to negative result and fetal anomaly severity	502 (7 with neonatal demise and 4 with fetal demise)	60.2 (502/834)
3. Fetal birth before ES report returned	10	1.2 (10/834)
4. Lost to follow-up	64	7.7 (64/834)
5. In pregnancy	53	6.4 (53/834)

## Discussion

This study summarizes the clinical experience in implementing pES based on a hospital multidisciplinary team model. The care of a fetal patient is becoming more personalized and precise as prenatal diagnosis gets more sophisticated. Prenatal imaging, maternal serum screening, genetic analysis, and multidisciplinary collaboration promote the accurate identification of fetal abnormalities. The multidisciplinary model of fetal medicine, based on the analysis and opinions of experts from different specialties, makes consensus recommendations on the management of specific high-risk pregnancies or complicated fetal conditions. Not only evidence-based prenatal protocols can be adjusted and developed, but also multidisciplinary teams can coordinate follow-up in early childhood, especially in patients treated with prenatal invasive intervention, thus improving the patient’s prognosis [2, 24]. This study is the first time that an MDT with a stepwise framework analyzed and interpreted prenatal ES data in a large cohort of 1618 fetal cases and assessed the clinical impact of prenatal ES.

It is well known that ES is typically a phenotype-driven test. Despite the advances in prenatal imaging, the fetal genotype-phenotype correlation remains the major practical challenge of prenatal ES. Currently, there is no fetal variant database equivalent to postnatal ones. Variable presentations of fetal disorders and the appearance of ultrasound abnormalities at late gestational age can only complicate the pES data interpretation [25]. Furthermore, some phenotypes are impossible to determine from prenatal imaging, such as developmental delay, intellectual disability, metabolic abnormality, and subtle dysmorphic features. A previous study focused on fetuses with multiple congenital anomalies, using a genotype-first approach followed by reverse phenotyping, shed light on unexpected fetal phenotype-genotype correlations [26].

In our study, we performed the variant analysis in three steps. The genotype-driven followed by phenotype-driven and reanalysis approach identified diagnostic variants in 81.7% (187/229), 12.2% (28/229), and 6.1% (14/229) positive cases in each step, respectively. Twenty-six genes were revealed to expand the spectrum of fetal phenotypes not reported previously. Twenty-seven genes were firstly reported in prenatal cases. Variants in 4 cases (1.7%) were initially considered as incidental findings and reclassified as diagnostic results based on literature searching for similar phenotypes as atypical clinical symptoms. For case 225 with supraventricular tachycardia, enlarged cisterna magna, ascites, and pleural effusion, a de novo nonsense variant was identified in the *CSNK2A1* gene with very vague HPO “match” for cardiovascular system defects. Postnatal examination after termination showed dysmorphic features including hypertelorism, low-set ears, open mouth, and camptodactyly of a finger, which confirmed the genotype-phenotype correlation. The above data further demonstrate the importance and necessity of “real-time” refining of phenotypic information for exome data interpretation, improving diagnostic performance and facilitating identification of novel genotype-phenotype associations [27, 28]. Therefore, it is recommended that a multidisciplinary team with full communication and interaction will improve prenatal ES detection efficiency and medical quality control [29, 30].

The mean number of closely reviewed variants was 1.7, and the average time to review candidate variants was approximately 15 min for each pES trio in our cohort. More than 50% of cases have only one or no candidate variants for close review (median = 1). These data demonstrated that the stepwise analysis strategy in our study was highly efficient and time-saving.

Previous reports highlighted the benefits of implementing ES and regular reanalysis in the clinical setting, with additional 5–22% yields by reanalysis [31]. In our study, data reanalysis only yielded an overall increased diagnostic rate of 0.9%. The possible reasons for the significantly lower yield of reanalysis may include (1) firstly, data reanalysis was only performed on those with additional new phenotypes and upon the physicians’ request (*n*=593); (2) secondly, our cohorts were all prenatal fetuses, and 35.8% (579/1618) cases chose termination of pregnancy, which is not beneficial to comprehensive phenotyping; (3) thirdly, as most pregnant women returned to their local hospitals for delivery or termination, making detailed postpartum phenotype collection difficult; (4) finally, one main limitation of our study is the lack of regular and long-term postnatal follow-up, which needs to be further improved and supplemented.

By ES detection, the diagnostic rate in the retrospective cohort (17.3%) was higher than that in the prospective cohort (12.4%). Similar results were obtained in the most recent study comparing prospective (13%, 24/183) and retrospective (29%, 35/120) cohorts of fetal clinical exome sequencing [13]. As ES was performed at the end of the pregnancy in the retrospective cohort, fetal phenotype observation was much more distinct and comprehensive. The detection rate of 8.5% reported in the largest prospective PAGE study [8] was lower than the 12.4% detection rate of the prospective cohort in the present study. pES analysis in the PAGE study mainly focused on 1628 development-related genes, while our pES assessed all known OMIM disease genes plus genes annotated in the Orphanet database. A total of 11 diagnostic genes in our cohort were outside the list in the PAGE study, suggesting a more comprehensive approach can improve the clinical sensitivity of pES.

Among the different malformation categories, the top five positive predictors for monogenetic diseases were skeletal anomalies (30.4%), multiple malformations (25.9%), cardiovascular anomalies (12.8%), central nervous anomalies (12.6%), and facial anomalies (9.7%). Notably, none of the 46 fetuses with anomalies in the chest system achieved molecular diagnosis by pES, suggesting isolated chest malformation is highly unlikely related to a monogenic disease. However, the limited sample size renders further evaluation necessary. Furthermore, the diagnostic rate (48%, 48/100) in the subgroup with significant family history was much higher than the overall detection rate (14.2%), further illustrating the strong genetic background in cases with family history. However, 39 of such cases remained unresolved, half consisting of cases with a family history of cardiovascular malformations (*n*=11) and cleft palate and lip (*n*=9), consistent with the known multifactorial etiology of these structural malformations. For all families with negative pES results but with strong indications for a monogenic cause, more efforts may be needed to reach a molecular diagnosis, including reanalysis over time with new gene discoveries, technical optimizations, and utilization of more advanced diagnostic methods such as whole genome sequencing [32, 33].

Neurodevelopmental involvement is the most prominent condition of concern that impacts the parental decision on pregnancy. Our results showed a high likelihood of adverse neurodevelopmental consequences in diagnosed cases with central nervous anomalies, FGR, isolated hydrops, multisystem anomalies, cardiovascular anomalies, and increased NT. In contrast, diagnosed cases with skeletal, facial, and urogenital anomalies were less associated with neurodevelopmental abnormalities. These results may be helpful for prenatal genetic counseling and need further corroboration from additional cohorts.

For fetuses with increased NT (≥3.5 mm), the detection rate was significantly higher in the associated group than in the isolated group (25.5% vs. 5%, *p*<0.05) in our cohort, consistent with previous pES studies [34]. The pES positive rate in the isolated increased NT group in our cohort (5%) is similar to a previous meta-analysis showing a 4% (95% CI 2–6%) incremental yield in such cases [35]. These data indicate that the diagnostic yield of pES is low for fetuses with truly isolated increased NT once chromosomal abnormalities are excluded. In contrast, increased NT combined with additional anomalies appears to be a positive predictor for a molecular diagnosis (25.5% positive rate), and such fetuses may be prioritized for pES testing.

In our large cohort, 20.0% (27/135) of the diagnostic genes were reported in prenatal cases for the first time; this expanded the phenotypic spectrum of single-gene disorders to the prenatal setting. Current mutation databases such as ClinVar and Human Genetics Mutation Database include limited fetal phenotypic data, making fetal phenotype-genotype correlation extremely challenging. Continuing efforts on the pES data analysis can provide new information regarding the spectrum of anomalies in rare disorders or well-established genetic conditions without known prenatal characteristics [36]. This new information, in turn, can effectively help to clarify the uncertain significance of results. For example, de novo P/LP variants in the *NFIA* gene were identified in two fetuses (cases 4 and 19) with agenesis of the corpus callosum and ventriculomegaly by prenatal ultrasound and brain MRI at the gestational age of 33 and 22 weeks, respectively. The *NFIA*-related autosomal dominant brain malformations with or without urinary tract defects disorder (BRMUTD; OMIM: 613753) is quite rare, with intragenic mutations described in ~20 affected individuals so far [37]. Central nervous system abnormalities for BRMUTD mainly comprise agenesis or hypoplasia of the corpus callosum, and additional features include macrocephaly, seizures, ventriculomegaly, and hypotonia. NFIA was also considered as the critical gene for 1p32-p31 deletion syndrome. NFIA haploinsufficiency has been associated with ventriculomegaly, corpus callosum hypogenesis, abnormal external genitalia, and intrauterine growth restriction in a fetus [38]. Here, we firstly reported two fetal cases with an *NFIA* intragenic mutation and CNS structural anomalies highly consistent with the postnatal presentations of BRMUTD and the prenatal features for 1p32-p31 deletion syndrome, reinforcing the genotype-prenatal phenotype association for this extremely rare disorder.

The uncertainty of the VUSes identified in the fetuses can result in significant anxiety and make decision making challenging for the parents. Effective resolution strategies include family segregation analysis, confirmatory clinical test / tracing phenotypic clues, case matching by genotype, and functional validation [39]. Family segregation analysis is the most convenient to carry out in clinical practice; however, it is not helpful for the de novo VUS, comprising 29.8% (39/131) of inconclusive results in prenatal cases. Due to the time urgency of prenatal diagnosis, it is usually challenging to implement functional validation for ongoing pregnancy. For case 35 in our retrospective cohort, cleft lip and palate were presented in the fetus, mother, and grandfather. A maternally inherited splicing variant c.1920+1G>A was detected in the *ARHGAP29* gene, which is not a known OMIM disease gene, but recorded in the Orphanet database for nonsyndromic cleft lip and palate (ORPHA:199306). After termination, the mRNA level of ARHGAP29 from samples of the fetus and mother was tested and demonstrated significantly decreased compared to the wild type. RT-PCR analysis revealed the variant caused abnormal skipping of exon 17 in the *ARHGAP29* gene [40]. Phenotypic tracing and clarification is another critical approach, which needs close collaboration between the laboratory and clinicians. Detailed descriptions of phenotypes in fetuses and family members, including differences in clinical presentations, even if subtle or atypical, are important and should be communicated [27]. When pregnancies are ended, postnatal examination by experts in dysmorphology and fetal autopsy at post-mortem can be beneficial to refine the phenotype and target specific genes for further in-depth investigation [25]. Furthermore, long-term postnatal follow-up is also critical to ascertain the individual’s clinical situation and provide proper prenatal counseling.

Compared to the smaller sample size of previous studies on the clinical impact of prenatal ES [41–44], in our large retrospective cohort, 17.3% (98/565) cases obtained molecular diagnoses that guide precise recurrence risk assessment and reproductive planning. In the prospective cohort, positive ES results contributed to decision making on termination or continuation of pregnancy in 74.0% of diagnostic cases (97/131). It has been reported that the overall frequency of unsolicited findings (unrelated to the clinical question) in clinical whole-exome sequencing is low [45, 46]. In our prenatal cohort, the detection rate of unexpected findings, including incidental and secondary findings, was 1.3% (21/1618). Guided by previous statements from international society [29, 47], incidental and secondary findings with childhood-onset diseases were also reported. In the retrospective cohort, IFs and SFs were reported in 0.5% (3/565) cases with live birth, implicating future clinical surveillance and management. Of the prospective cohort, prenatal ES had a clinical impact on 2 cases with IFs and 1 case with SF regarding pregnancy decision making and clinical management. Our experience demonstrated the substantial clinical impact and significant prognostic contribution of ES to pregnancy assessments in the prenatal setting.

Although prenatal ES increased the overall diagnostic yield by 14.2% in our structurally anomalous fetuses with uninformative karyotype and CMA results, significant challenges remain to be overcome when translating ES into clinical practice. Ethical issues that are not unique to prenatal fetal ES detection, such as non-biological parents, the chance of detecting incidental findings, secondary findings, and variants of uncertain significance, can be managed through consistent laboratory principles and a multidisciplinary system [48, 49]. Prenatal ES data analysis and interpretation within the hospital will facilitate continuous communications and confirmations by the multidisciplinary team, further enhancing confidence in the clinical management of such complicated cases. In addition, before applying prenatal ES into routine clinical practice, policies and procedures ensuring patient privacy and confidentiality need to be clarified [47].

## Conclusions

In conclusion, our data, the largest pES cohort so far, showed that most (81.7%) of the causative genes and variants could be captured by genotype-driven analysis prioritizing a short list independent of phenotypic information followed by focused clinical correlation and variant interpretation. Herein, the proven efficient genotype-driven approach enabled rapid analysis through minimizing the most challenging and time-consuming clinical correlation part of pES analysis. Such assumption agnostic strategy also identified potential novel genotype-prenatal phenotype association in many gene/disorders. Findings from pES, especially informed adverse neurodevelopment outcome risk assessment based on pES results, impacted the clinical decision regarding termination vs. continuation of the pregnancy in >60% of couples in the prospective cohort. Our study demonstrated that pES clearly improves existing prenatal diagnostic capabilities, expands our understanding of genetic disease in utero, and thus helps us to better interpret fetal phenotypes in the future. The data presented here affirm the compelling evidence for applying ES as a very promising technique in prenatal genetic diagnosis, especially for fetuses with multiple organ/skeletal abnormalities/positive family histories, and highlight the necessity of establishing a multidisciplinary consultation system to implement prenatal ES.

## Supplementary Information

**Additional file 1.** Analysis and interpretation process of fetal ES data.

**Additional file 2: Table S1.** Clinical characteristics of the fetal cases. **Table S2.** Fetuses with diagnostic or VUS results obtained additional new phenotypes during prenatal and/or postnatal period. **Table S3.** Fetuses with positive diagnostic results detected by ES. **Table S4.** Diagnostic rates in different malformation subgroups. **Table S5.** Diagnostic rates in relation to NT measurement range. **Table S6.** Number of variants analyzed in step 1 and 2- case by case. **Table S7.** Number of variants analyzed in step 1 and 2 based on malformation classification or overall result category. **Table S8.** Fetuses with VUS results detected by ES. **Table S9.** Fetuses with IFs and SFs results detected by ES. **Table S10.** Candidate genes identified in this study. **Table S11.** Pregnancy outcomes of the study cohort.

## Data Availability

The datasets supporting the major results/conclusions of this article are included within the article and its additional files. The genetic variation data in this study are deposited and accessible at the CNGBdb website with accession number CVAR0000192 (https://db.cngb.org/search/variant/CVAR0000192/). These data have also been submitted to GSA and are awaiting data release. Our sequencing raw data cannot be submitted to publicly available databases because the ethical approval did not permit sharing of exome sequencing data and the patients’ families did not consent to share their raw data.

## Abbreviations

ACMG: American College of Medical Genetics and Genomics
ANOVA: Analysis of variance
CA: Congenital anomaly
CMA: Chromosome microarray analysis
ES: Exome sequencing
FGR: Fetal growth retardation
HGMD: Human Gene Mutation Database
HPO: Human Phenotype Ontology
IFs: Incidental findings
MLPA: Multiplex ligation-dependent probe amplification
NT: Nuchal translucency
OMIM: Online Mendelian Inheritance in Man
SFs: Secondary findings
SNVs: Single-nucleotide variants
VUS: Variant of unknown significance
