# Supplementary material for: Application of exome sequencing for prenatal diagnosis of fetal structural anomalies: clinical experience and lessons learned from a cohort of 1618 fetuses
Source: Genome Med. 2022 Oct 28;14:123. doi: 10.1186/s13073-022-01130-x (PMC9615232; doi:10.1186/s13073-022-01130-x)
Supplement: Supplementary file 2 — Additional file 2: Table S1. Clinical characteristics of the fetal cases. Table S2. Fetuses with diagnostic or VUS results obtained additional new phenotypes during prenatal and/or postnatal period. Table S3. Fetuses with positive diagnostic results detected by ES. Table S4. Diagnostic rates in different malformation subgroups. Table S5. Diagnostic rates in relation to NT measurement range. Table S6. Number of variants analyzed in step 1 and 2- case by case. Table S7. Number of variants analyzed in step 1 and 2 based on malformation classification or overall result category. Table S8. Fetuses with VUS results detected by ES. Table S9. Fetuses with IFs and SFs results detected by ES. Table S10. Candidate genes identified in this study. Table S11. Pregnancy outcomes of the study cohort. [file 13073_2022_1130_MOESM2_ESM.docx]

**Additional file 2 Table S1. Clinical characteristics of the fetal cases**

| **Maternal age (median)** | 29 (range 18-47) |
| --- | --- |
| **Gestational weeks (median)** | 25 (range 11-35) |
| **Sex of fetuses (M/F)** | 1001 / 617 |
| **Cases in retrospective/prospective study** | 565 / 1053 |
| **Sample types** |  |
| Chorionic villus | 139 |
| Amniotic fluid | 971 |
| Cord blood | 508 |
| **Malformation classification** |  |
| Central nervous | 262 |
| Facial | 124 |
| Chest | 46 |
| Cardiovascular | 289 |
| Abdominal | 57 |
| Urogenital | 185 |
| Skeletal | 207 |
| FGR | 61 |
| Isolated Hydrops | 29 |
| Increased NT and cystic hygroma | 121 |
| Others | 21 |
| Multisystem | 216 |
| **Total** | **1618** |

Others: fetus with absence of nasal bone and mass abnormalities such as lymphangioma, hemangioma or tumor.

**Additional file 2 Table S2. Fetuses with diagnostic or VUS results obtained additional new phenotypes during prenatal and/or postnatal period**

| **Case ID** | **Gender** | **Initial fetal phenotype** | **Gene(OMIM ID)** | **Transcript** | **Nucleotide change** | **Amino acid change** | **Variant type** | **Zygosity** | **Classification** | **Origin** | **Inheritance** | **Disease(OMIM ID)** | **Pregnancy outcome** | **Group** | **Additional phenotype in prenatal period** | **Additional phenotype in postnatal period** |
| --- | --- | --- | --- | --- | --- | --- | --- | --- | --- | --- | --- | --- | --- | --- | --- | --- |
| **Positive diagnostic cases** | | |  |  |  |  |  |  |  |  |  |  |  |  |  |  |
| **4** | female | Agenesis of corpus callosum, Ventriculomegaly | NFIA(600727) | NM_001145512.1 | c.1112C>A | p.(Ser371Ter) | nonsense | Het | P | De novo | AD | BRAIN MALFORMATIONS WITH OR WITHOUT URINARY TRACT DEFECTS(613735) | Termination | Retrospective |  | Autopsy: Cerebral edema,Cortical dysplasia,Pleural effusion, Ascites |
| **8** | female | Subependymal cysts, Ventriculomegaly | CHD4(603277) | NM_001273.3 | c.2960A>G | p.(Lys987Arg) | missense | Het | LP | De novo | AD | SIFRIM-HITZ-WEISS SYNDROME(617159) | Live birth | Retrospective |  | Subarachnoid hemorrhage,Subdural hemorrhage |
| **9** | male | Dandy-Walker malformation | CSPP1(611654) | NM_024790.6 | c.1641_1642insA | p.(Val548SerfsTer2) | frameshift | Het | P | Pat | AR | JOUBERT SYNDROME 21(615636) | Live birth | Retrospective |  | Subdural hemorrhage, Global developmental delay |
|  |  |  |  | NM_024790.6 | c.3127C>T | p.(Arg1043Ter) | nonsense | Het | P | Mat |  |  |  |  |  |  |
| **11** | female | Hydrocephalus | ANKRD11(611192) | NM_013275.5 | c.1903_1907delAAACA | p.(Lys635GlnfsTer26) | frameshift | Het | P | De novo | AD | KBG SYNDROME(148050) | Live birth | Retrospective |  | Torticollis, Plagiocephaly, Subependymal cysts, Patent foramen ovale, Hypoplasia affecting the anterior segment of the eye ,Growth delay |
| **19 ^b^** | male | Increased nuchal translucency | NFIA(600727) | NM_001145512.1 | c.483A>C | p.(Arg161Ser) | missense | Het | LP | De novo | AD | BRAIN MALFORMATIONS WITH OR WITHOUT URINARY TRACT DEFECTS(613735) | Termination | Prospective | Agenesis of corpus callosum, Ventriculomegaly |  |
| **21 ^b^** | male | Increased nuchal translucency | ADNP(611386) | NM_015339.4 | c.2161C>T | p.(Gln721Ter) | nonsense | Het | P | De novo | AD | HELSMOORTEL-VAN DER AA SYNDROME(615873) | Termination | Prospective | Ventriculomegaly |  |
| **24 ^b^** | male | Increased nuchal translucency | FGFR2(176943) | NM_000141.4 | c.1052C>G | p.(Ser351Cys) | missense | Het | P | De novo | AD | APERT SYNDROME(101200) | Termination | Prospective | Hypertelorism, Ventriculomegaly |  |
| **34** | female | Micrognathia, Polyhydramnios | COL11A1(120280) | NM_080629.2 | c.3449G>T | p.(Gly1150Val) | missense | Het | P | De novo | AD | MARSHALL SYNDROME (154780) | Live birth | Retrospective |  | Cleft palate, Pierre-Robin sequence, Subependymal cysts, Atrial septal defect |
| **38** | male | Micrognathia, Cleft palate | KCNK9(605874) | NM_001282534.1 | c.706G>C | p.(Gly236Arg) | missense | Het | LP | De novo | AD | BIRK-BAREL SYNDROME(612292) | Live birth | Retrospective |  | Pierre-Robin sequence, Atrial septal defect, Cryptorchidism, Anemia |
| **39** | female | Increased nuchal translucency | FOXC2(602402) | NM_005251.2 | c.122_123insGACA | p.(Tyr41Ter) | nonsense | Het | LP | Mat | AD | LYMPHEDEMA-DISTICHIASIS SYNDROME(153400) | Live birth | Prospective | Hypertelorism |  |
| **48** | male | Tetralogy of Fallot, Mitral regurgitation, Tricuspid regurgitation | CHD7(608892) | NM_017780.3 | c.2176_2177delGA | p.(Asp726LeufsTer12) | frameshift | Het | P | De novo | AD | CHARGE SYNDROME(214800) | Neonatal death | Retrospective |  | Pulmonary hemorrhage, Congenital laryngeal stridor, Abnormal bronchus morphology, Polydactyly, External ear malformation, Hydrocele testis, Anemia |
| **52** | male | Persistent left superior vena cava, Ventriclular septal defect | SMARCA4(603254) | NM_001128849.1 | c.3460C>T | p.(Leu1154Phe) | missense | Het | LP | De novo | AD | COFFIN-SIRIS SYNDROME 4(614609) | Neonatal death | Retrospective |  | Atrial septal defect, Pierre-Robin sequence, Hearing impairment, Laryngomalacia, Prolonged prothrombin time, Torticollis, Patent ductus arteriosus |
| **53** | male | Ventricular septal defect, Pulmonary artery stenosis | ZEB2(605802) | NM_014795.3 | c.1200T>A | p.(Tyr400Ter) | nonsense | Het | P | De novo | AD | MOWAT-WILSON SYNDROME(235730) | Live birth | Retrospective |  | Aganglionic megacolon, Growth delay |
| **54** | female | Ventricular septal defect, Cardiac enlargement | FLNA(300017) | NM_001110556.1 | c.7757-2A>C | - | splice_acceptor | Het | LP | De novo | XL | MELNICK-NEEDLES SYNDROME(309350) | Live birth | Retrospective |  | Atrial septal defect, Patent ductus arteriosus, Muscular hypotonia, Pulmonary arterial hypertension |
| **55** | female | Pulmonic stenosis | SHOC2(602775) | NM_007373.3 | c.4A>G | p.(Ser2Gly) | missense | Het | P | De novo | AD | NOONAN SYNDROME-LIKE DISORDER WITH LOOSE ANAGEN HAIR 1(607721) | Live birth | Retrospective |  | Atrial septal defect, Failure to thrive, Global developmental delay |
| **58** | male | Coarctation of aorta | KMT2D(602113) | NM_003482.3 | c.12522_12523delinsCCCCAGG | p.(Lys4174AsnfsTer43) | frameshift | Het | P | De novo | AD | KABUKI SYNDROME 1(147920) | Live birth | Retrospective |  | Mitral stenosis, Hypoplastic aortic arch |
| **60** | female | Dilated cardiomyopathy, Ventricular septal defect | MYH7(160760) | NM_000257.3 | c.2330G>A | p.(Arg777Lys) | missense | Het | LP | De novo | AD | CARDIOMYOPATHY, DILATED, 1S(613426) | Live birth | Retrospective |  | Noncompaction cardiomyopathy, Mitral regurgitation, Tricuspid regurgitation, Pulmonary arterial hypertension |
| **61** | male | Noncompaction cardiomyopathy | NONO(300084) | NM_007363.4 | c.1096C>T | p.(Gln366Ter) | nonsense | Hemi | P | De novo | XL | MENTAL RETARDATION, X-LINKED, SYNDROMIC 34(300967) | Live birth | Retrospective |  | Patent ductus arteriosus, Pulmonary arterial hypertension, Patent foramen ovale |
| **65** | female | Transposition of the great arteries | MAPK1(176948) | NM_002745.4 | c.1061T>G | p.(Phe354Cys) | missense | Het | P | De novo | AD | Noonan syndrome 13(619087) | live birth | Prospective |  | Growth delay |
| **80** | male | Hypoplasia of right ventricle | MYH7(160760) | NM_000257.3 | c.2779G>A | p.(Glu927Lys) | missense | Het | LP | De novo | AD | CARDIOMYOPATHY, FAMILIAL HYPERTROPHIC, 1(192600) | Live birth | Prospective |  | Pulmonary arterial hypertension |
| **81** | female | Increased nuchal translucency | LZTR1(600574) | NM_006767.3 | c.593+2T>C | - | splice_donor | Het | LP | Pat | AR | NOONAN SYNDROME 2(605275) | Termination | Prospective | Cystic hygroma, Hypoplastic left heart |  |
|  |  |  |  | NM_006767.3 | c.1785+1G>A | - | splice_donor | Het | LP | Mat |  |  |  |  |  |  |
| **83** | female | Pyloric stenosis, Gastrectasia | FOXF1(601089) | NM_001451.2 | c.231C>G | p.(Phe77Leu) | missense | Het | LP | De novo | AD | ALVEOLAR CAPILLARY DYSPLASIA WITH MISALIGNMENT OF PULMONARY VEINS(265380) | Neonatal death | Retrospective |  | Pulmonary arterial hypertension |
| **90** | male | Micropenis, Hypospadias | SRD5A2(607306) | NM_000348.3 | c.680G>A | p.(Gly227Glu) | missense | Het | P | Mat | AR | PSEUDOVAGINAL PERINEOSCROTAL HYPOSPADIAS(264600) | Live birth | Retrospective |  | Inguinal hernia |
|  |  |  |  | NM_000348.3 | c.16C>T | p.(Gln6Ter) | nonsense | Het | P | Pat |  |  |  |  |  |  |
| **99** | male | Megacystis | ACTG2(102545) | NM_001615.3 | c.770G>A | p.(Arg257His) | missense | Het | P | Mat | AD | VISCERAL MYOPATHY 1(155310) | Live birth | Prospective |  | Congenital posterior urethral valve, Hydronephrosis, Urinary retention, Hydroureter |
| **100** | female | Increased nuchal translucency | FGFR3(134934) | NM_000142.4 | c.742C>T | p.(Arg248Cys) | missense | Het | P | De novo | AD | ACHONDROPLASIA(100800) | Termination | Retrospective | Short long bone, Bowing of the long bones, Narrow chest, Abnormal skull morphology |  |
| **111** | female | Short long bone | FGFR3(134934) | NM_001163213.1 | c.1144G>A | p.(Gly382Arg) | missense | Het | P | De novo | AD | ACHONDROPLASIA(100800) | Live birth | Retrospective |  | Short stature |
| **113** | male | Short long bone | GNPTAB(607840) | NM_024312.4 | c.1284+1G>T | - | splice_donor | Het | P | Pat | AR | MUCOLIPIDOSIS III ALPHA/BETA (252600) | Live birth | Retrospective |  | Growth delay, increased mucopolysacchariduria |
|  |  |  |  | NM_024312.4 | c.1090C>T | p.(Arg364Ter) | nonsense | Het | P | Mat |  |  |  |  |  |  |
| **115** | male | Hand clenching, Clinodactyly of hallux, Talipes equinovarus | TNNI2(191043) | NM_003282.3 | c.499_501delGAG | p.(Glu167del) | inframe_deletion | Het | LP | Pat(affected) | AD | ARTHROGRYPOSIS, DISTAL, TYPE 2B1(601680) | Live birth | Retrospective |  | Fava bean-induced hemolytic anemia, Motor delay |
| **120** | female | Arthrogryposis multiplex congenita | ASXL3(615115) | NM_030632.2 | c.3039+1G>T | - | splice_donor | Het | P | De novo | AD | BAINBRIDGE-ROPERS SYNDROM(615485) | Live birth | Retrospective |  | Psychomotor retardation, Abnormal facial shape, Hearing impairment |
| **121** | female | Short femur | TRPV4(605427) | NM_021625.4 | c.2353T>C | p.(Trp785Arg) | missense | Het | LP | De novo | AD | PARASTREMMATIC DWARFISM(168400) | Live birth | Retrospective |  | Rickets, Genu varum |
| **126** | male | Hand polydactyly,decreased fetal movement, FGR | ATRX(300032) | NM_000489 | 1035bp deletion | - | Deletion | Hemi | LP | Mat | XR | MENTAL RETARDATION-HYPOTONIC FACIES SYNDROME, X-LINKED, 1(309580) | Live birth | Retrospectie |  | Global developmental delay, Microcephaly, Low-set ears, Hypertelorism, Adducted thumb, Hearing impairment |
| **148** | male | Increased nuchal translucency | FGFR3(134934) | NM_001163213.1 | c.1954A>G | p.(Lys652Glu) | missense | Het | P | De novo | AD | ACHONDROPLASIA(100800) | Termination | Prospective | Macrocephaly, Narrow chest |  |
| **149 ^b^** | male | Increased nuchal translucency | COL2A1(120140) | NM_001844.4 | c.2717G>A | p.(Gly906Glu) | missense | Het | LP | De novo | AD | ACHONDROGENESIS, TYPE II(200610) | Termination | Prospective | Micromelia |  |
| **158 ^b^** | female | Increased nuchal translucency | COL2A1(120140) | NM_001844.4 | c.2213G>T | p.(Gly738Val) | missense | Het | LP | De novo | AD | ACHONDROGENESIS, TYPE II(200610) | Termination | Prospective | Micrognathia, Talipes equinovarus, Short femur |  |
| **163** | male | Intrauterine growth retardation | KAT6A(601408) | NM_006766.4 | c.751C>T | p.(Arg251Ter) | nonsense | Het | P | De novo | AD | ARBOLEDA-THAM SYNDROME(616268) | Live birth | Retrospective |  | Syndactyly |
| **167** | male | Increased nuchal translucency | SETD5(615743) | NM_001080517.2 | c.2476+1G>A | - | splice_donor | Het | P | De novo | AD | MENTAL RETARDATION, AUTOSOMAL DOMINANT 23(615761) | Live birth | Retrospective |  | Gastroesophageal reflux, Growth delay |
| **170** | male | Increased nuchal translucency | KCNT1(608167) | NM_020822.2 | c.1420C>T | p.(Arg474Cys) | missense | Het | P | De novo | AD | DEVELOPMENTAL AND EPILEPTIC ENCEPHALOPATHY 14(614959) | Termination | Prospective | Bilateral choroid plexus cysts, Cystic hygroma |  |
| **173** | male | Increased nuchal translucency | RAF1(164760) | NM_002880.3 | c.770C>T | p.(Ser257Leu) | missense | Het | P | De novo | AD | NOONAN SYNDROME 5(611553) | Neonatal death | Retrospective | Ventriculomegaly, Abnormal posturing of hand, Micropenis,Cystic hygroma | Ileus, Laryngomalacia, Neonatal respiratory distress |
| **188** | female | Micrognathia, Ventricular septal defect | FGFR2(176943) | NM_022970.3 | c.833G>T | p.(Cys278Phe) | missense | Het | P | De novo | AD | APERT SYNDROME(101200) | Live birth | Retrospective |  | Pierre-Robin sequence, Dyspnea, Feeding difficulties, Proptosis |
| **189** | male | Micrognathia, Arthrogryposis multiplex congenita, FGR | UBA1(314370) | NM_153280.2 | c.1617G>T | p.(Met539Ile) | missense | Hemi | LP | Mat | XR | SPINAL MUSCULAR ATROPHY, X-LINKED 2(301830) | Live birth | Retrospective |  | Dyspnea, Hypertonia, Congenital conductive hearing impairment, Congenital hip dislocation, Cryptorchidism, Cubitus valgus, Abnormality of the ankles |
| **190** | female | Ventricular septal defect, Cleft palate | KMT2D(602113) | NM_003482.3 | c.15604delG | p.(Ala5202ProfsTer41) | frameshift | Het | P | De novo | AD | KABUKI SYNDROME 1(147920) | Live birth | Retrospective |  | Pierre-Robin sequence |
| **202** | male | Increased nuchal translucency | RAF1(164760) | NM_002880.3 | c.770C>T | p.(Ser257Leu) | missense | Het | P | De novo | AD | NOONAN SYNDROME 5(611553) | Termination | Prospective | Ventricular septal defect,Holoprosencephaly,Hypertelorism |  |
| **207 ^a^** | male | Urachal cyst, Omphalocele | CRB2(609720) | NM_173689.6 | c.3307T>C | p.(Cys1103Arg) | missense | Het | LP | Mat | AR | VENTRICULOMEGALY WITH CYSTIC KIDNEY DISEASE(219730) | Termination | Prospective | Hyperechogenic kidneys, Ventriculomegaly |  |
|  |  |  |  | NM_173689.6 | c.3548T>A | p.(Leu1183Ter) | nonsense | Het | LP | Pat |  |  | - |  |  |  |
| **208 ^b^** | male | Ventricular septal defect | RET(164761) | NM_020975.4 | c.3148C>T | p.(Arg1050Ter) | nonsense | Het | LP | Pat(affected) | AD | MULTIPLE ENDOCRINE NEOPLASIA, TYPE IIA(171400) | Live birth | Prospective | Ectopic kidney |  |
| **215** | male | Increased nuchal translucency | FLNA(300017) | NM_001110556.1 | c.1538_1539 delGGinsTA | (p.Gly513Val) | inframe | Hemi | LP | Mat | XD | MELNICK-NEEDLES SYNDROME(309350) | Termination | Prospective | Ascites, Hyperechogenic kidneys, Tricuspid regurgitation |  |
| **221 ^b^** | male | Aplasia of the nasal bone, Abnormality of ductus venosus blood flow, Single umbilical artery | ARID1A(603024) | NM_006015.4 | c.5853dupC | p.(Ile1952HisfsTer11) | missense | Het | P | De novo | AD | COFFIN-SIRIS SYNDROME 2(614607) | Termination | Prospective | Ventriculomegaly, Dandy-Walker malformation, Congenital diaphragmatic hernia |  |
| **222** | male | Ventriculomegaly, Frontal bossing, Lemon sign | FGFR2(176943) | NM_000141.4 | c.1019A>G | p.Try340Cys | missense | Het | P | De novo | AD | APERT SYNDROME(101200) | Termination | Prospective | Talipes equinovarus,Renal agenesis, Aplasia/Hypoplasia involving the vertebral column |  |
| **225** | male | Supraventricular tachycardia, Enlarged cisterna magna, Ascites, Pleural effusion, Polyhydramnios | CSNK2A1(115440) | NM_001895.3 | c.838C>T | p.(Arg280Ter) | nonsense | Het | LP | De novo | AD | OKUR-CHUNG NEURODEVELOPMENTAL SYNDROME(617062) | Termination | Prospective |  | Hypertelorism, Low-set ears, Open mouth, Camptodactyly of finger |
| **227** | female | Omphalocele | CDKN1C(600856) | NM_000076.2 | c.827_828delinsAA | p.(Phe276Ter) | nonsense | Het | P | Mat | AD | BECKWITH-WIEDEMANN SYNDROME; BWS(130650) | Termination | Prospective | Tricuspid regurgitation, Polyhydramnios, Short long bone, Hepatomegaly |  |
|  |  |  |  |  |  |  |  |  |  |  |  |  |  |  |  |  |
| **VUS cases** | |  |  |  |  |  |  |  |  |  |  |  |  |  |  |  |
| **235** | male | Cerebellar vermis hypoplasia | CEP120(613446) | NM_153223.3 | c.2132_2133delAA | p.(Lys711ThrfsTer7) | frameshift | Het | LP | Mat | AR | JOUBERT SYNDROME 31(617761) | Live birth | Retrospective |  | Psychomotor retardation, Congenital horizontal nystagmus |
|  |  |  |  | NM_153223.3 | c.322-8T>C |  | splice_region | Het | VUS-high risk | Pat |  |  |  |  |  |  |
| **239** | female | Microcephaly, FGR | TBCD(604649) | NM_005993.4 | c.1573C>T | p.(Pro525Ser) | missense | Het | VUS-high risk | Mat | AR | ENCEPHALOPATHY, PROGRESSIVE, EARLY-ONSET, WITH BRAIN ATROPHY AND THIN CORPUS CALLOSUM(617193) | Live birth | Prospective |  | Hearing abnormality |
|  |  |  |  | NM_005993.4 | c.2178+5A>G |  | splice_region | Het | VUS | Pat |  |  |  |  |  |  |
| **240 ^c^** | male | Dilation of lateral ventricles, FGR | HSPG2(142461) | NM_005529 | c.11929G>A | p.(Val3977Met) | missense | Het | VUS | Mat | AR | SCHWARTZ-JAMPEL SYNDROME, TYPE 1(255800) | Live birth | Prospective |  | Motor delay |
|  |  |  |  | NM_005529 | c.4627-8G>A | - | splice_region | Het | VUS | Pat |  |  |  |  |  |  |
| **253** | male | Cleft palate, Cleft lip | DHCR7(602858) | NM_001360.2 | c.1060G>A | p.(Ala354Thr) | missense | Het | VUS | Pat | AR | SMITH-LEMLI-OPITZ SYNDROME (270400) | Live birth | Retrospective |  | Failure to thrive |
|  |  |  |  | NM_001360.2 | c.289G>A | p.(Ala97Thr) | missense | Het | VUS | Mat |  |  |  |  |  |  |
| **256** | female | Cleft palate | LRP2(600073) | NM_004525.2 | c.10027C>T | p.(Arg3343Cys) | missense | Het | VUS | Mat | AR | DONNAI-BARROW SYNDROME(222448) | Live birth | Retrospective |  | Short stature, Myopia, Hearing impairment |
|  |  |  |  | NM_004525.2 | c.7306A>G | p.(Thr2436Ala) | missense | Het | VUS | Pat |  |  |  |  |  |  |
| **257** | male | Cleft palate | HUWE1(300697) | NM_031407.5 | c.12067C>T | p.(Arg4023Cys) | missense | Hemi | VUS | Mat | XL | MENTAL RETARDATION, X-LINKED, SYNDROMIC, TURNER TYPE(309590) | Live birth | Retrospective |  | Low-set ears,Depressed nasal bridge, Global developmental delay, Failure to thrive |
| **264 ^c^** | male | Hypoplasia of the lungs | ZIC3(300265) | NM_003413.3 | c.1306C>G | p.(Leu436Val) | missense | Hemi | VUS-high risk | Mat | XR | X-LINKED HETEROTAXY, VISCERAL 1 (306955), X-LINKED VACTERL ASSOCIATION WITH OR WITHOUT HYDROCEPHALUS(314390) | Live birth | Retrospective |  | Hand polydactyly, Torticollis, Scimitar anomaly, Pulmonary arterial hypertension, Atrial septal defect |
| **265** | male | Pulmonary sequestration | STRA6(610745) | NM_001199042.1 | c.436T>C | p.(Phe146Leu) | missense | Het | VUS | Pat | AR | MICROPHTHALMIA, SYNDROMIC 9(601186) | Live birth | Prospective | Fetal pyelectasis |  |
|  |  |  |  | NM_001199042.1 | c.367A>G | p.(Arg123Gly) | missense | Het | VUS | Mat |  |  |  |  |  |  |
| **266** | male | Increased nuchal translucency | RLIM(300379) | NM_016120 | c.1093C>T | p.(R365C) | missense | Hemi | VUS-high risk | De novo | XL | TONNE-KALSCHEUER SYNDROME(300978) | Termination | Prospective | Pleural effusion |  |
| **270** | female | Coarctation of aorta, Ventricular septal defect | SMARCA4(603254) | NM_001128849.1 | c.4795G>A | p.(Glu1599Lys) | missense | Het | VUS | De novo | AD | COFFIN-SIRIS SYNDROME 4(614609) | Live birth | Retrospective |  | Patent foramen ovale, Patent ductus arteriosus, Pulmonary arterial hypertension |
| **275** | female | Double outlet right ventricle, Ventricular septal defect, Coarctation of aorta | NOTCH1(190198) | NM_017617.4 | c.4781G>A | p.(Arg1594Gln) | missense | Het | VUS-high risk | De novo | AD | ADAMS-OLIVER SYNDROME 5(616028) | Live birth | Retrospective |  | Patent ductus arteriosus, Atrial septal defect, Pulmonary arterial hypertension, Subependymal cysts |
| **281 ^c^** | female | Increased nuchal translucency | GDF1(602880) | NM_001492.5 | c.480_481delinsA | p.(Gly161AlafsTer5) | frameshift | Het | VUS | Pat | AD | CONGENITAL HEART DEFECTS, MULTIPLE TYPES(613854) | Termination | Prospective | Congenitally corrected transposition of the great arteries with ventricular septal defect |  |
| **285 ^c^** | female | Ventricular septal defect, Cardiomegaly | MYH7(160760) | NM_000257.3 | c.545C>A | p.(Ala182Glu) | missense | Het | VUS | Pat | AD | CARDIOMYOPATHY, FAMILIAL HYPERTROPHIC, 1(192600) | Live birth | Prospective |  | Noncompaction cardiomyopathy, Pulmonary arterial hypertension, Patent foramen ovale, Mitral regurgitation |
| **289** | male | Hydrops fetalis, Polyhydramnios | EPHB4(600011) | NM_004444.4 | c.2354G>A | p.(Arg785Gln) | missense | Het | VUS-high risk | Mat | AD | LYMPHATIC MALFORMATION 7(617300) | Live birth | Prospective |  | Ventriculomegaly, Choroid plexus cyst |
| **291** | male | Hydrops fetalis | UBR1(605981) | NM_174916.2 | c.4750G>A | p.(Val1584Met) | missense | Het | VUS | Mat | AR | JOHANSON-BLIZZARD SYNDROME(243800) | Neonatal death | Prospective |  | Arrhythmia, Pulmonary hypoplasia, Anemia, Hypocalcemia, Hypomagnesiuria, Intracranial hemorrhage |
|  |  |  |  | NM_174916.2 | c.604G>A | p.(Val202Ile) | missense | Het | VUS | Pat |  |  |  |  |  |  |
| **293** | male | Increased nuchal translucency | ADNP(611386) | NM_015339.4 | c.553T>A | p.(Tyr185Asn) | missense | Het | VUS | De novo | AD | HELSMOORTEL-VAN DER AA SYNDROME(615873) | Termination | Prospective | Hydrops fetalis |  |
| **298** | male | Multicystic kidney dysplasia | PKHD1(606702) | NM_138694.3 | c.10058T>G | p.(Leu3353Arg) | missense | Het | VUS-high risk | Pat | AR | POLYCYSTIC KIDNEY DISEASE 4 WITH OR WITHOUT POLYCYSTIC LIVER DISEASE(263200) | Live birth | Retrospective |  | Enlarged interhemispheric fissure, Splenomegaly, Patent foramen ovale |
|  |  |  |  | NM_138694.3 | c.4798G>A | p.(Gly1600Arg) | missense | Het | VUS-high risk | Mat |  |  |  |  |  |  |
| **301** | male | Hydronephrosis | PGK1(311800) | NM_000291.3 | c.989G>A | p.(Arg330Gln) | missense | Hemi | VUS | Mat | XR | PHOSPHOGLYCERATE KINASE 1 DEFICIENCY(300653) | Live birth | Prospective |  | Hydrocele testis |
| **310 ^c^** | male | Calcaneovalgus deformity | LOXL3(607163) | NM_032603.4 | c.39dupG | p.(Leu14AlafsTer21) | frameshift | Homo | VUS | Mat+Pat | AR | Autosomal recessive Stickler syndrome(ORPHA:250984) | Live birth | Retrospective |  | Severe Myopia, Abnormality of the hip joint |
| **313** | male | Abnormal posturing of hand | ATP7A(300011) | NM_000052.6 | c.4479G>C | p.(Arg1493Ser) | missense | Hemi | VUS | Mat | XR | OCCIPITAL HORN SYNDROME(304150);SPINAL MUSCULAR ATROPHY, DISTAL, X-LINKED 3(300489) | Live birth | Prospective |  | Motor delay |
| **322** | female | Duodenal atresia | PIEZO2(613629) | NM_022068.3 | c.3476G>A | p.(Arg1159Lys) | missense | Het | VUS | De novo | AD | MARDEN-WALKER SYNDROME(248700) | Live birth | Retrospective |  | Myocardial damage |
| **324** | female | Omphalocele | LRP2(600073) | NM_004525.2 | c.11341T>G | p.(Tyr3781Asp) | missense | Het | VUS | Pat | AR | DONNAI-BARROW SYNDROME(222448) | Live birth | Prospective |  | Atrial septal defect, Interrupted inferior vena cava with azygous continuation, Abnormality of the bronchi |
|  |  |  |  | NM_004525.2 | c.9070G>A | p.(Glu3024Lys) | missense | Het | VUS | De novo |  |  |  |  |  |  |
| **328** | male | FGR, Oligohydramnios | FLNB(603381) | NM_001164317.1 | c.3661A>G | p.(Lys1221Glu) | missense | Het | VUS | Mat | AR | SPONDYLOCARPOTARSAL SYNOSTOSIS SYNDROME(272460) | Live birth | Retrospective |  | Growth delay |
|  |  |  |  | NM_001164317.1 | c.7762G>A | p.(Glu2588Lys) | missense | Het | VUS | Pat |  |  |  |  |  |  |
| **329** | female | FGR, Polyhydramnios | DOCK6(614194) | NM_020812.3 | c.5227G>A | p.(Gly1743Ser) | missense | Het | VUS | Pat | AR | ADAMS-OLIVER SYNDROME 2; AOS2(614219) | Live birth | Prospective |  | Bradyopsia |
|  |  |  |  | NM_020812.3 | c.4579C>T | p.(Arg1527Cys) | missense | Het | VUS | Mat |  |  |  |  |  |  |
| **337** | female | Micrognathia, Cleft palate, Ventricular septal defect, Left aortic arch with retroesophageal right subclavian artery, Persistent left superior vena cava | NONO(300084) | NM_007363.4 | c.425G>A | p.(Arg142His) | missense | Het | LP | De novo | XL | MENTAL RETARDATION, X-LINKED, SYNDROMIC 34(300967) | Live birth | Retrospective |  | Feeding difficulties, Growth delay |
| **352** | male | Oligohydramnios, Congenital posterior urethral valve, Hydronephrosis, Hydroureter | FREM2(608945) | NM_207361.5 | c.4396C>T | p.(Arg1466Ter) | missense | Het | LP | Pat | AR | FRASER SYNDROME 2(617666) | Termination | Prospective |  | Finger syndactyly, Narrow palpebral fissure |
|  |  |  |  | NM_207361.5 | c.8347A>T | p.(Ile2783Leu) | missense | Het | VUS | Mat |  |  |  |  |  |  |
| **355 ^c^** | male | Ascites, Oligohydramnios | NPC1(607623) | NM_000271.4 | c.2526T>A | p.(Phe842Leu) | missense | Het | VUS-high risk | Mat | AR | NIEMANN-PICK DISEASE, TYPE C1(257220) | Termination | Prospective | Splenomegaly, Hepatomegaly, Increased placental thickness |  |
|  |  |  |  | NM_000271.4 | c.1226T>C | p.(Ile409Thr) | missense | Het | LP | De novo |  |  |  |  |  |  |
| **356 ^c^** | female | Ascites, Cardiomegaly, Tricuspid regurgitation | TMEM260(617449) | NM_017799.3 | c.107T>C | p.(Val36Ala) | missense | Het | VUS | Mat | AR | STRUCTURAL HEART DEFECTS AND RENAL ANOMALIES SYNDROME (617478) | Termination | Prospective | Agenesis of corpus callosum, Cerebellar hypoplasia, Polyhydramnios, Hydrops fetalis |  |
|  |  |  |  | NM_017799.3 | c.284A>G | p.(Asn95Ser) | missense | Het | VUS | Pat |  |  |  |  |  |  |
| **358 ^c^** | male | Increased nuchal translucency | LZTR1(600574) | NM_006767.3 | c.741C>A | p.(Ser247Arg) | missense | Het | VUS | Mat | AR | NOONAN SYNDROME 2(605275) | Termination | Prospective | Cystic hygroma, Hypertelorism, Pleural effusion, Ascites |  |
|  |  |  |  | NM_006767.3 | c.1349G>A | p.(Gly450Asp) | missense | Het | VUS | Pat |  |  |  |  |  |  |

^a^  The case obtained upgrading from VUS to LP due to additional new phenotypes.

^b^ Seven cases reclassified as diagnostic results due to additional new phenotypes in late pregnancy.

^c^ Eight cases obtained upgrading from negative to VUS due to additional new phenotypes.

**Additional file 2 Table S3. Fetuses with positive diagnostic results detected by ES**

| **Case ID** | **Gender** | **Ultrasound findings** | **Gene(OMIM ID)** | **Transcript** | **Nucleotide change** | **Amino acid change** | **Variant type** | **Zygosity** | **Classification** | **ACMG codes** | **Origin** | **Inheritance** | **Disease(OMIM ID)** | **Pregnancy outcome** | **Group** | **Evidence for causality:** matching HPO entry | **Additional fetal phenotypes** |
| --- | --- | --- | --- | --- | --- | --- | --- | --- | --- | --- | --- | --- | --- | --- | --- | --- | --- |
| **Central nervous** | | |  |  |  |  |  |  |  |  |  |  |  |  |  |  |  |
| **1** | male | Agenesis of corpus callosum, Subependymal cysts | ARX(300382) | NM_139058.2 | c.994C>G** | p.(Arg332Gly) | missense | hemi | LP | PM2, PP3, PM5, PM1_PP | Mat | XL | LISSENCEPHALY, X-LINKED, 2(300215) | Termination | Retrospective | Agenesis of corpus callosum | Subependymal cysts |
| **2** | female | Dandy-Walker malformation | CHD7(608892) | NM_017780.3 | c.7879C>T | p.(Arg2627Ter) | nonsense | Het | P | PVS1, PM2, PS2_PP, PS4_PM | De novo | AD | CHARGE SYNDROME(214800) | Termination | Retrospective | Dandy-Walker malformation | ─ |
| **3** | female | Hydrocephalus | TUBA1A(602529) | NM_006009.3 | c.781C>T** | p.(Pro261Ser) | missense | Het | LP | PM2, PM1_PP, PS2_PP, PP3_PM, PP2 | De novo | AD | LISSENCEPHALY 3(611603) | Termination | Retrospective | Central nervous system; PMID: 26493046,35017693 | ─ |
| **4** | female | Agenesis of corpus callosum, Ventriculomegaly | NFIA(600727)* | NM_001145512.1 | c.1112C>A** | p.(Ser371Ter) | nonsense | Het | P | PVS1, PS2, PM2 | De novo | AD | BRAIN MALFORMATIONS WITH OR WITHOUT URINARY TRACT DEFECTS(613735) | Termination | Retrospective | Agenesis of corpus callosum, Ventriculomegaly | ─ |
| **5** | male | Agenesis of the corpus callosum, Subependymal cysts, Cerebral dysplasia | TUBB2B(612850) | NM_178012.4 | c.538G>A** | p.(Val180Met) | missense | Het | LP | PS2, PM2, PP3, PP2 | De novo | AD | CORTICAL DYSPLASIA, COMPLEX, WITH OTHER BRAIN MALFORMATIONS 7(610031) | Termination | Retrospective | Agenesis of corpus callosum | Subependymal cysts |
| **6** | male | Microcephaly | ASPM(605481) | NM_018136.4 | c.7782_7783delGA | p.(Lys2595SerfsTer6) | frameshift | Het | P | PVS1, PM2_PP, PM3 | Pat | AR | MICROCEPHALY 5, PRIMARY(608716) | Termination | Retrospective | Microcephaly | ─ |
|  |  |  |  | NM_018136.4 | c.1789C>T | p.(Arg597Ter) | nonsense | Het | P | PVS1, PM2, PM3 | Mat |  |  |  |  |  |  |
| **7** | male | Widened posterior fossa | EZH2(601573)* | NM_004456.4 | c.2050C>T | p.(Arg684Cys) | missense | Het | P | PS2, PS3_PM, PS4_PP, PM2, PM5, PP3 | De novo | AD | WEAVER SYNDROME(277590) | Live birth | Retrospective | Central nervous system | Widened posterior fossa |
| **8 ^#^** | female | Subependymal cysts, Ventriculomegaly | CHD4(603277) | NM_001273.3 | c.2960A>G** | p.(Lys987Arg) | missense | Het | LP | PS2_PM, PM1_PP, PM2, PP3, PP2 | De novo | AD | SIFRIM-HITZ-WEISS SYNDROME(617159) | Live birth | Retrospective | Ventriculomegaly | Subependymal cysts |
| **9** | male | Dandy-Walker malformation | CSPP1(611654) | NM_024790.6 | c.1641_1642insA | p.(Val548SerfsTer2) | frameshift | Het | P | PVS1, PM2, PM3 | Pat | AR | JOUBERT SYNDROME 21(615636) | Live birth | Retrospective | Dandy-Walker malformation | ─ |
|  |  |  |  | NM_024790.6 | c.3127C>T | p.(Arg1043Ter) | nonsense | Het | P | PVS1, PM2, PM3 | Mat |  |  |  |  |  |  |
| **10** | male | Ventriculomegaly | ANKRD11(611192) | NM_013275.5 | c.1903_1907delAAACA | p.(Lys635GlnfsTer26) | frameshift | Het | P | PVS1, PM2, PS2, PS4 | De novo | AD | KBG SYNDROME(148050) | Live birth | Retrospective | Ventriculomegaly | ─ |
| **11** | female | Hydrocephalus | ANKRD11(611192) | NM_013275.5 | c.1903_1907delAAACA | p.(Lys635GlnfsTer26) | frameshift | Het | P | PVS1, PM2, PS2, PS4 | De novo | AD | KBG SYNDROME(148050) | Live birth | Retrospective | Central nervous system | ─ |
| **12** | female | Hypoplasia of the corpus callosum, Ventriculomegaly | PPP2R1A(605983)* | NM_014225.5 | c.775G>A | p.(Val259Ile) | missense | Het | LP | PS2, PM2, PP3 | De novo | AD | MENTAL RETARDATION, AUTOSOMAL DOMINANT 36; MRD36(616362) | Live birth | Retrospective | Hypoplasia of the corpus callosum, Ventriculomegaly | ─ |
| **13** | female | Hypoplasia of the corpus callosum | AHDC1(615790) | NM_001029882.2 | c.1759C>T | p.(Arg587Ter) | nonsense | Het | P | PVS1_PS, PS2, PM2 | De novo | AD | XIA-GIBBS SYNDROME(615829) | Termination | Retrospective | Hypoplasia of the corpus callosum | ─ |
| **14** | female | Macrogyria,Lissencephaly | PAFAH1B1(601545) | NM_000430.3 | c.714_722delTATGGTACG** | p.(Met239_Arg241del) | In-frame | Het | LP | PS2, PM2, PM4 | De novo | AD | LISSENCEPHALY 1(607432) | Termination | Retrospective | Lissencephaly | ─ |
| **15** | male | Hypoplasia of the corpus callosum | AP4M1(602296) | NM_004722.3 | c.300delG** | p.(Thr101ProfsTer30) | frameshift | Het | P | PVS1, PM2, PM3 | Mat | AR | SPASTIC PARAPLEGIA 50, AUTOSOMAL RECESSIVE(612936) | Termination | Retrospective | Hypoplasia of the corpus callosum | ─ |
|  |  |  |  | NM_004722.3 | c.1025+4A>T** | - | splice region | Het | LP | PVS1_PM, PM2, PM3 | Pat |  |  |  |  |  |  |
| **16** | male | Porencephalic cyst, Posterior fossa cyst | COL4A1(120130) | NM_001845.5 | c.3556G>A | p.(Gly1186Ser) | missense | Het | LP | PM1, PM2, PS2_PM, PP3 | De novo | AD | BRAIN SMALL VESSEL DISEASE 1 WITH OR WITHOUT OCULAR ANOMALIES(175780) | Termination | Prospective | Porencephalic cyst | Posterior fossa cyst |
| **17** | female | Pachygyria | PAFAH1B1(601545) | NM_000430.3 | c.41_42dupCT** | p.(Ile15LeufsTer2) | frameshift | Het | P | PVS1, PS2_PM, PM2 | De novo | AD | LISSENCEPHALY 1(607432) | Termination | Prospective | Pachygyria | ─ |
| **18** | female | Dilation of lateral ventricles, Polyhydramnios | **ARV1(611647)*** | NM_022786.3 | c.409delG** | p.(Glu137AsnfsTer13) | frameshift | Het | P | PVS1, PM2, PM3 | Pat | AR | EPILEPTIC ENCEPHALOPATHY, EARLY INFANTILE, 38(617020) | Termination | Prospective | Central nervous system | Dilation of lateral ventricles, Polyhydramnios |
|  |  |  |  | NM_022786.3 | c.518dupA | p.(Pro174AlafsTer14) | frameshift | Het | P | PVS1, PM2_PP, PM3 | Mat |  |  |  |  |  |  |
| **19** | male | Increased nuchal translucency, Agenesis of corpus callosum, Ventriculomegaly | NFIA(600727)* | NM_001145512.1 | c.483A>C** | p.(Arg161Ser) | missense | Het | LP | PS2, PM1, PP3 | De novo | AD | BRAIN MALFORMATIONS WITH OR WITHOUT URINARY TRACT DEFECTS(613735) | Termination | Prospective | Agenesis of corpus callosum, Ventriculomegaly | Increased nuchal translucency |
| **20** | male | Hypoplasia of the corpus callosum | ARID1B(614556) | NM_001346813.1 | c.5145+1G>T** | - | splice donor | Het | LP | PVS1_PS, PS2_PM, PM2 | De novo | AD | COFFIN-SIRIS SYNDROME 1(135900) | Termination | Prospective | Hypoplasia of the corpus callosum | ─ |
| **21** | male | Increased nuchal translucency, Ventriculomegaly | ADNP(611386)* | NM_015339.4 | c.2161C>T** | p.(Gln721Ter) | nonsense | Het | LP | PVS1_PS, PS2_PM, PM2 | De novo | AD | HELSMOORTEL-VAN DER AA SYNDROME(615873) | Termination | Prospective | Ventriculomegaly | Increased nuchal translucency |
| **22** | male | Aplasia of the corpus callosum | ARID1B(614556) | NM_001346813.1 | c.5932C>T | p.(Gln1978Ter) | nonsense | Het | LP | PVS1_PS, PS2_PM, PM2 | De novo | AD | COFFIN-SIRIS SYNDROME 1(135900) | Termination | Prospective | Aplasia of the corpus callosum | ─ |
| **23** | male | Gray matter heterotopias, Porencephalic cyst | COL4A1(120130) | NM_001845.5 | c.3034_3041delinsCC** | p.(Ser1012_Gly1014delinsPro) | In-frame | Het | LP | PS2, PM1, PM2 | De novo | AD | BRAIN SMALL VESSEL DISEASE 1 WITH OR WITHOUT OCULAR ANOMALIES(175780) | Termination | Prospective | Porencephalic cyst | ─ |
| **24** | male | Increased nuchal translucency, Hypertelorism, Ventriculomegaly | FGFR2(176943) | NM_000141.4 | c.1052C>G | p.(Ser351Cys) | missense | Het | P | PS2_PVS, PS4, PM2 | De novo | AD | APERT SYNDROME(101200) | Termination | Prospective | Hypertelorism, Ventriculomegaly | ─ |
| **25** | male | Cerebellar vermis hypoplasia, Dilated fourth ventricle, Large foramen magnum | CC2D2A(612013) | NM_001378615.1 | c.2161C>A** | p.(Pro721Thr) | missense | Het | LP | PM2, PM3, PP3_PM, PP4 | Mat | AR | JOUBERT SYNDROME 9(612285) | Termination | Prospective | Cerebellar vermis hypoplasia, Dilated fourth ventricle | ─ |
|  |  |  |  | NM_001378615.1 | c.4465_4468delGACA | p.(Asp1489LysfsTer15) | frameshift | Het | P | PVS1, PM2, PM3, PP4 | Pat |  |  |  |  |  |  |
| **26** | male | Ventriculomegaly | **DLL1(606582)** | NM_005618.3 | c.1407C>A** | p.(Cys469Ter) | nonsense | Het | P | PVS1, PS2_PM, PM2 | De novo | AD | NEURODEVELOPMENTAL DISORDER WITH NONSPECIFIC BRAIN ABNORMALITIES AND WITH OR WITHOUT SEIZURES(618709) | Termination | Prospective | Ventriculomegaly | ─ |
| **27** | female | Microcephaly | PDHA1(300502) | NM_000284.4 | c.1142_1145dupATCA | p.(Trp383SerfsTer6) | frameshift | Het | P | PVS1_PM, PM2, PS2_PVS, PP4 | De novo | AD | PYRUVATE DEHYDROGENASE E1-ALPHA DEFICIENCY(312170) | Termination | Prospective | Microcephaly | ─ |
| **28** | male | Agenesis of corpus callosum | EPG5(615068) | NM_020964.2 | c.3877C>T** | p.(Arg1293Cys) | missense | Het | LP | PM2, PM3, PP3_PM | Pat | AR | VICI SYNDROME(242840) | Termination | Prospective | Agenesis of corpus callosum | ─ |
|  |  |  |  | NM_020964.2 | c.3481C>T | p.(Arg1161Ter) | nonsense | Het | P | PVS1, PM2, PM3_PP | Mat |  |  |  |  |  |  |
| **29** | female | Widened posterior fossa, Arachnoid cyst | FLNA(300017) | NM_001110556.1 | c.4265dupA** | p.(Tyr1422Ter) | frameshift | Het | P | PVS1, PS2_PP, PM2 | De novo | XL | PERIVENTRICULAR NODULAR HETEROTOPIA 1(300049) | Termination | Prospective | PMID: 23151899, 30391507, 25475607 | ─ |
| **30** | male | Hydrocephalus, Hypoplasia of the corpus callosum | PPP2R1A(605983)* | NM_014225.5 | c.544C>T | p.(Arg182Trp) | missense | Het | P | PM1, PM2, PS3_PP, PS2_PVS | De novo | AD | MENTAL RETARDATION, AUTOSOMAL DOMINANT 36(616362) | Termination | Prospective | Hydrocephalus, Hypoplasia of the corpus callosum | ─ |
| **31** | male | Bilateral choroid plexus cyst, Single umbilical artery | **ZMYM2(602221)*** | NM_003453.4 | c.534dupA** | p.(Asp179ArgfsTer3) | missense | Het | P | PVS1, PS2_PP, PM2 | De novo | AD | Neurodevelopmental craniofacial syndrome with variable renal and cardiac abnormalities(619522) | In pregnancy | Prospective | Central nervous system | Bilateral choroid plexus cyst, Single umbilical artery |
| **32** | male | Microcephaly, Intrauterine growth retardation | ASPM(605481) | NM_018136.4 | c.3385A>T** | p.(Lys1129Ter) | nonsense | Het | P | PVS1, PM2, PM3 | Pat | AR | MICROCEPHALY 5, PRIMARY(608716) | Termination | Prospective | Microcephaly | ─ |
|  |  |  |  | NM_018136.4 | c.1761_1762delAG** | p.(Arg587SerfsTer23) | frameshift | Het | P | PVS1, PM2, PM3 | Mat |  |  |  |  |  |  |
| **33** | male | Large for gestational age, Ventriculomegaly | PTCH1(601309) | NM_000264.3 | c.114delG | p.(Leu39CysfsTer41) | frameshift | Het | P | PVS1, PS2_PM, PM2 | Pat(Unknown) | AD | HOLOPROSENCEPHALY 7(601828) | Live birth | Prospective | Large for gestational age, Ventriculomegaly | ─ |
| **Facial** | | |  |  |  |  |  |  |  |  |  |  |  |  |  |  |  |
| **34** | female | Micrognathia, Polyhydramnios, Cleft palate | COL11A1(120280) | NM_080629.2 | c.3449G>T** | p.(Gly1150Val) | missense | Het | LP | PM1, PM2, PS2, PP3 | De novo | AD | MARSHALL SYNDROME (154780) | Live birth | Retrospective | Micrognathia, Cleft palate | ─ |
| **35** | male | Cleft lip,Cleft palate | **ARHGAP29(610496)** | NM_004815.4 | c.1920+1G>A | - | splice donor | Het | P | PVS1, PM2, PP1 | Mat(affected) | AD | Nonsyndromic Cleft Lip and Palate(ORPHA:199306) | Termination | Retrospective | Cleft palate | ─ |
| **36** | male | Cleft lip,Cleft palate | IRF6(607199) | NM_006147.3 | c.181G>T | p.(Ala61Ser) | missense | Het | LP | PS2, PM2, PP3_PM | De novo | AD | VAN DER WOUDE SYNDROME 1(119300) | Termination | Retrospective | Cleft lip, Cleft palate | ─ |
| **37** | female | Cleft lip,Cleft palate | IRF6(607199) | NM_006147.3 | c.439_442delGAAG** | p.(Glu147MetfsTer19) | frameshift | Het | P | PVS1, PM2, PP4 | Pat(affected) | AD | VAN DER WOUDE SYNDROME 1(119300) | Live birth | Retrospective | Cleft lip, Cleft palate | ─ |
| **38** | male | Micrognathia, Cleft palate | KCNK9(605874)* | NM_001282534.1 | c.706G>C | p.(Gly236Arg) | missense | Het | P | PS2_PVS, PS3_PP, PM2 | De novo | AD | BIRK-BAREL SYNDROME(612292) | Live birth | Retrospective | Micrognathia, Cleft palate | ─ |
| **39** | female | Hypertelorism, Increased nuchal translucency | FOXC2(602402) | NM_005251.2 | c.122_123insGACA** | p.(Tyr41Ter) | frameshift | Het | LP | PVS1_PS, PM2, PP4 | Mat(affected) | AD | LYMPHEDEMA-DISTICHIASIS SYNDROME(153400) | Live birth | Prospective | PMID: 32219830, 29866673, 19548265 | ─ |
| **40** | male | Congenital cataract | CRYAA(123580)* | NM_000394.3 | c.34C>T | p.(Arg12Cys) | missense | Het | P | PS4, PM2, PP1_PS, PP3 | Pat(affected) | AD | CATARACT 9, MULTIPLE TYPES; CTRCT9(604219) | Live birth | Prospective | Congenital cataract | ─ |
| **41** | male | Agenesis of nasal bones, Polyhydramnios | RUNX2(600211) | NM_001024630.3 | c.659C>T | p.(Thr220Ile) | missense | Het | LP | PS2_PM, PM2, PP3, PP4 | De novo | AD | CLEIDOCRANIAL DYSPLASIA(119600) | Live birth | Prospective | Abnormal nasal morphology | ─ |
| **42** | male | Cleft palate, Cleft lip | KMT2D(602113) | NM_003482.3 | c.15673C>T | p.(Arg5225Cys) | missense | Het | P | PS2_PVS, PM2, PP3 | De novo | AD | KABUKI SYNDROME 1(147920) | Termination | Prospective | Cleft palate | ─ |
| **43** | female | Micrognathia, Microtia, Hypertelorism, Hypoplasia of the nasal bone, Polyhydramnios | TCOF1(606847) | NM_001135243.1 | c.2286dupT** | p.(Glu763Ter) | frameshift | Het | P | PVS1, PS2, PM2 | De novo | AD | TREACHER COLLINS SYNDROME 1(154500) | Termination | Prospective | Micrognathia, Microtia, Hypertelorism | ─ |
| **44** | female | Unilateral cleft lip, Unilateral cleft palate | SIX3(603714) | NM_005413.3 | c.595delC** | p.(Leu199CysfsTer52) | frameshift | Het | LP | PVS1, PM2 | Mat(affected) | AD | HOLOPROSENCEPHALY 2(157170) | Termination | Prospective | Cleft lip | ─ |
| **45** | female | Cleft palate | EYA1(601653) | NM_000503.5 | c.1081C>T | p.(Arg361Ter) | nonsense | Het | P | PVS1, PS2, PM2 | De novo | AD | BRANCHIOOTORENAL SYNDROME 1(113650) | Termination | Prospective | Cleft palate | ─ |
| **Cardiovascular** | | | |  |  |  |  |  |  |  |  |  |  |  |  |  |  |
| **46** | male | Tetralogy of Fallot | ADNP(611386)* | NM_015339.4 | c.2156dupA | p.(Tyr719Ter) | frameshift | Het | P | PVS1_PS, PS2, PM2 | De novo | AD | HELSMOORTEL-VAN DER AA SYNDROME(615873) | Termination | Retrospective | cardiovascular system | ─ |
| **47** | female | Ventricular septal defect | PTPN11(176876) | NM_002834.3 | c.1510A>G | p.(Met504Val) | missense | Het | P | PS3, PM6_PS, PS4_PM, PP2, PP3 | De novo | AD | NOONAN SYNDROME 1(163950) | Live birth | Retrospective | Ventricular septal defect | ─ |
| **48** | male | Tetralogy of Fallot, Mitral regurgitation, Tricuspid regurgitation | CHD7(608892) | NM_017780.3 | c.2176_2177delGA | p.(Asp726LeufsTer12) | frameshift | Het | P | PVS1, PS2_PP, PM2 | De novo | AD | CHARGE SYNDROME(214800) | Neonatal death | Retrospective | Tetralogy of Fallot | ─ |
| **49** | male | Mitral atresia, Ventricular septal defect | KMT2D(602113) | NM_003482.3 | c.8994dupT | p.(Ala2999CysfsTer8) | frameshift | Het | P | PVS1, PS2_PM, PM2 | De novo | AD | KABUKI SYNDROME 1(147920) | Termination | Retrospective | Ventricular septal defect | ─ |
| **50** | female | Dilatation of the ventricular cavity, Left ventricular pseudoaneurysm | FBN1(134797) | NM_000138.4 | c.6497A>G** | p.(Asp2166Gly) | missense | Het | LP | PS2_PM, PM1, PM2, PM5 | De novo | AD | MARFAN SYNDROME(154700) | Termination | Retrospective | Cardiovascular system | ─ |
| **51** | female | Aortic valve stenosis | PTPN11(176876) | NM_002834.3 | c.166A>G | p.(Ile56Val) | missense | Het | P | PS2, PS4, PM2, PP3, PP2 | De novo | AD | NOONAN SYNDROME 1(163950) | Termination | Retrospective | Cardiovascular system | ─ |
| **52** | male | Persistent left superior vena cava, Ventriclular septal defect | SMARCA4(603254) | NM_001128849.1 | c.3460C>T | p.(Leu1154Phe) | missense | Het | LP | PS2_PM, PM1, PM2, PP3 | De novo | AD | COFFIN-SIRIS SYNDROME 4(614609) | Neonatal death | Retrospective | Cardiovascular system | ─ |
| **53** | male | Ventricular septal defect, Pulmonary artery stenosis | ZEB2(605802) | NM_014795.3 | c.1200T>A** | p.(Tyr400Ter) | nonsense | Het | P | PVS1, PS2_PM, PM2 | De novo | AD | MOWAT-WILSON SYNDROME(235730) | Live birth | Retrospective | Ventricular septal defect | ─ |
| **54** | female | Ventricular septal defect, Cardiac enlargement | FLNA(300017) | NM_001110556.1 | c.7757-2A>C** | - | splice acceptor | Het | LP | PVS1_PM, PS2_PM, PM2 | De novo | XL | MELNICK-NEEDLES SYNDROME(309350) | Live birth | Retrospective | Cardiovascular system | ─ |
| **55** | female | Pulmonic stenosis | SHOC2(602775) | NM_007373.3 | c.4A>G | p.(Ser2Gly) | missense | Het | P | PS2_PVS, PS3, PS4, PM2, PP2 | De novo | AD | NOONAN SYNDROME-LIKE DISORDER WITH LOOSE ANAGEN HAIR 1(607721) | Live birth | Retrospective | Pulmonic stenosis | ─ |
| **56** | male | Tetralogy of Fallot | BRAF(164757) | NM_001374258.1 | c.1575G>C | p.(Leu525Phe) | missense | Het | P | PS4_PM, PS2, PS3, PM2, PP2, PP3 | De novo | AD | CARDIOFACIOCUTANEOUS SYNDROME 1(115150) | Termination | Retrospective | Tetralogy of Fallot | ─ |
| **57** | female | Pulmonic stenosis, Ventricular septal defect | HRAS(190020) | NM_005343.3 | c.34G>A | p.(Gly12Ser) | missense | Het | P | PS2_PVS, PS4, PS3, PM1, PM2, PP2, PP3 | De novo | AD | COSTELLO SYNDROME(218040) | Termination | Retrospective | Pulmonic stenosis, Ventricular septal defect | ─ |
| **58** | male | Coarctation of aorta | KMT2D(602113) | NM_003482.3 | c.12522_12523delinsCCCCAGG** | p.(Lys4174AsnfsTer43) | frameshift | Het | P | PVS1, PS2_PP, PM2 | De novo | AD | KABUKI SYNDROME 1(147920) | Live birth | Retrospective | Coarctation of aorta | ─ |
| **59** | male | Coarctation of aorta, Double outlet right ventricle | KMT2D(602113) | NM_003482.3 | c.12823C>T | p.(Gln4275Ter) | nonsense | Het | P | PVS1, PS2_PM, PM2 | De novo | AD | KABUKI SYNDROME 1(147920) | Termination | Retrospective | Coarctation of aorta | ─ |
| **60** | female | Dilated cardiomyopathy, Ventricular septal defect | **MYH7(160760)** | NM_000257.3 | c.2330G>A | p.(Arg777Lys) | missense | Het | LP | PS2_PM, PM1, PM2, PP3 | De novo | AD | CARDIOMYOPATHY, DILATED, 1S(613426) | Live birth | Retrospective | Dilated cardiomyopathy | ─ |
| **61** | male | Noncompaction cardiomyopathy | NONO(300084) | NM_007363.4 | c.1096C>T** | p.(Gln366Ter) | nonsense | Hemi | LP | PVS1, PS2_PM, PM2 | De novo | XL | MENTAL RETARDATION, X-LINKED, SYNDROMIC 34(300967) | Live birth | Retrospective | Noncompaction cardiomyopathy | ─ |
| **62** | female | Common atrium, Complete atrioventricular canal defect, Coarctation of aorta | EVC2(607261) | NM_147127.4 | c.2335G>T | p.(Glu779Ter) | nonsense | Het | P | PVS1, PM2, PP4 | Mat | AR | ELLIS-VAN CREVELD SYNDROME(225500) | Termination | Prospective | Common atrium | ─ |
|  |  |  |  | NM_147127.4 | c.2965_2967delCTC | p.(Leu989del) | In-frame | Het | LP | PM2, PM3, PM4, PP4 | Pat |  |  |  |  |  |  |
| **63** | male | Cardiac rhabdomyoma | TSC2(191092) | NM_000548.5 | c.880G>A | p.(Gly294Arg) | missense | Het | LP | PM2, PM5, PP3, PP4 | Pat(affected) | AD | TUBEROUS SCLEROSIS 2(613254) | Termination | Prospective | Cardiac rhabdomyoma | ─ |
| **64** | female | Cardiac rhabdomyoma | TSC2(191092) | NM_000548.4 | c.2410T>C | p.(Cys804Arg) | missense | Het | P | PS2_PM, PS3_PP, PM2, PP3 | De novo | AD | TUBEROUS SCLEROSIS 2(613254) | Termination | Prospective | Cardiac rhabdomyoma | ─ |
| **65 ^#^** | female | Transposition of the great arteries | **MAPK1(176948)*** | NM_002745.4 | c.1061T>G | p.(Phe354Cys) | missense | Het | LP | PS2, PM2, PP3, PP2 | De novo | AD | Noonan syndrome 13(619087) | live birth | Prospective | Cardiovascular system | ─ |
| **66** | female | Cardiac rhabdomyoma | TSC2(191092) | NM_000548.4 | c.5238_5255delCATCAAGCGGCTCCGCCA | p.(His1746_Arg1751del) | In-frame | Het | P | PS4, PS3_PM, PM2, PM4 | Mat(affected) | AD | TUBEROUS SCLEROSIS 2(613254) | Termination | Prospective | Cardiac rhabdomyoma | ─ |
| **67** | male | Coarctation of aorta, Pulmonary artery stenosis | KDM6A(300128) | NM_001291415.1 | c.3510_3512delTCT | p.(Leu1171del) | In-frame | Hemi | LP | PS2, PM2, PM4_PP | De novo | XD | KABUKI SYNDROME 2(300867) | Termination | Prospective | Coarctation of aorta | ─ |
| **68** | female | Cardiac rhabdomyoma | TSC2(191092) | NM_000548.4 | c.1831C>T | p.(Arg611Trp) | missense | Het | P | PS2_PM, PS3_PM, PS4, PM2, PP3 | De novo | AD | TUBEROUS SCLEROSIS 2(613254) | Termination | Prospective | Cardiac rhabdomyoma | ─ |
| **69** | male | Neoplasm of the heart | TSC1(605284) | NM_000368.4 | c.2773G>T | p.(Glu925Ter) | nonsense | Het | P | PVS1, PS2_PM, PM2 | De novo | AD | TUBEROUS SCLEROSIS 1(191100) | Live birth | Prospective | Neoplasm of the heart | ─ |
| **70** | male | Mitral regurgitation, Tricuspid regurgitation, Hypoplastic tricuspid valve, Cardiomegaly | **CDH2(114020)** | NM_001792.3 | c.713A>G** | p.(His238Arg) | missense | Het | LP | PS2, PM2, PP3 | De novo | AD | ARRHYTHMOGENIC RIGHT VENTRICULAR DYSPLASIA, FAMILIAL, 14(618920) | Termination | Prospective | Mitral regurgitation | ─ |
| **71** | female | Cardiomegaly, Cardiomyopathy, Right ventricular noncompaction cardiomyopathy, Hydrops fetalis | **TPM1(191010)** | NM_001018005.1 | c.122A>G** | p.(Asp41Gly) | missense | Het | LP | PS2, PM2, PP3 | De novo | AD | CARDIOMYOPATHY, DILATED, 1Y(611878) | Termination | Prospective | Cardiomyopathy | ─ |
| **72** | male | Cardiac rhabdomyoma | TSC2(191092) | NM_000548.4 | c.2742+2T>G** | - | splice donor | Het | LP | PVS1_PS, PS2_PM, PM2 | De novo | AD | TUBEROUS SCLEROSIS 2(613254) | Termination | Prospective | Cardiac rhabdomyoma | ─ |
| **73** | female | Cardiomegaly | FBN1(134797) | NM_000138.4 | c.5788+5G>A | - | splice region | Het | P | PS2_PVS, PS3_PM, PM2 | De novo | AD | MARFAN SYNDROME(154700) | Live birth | Prospective | Cardiomegaly | ─ |
| **74** | female | Coarctation of aorta,Ventricular septal defect | PTPN11(176876) | NM_002834.3 | c.922A>G | p.(Asn308Asp) | missense | Het | P | PP2, PP3, PM2, PP1_PS, PS2_PVS, PS3 | Mat(affected) | AD | NOONAN SYNDROME 1(163950) | Live birth | Prospective | Coarctation of aorta,Ventricular septal defect | ─ |
| **75** | female | Ventricular septal defect, Pulmonary artery stenosis | EFTUD2(603892) | NM_004247.3 | c.1064dupA** | p.(Phe356ValfsTer8) | frameshift | Het | P | PVS1,PS2_PM, PM2 | De novo | AD | MANDIBULOFACIAL DYSOSTOSIS, GUION-ALMEIDA TYPE(610536) | Termination | Prospective | Ventricular septal defect | ─ |
| **76** | female | Pulmonic stenosis, Persistent left superior vena cava | PTPN11(176876) | NM_002834.3 | c.854T>C | p.(Phe285Ser) | missense | Het | P | PS2_PVS, PS4_PM, PM2, PM5, PP3 | De novo | AD | NOONAN SYNDROME 1(163950) | Termination | Prospective | Pulmonic stenosis | ─ |
| **77** | female | Coarctation of aorta | KMT2D(602113) | NM_003482.3 | c.11695C>T** | p.(Gln3899Ter) | nonsense | Het | P | PVS1, PS2_PP, PM2 | De novo | AD | KABUKI SYNDROME 1(147920) | Termination | Prospective | Coarctation of aorta | ─ |
| **78** | male | Cardiac rhabdomyoma | TSC2(191092) | NM_000548.4 | c.3685C>T | p.(Gln1229Ter) | nonsense | Het | P | PVS1, PS2_PM, PS4_PP, PM2 | De novo | AD | TUBEROUS SCLEROSIS 2(613254) | Termination | Prospective | Cardiac rhabdomyoma | ─ |
| **79** | male | Coarctation of aorta, Anomalous origin of left subclavian artery | KMT2D(602113) | NM_003482.3 | c.1583delC** | p.(Pro528HisfsTer402) | frameshift | Het | P | PVS1, PS2_PP, PM2 | De novo | AD | KABUKI SYNDROME 1(147920) | Termination | Prospective | Coarctation of aorta | ─ |
| **80** | male | Hypoplasia of right ventricle | **MYH7(160760)** | NM_000257.3 | c.2779G>A | p.(Glu927Lys) | missense | Het | LP | PS2_PP, PS4_PM, PM1_PP, PM2, PP3 | De novo | AD | CARDIOMYOPATHY, FAMILIAL HYPERTROPHIC, 1(192600) | Live birth | Prospective | cardiovascular system | ─ |
| **81** | female | Increased nuchal translucency, Cystic hygroma, Hypoplastic left heart | LZTR1(600574) | NM_006767.3 | c.593+2T>C** | - | splice donor | Het | LP | PVS1, PM2, PM3 | Pat | AR | NOONAN SYNDROME 2(605275) | Termination | Prospective | Cystic hygroma | ─ |
|  |  |  |  | NM_006767.3 | c.1785+1G>A | - | splice donor | Het | LP | PVS1, PM2, PM3 | Mat |  |  |  |  |  |  |
| **82** | male | Complete atrioventricular canal defect,Coarctation of aorta,Echogenic fetal bowel | NR2F2(107773) | NM_021005.3 | c.265A>G** | p.(Lys89Glu) | missense | Het | LP | PS2_PM, PM2, PP3_PM | De novo | AD | CONGENITAL HEART DEFECTS, MULTIPLE TYPES, 4(615779) | In pregnancy | Prospective | Coarctation of aorta | ─ |
| **Abdominal** | | |  |  |  |  |  |  |  |  |  |  |  |  |  |  |  |
| **83 ^#^** | female | Pyloric stenosis, Gastrectasia | FOXF1(601089) | NM_001451.2 | c.231C>G** | p.(Phe77Leu) | missense | Het | LP | PS2_PM, PM1, PM2, PP3 | De novo | AD | ALVEOLAR CAPILLARY DYSPLASIA WITH MISALIGNMENT OF PULMONARY VEINS(265380) | Neonatal death | Retrospective | Gastrointestinal system | ─ |
| **84** | male | Omphalocele | CDKN1C(600856) | NM_000076.2 | c.694C>T | p.(Gln232Ter) | nonsense | Het | P | PVS1, PS2_PM, PM2 | De novo | AD | BECKWITH-WIEDEMANN SYNDROME; BWS(130650) | Termination | Prospective | Omphalocele | ─ |
| **Urogenital** | | |  |  |  |  |  |  |  |  |  |  |  |  |  |  |  |
| **85** | male | Multicystic kidney dysplasia | **UMOD(191845)** | NM_001278614.1 | c.1416dupA | p.(Gln473ThrfsTer43) | frameshift | Het | LP | PVS1, PS2_PM, PM2 | De novo | AD | TUBULOINTERSTITIAL KIDNEY DISEASE, AUTOSOMAL DOMINANT, 1(1162000) | Live birth | Retrospective | Urogenital system | ─ |
| **86** | female | Hyperechogenic kidneys, Multicystic kidney dysplasia | HNF1B(189907) | NM_000458.2 | c.188_191dupATAC | p.(Leu65TyrfsTer24) | frameshift | Het | P | PVS1, PS2_PM, PM2 | De novo | AD | RENAL CYSTS AND DIABETES SYNDROME(137920) | Live birth | Retrospective | Multicystic kidney dysplasia | ─ |
| **87** | female | Enlarged kidney, Hyperechogenic kidneys, Choroid plexus cyst | PKD1(601313) | NM_001009944.2 | c.6894G>C | p.(Trp2298Cys) | missense | Het | LP | PM1_PP, PM2, PP3, PP4 | Mat(affected) | AD | POLYCYSTIC KIDNEY DISEASE 1 WITH OR WITHOUT POLYCYSTIC LIVER DISEASE(173900) | Live birth | Retrospective | Enlarged kidney | ─ |
| **88** | male | Multicystic kidney dysplasia | JAG1(601920) | NM_000214.2 | c.205G>A | p.(Asp69Asn) | missense | Het | LP | PS2_PM, PM1_PP, PM2, PP3 | De novo | AD | ALAGILLE SYNDROME 1(118450) | Termination | Retrospective | Renal dysplasia | ─ |
| **89** | male | Enlarged kidney, Hyperechogenic kidneys,Oligohydramnios | ACE(106180) | NM_000789.3 | c.418-2A>G | - | splice acceptor | Het | P | PVS1, PM2, PM3 | Pat | AR | RENAL TUBULAR DYSGENESIS(267430) | Termination | Retrospective | Oligohydramnios | ─ |
|  |  |  |  | NM_000789.3 | c.1028G>A | p.(Trp343Ter) | nonsense | Het | P | PVS1, PM2, PM3 | Mat |  |  |  |  |  |  |
| **90** | male | Micropenis, Hypospadias | SRD5A2(607306) | NM_000348.3 | c.680G>A | p.(Gly227Glu) | missense | Het | P | PM3_PVS, PS3_PM, PP4 | Mat | AR | PSEUDOVAGINAL PERINEOSCROTAL HYPOSPADIAS(264600) | Live birth | Retrospective | Micropenis, Hypospadias | ─ |
|  |  |  |  | NM_000348.3 | c.16C>T | p.(Gln6Ter) | nonsense | Het | P | PVS1, PM2, PM3_PS | Pat |  |  |  |  |  |  |
| **91** | female | Enlarged kidney, Hyperechogenic kidneys | PKD2(173910) | NM_000297.3 | c.2020-4_2024delACAGAATAT | - | splice acceptor | Het | LP | PVS1_PM, PM2, PS2_PM | De novo | AD | POLYCYSTIC KIDNEY DISEASE 2 WITH OR WITHOUT POLYCYSTIC LIVER DISEASE(613095) | Live birth | Prospective | Enlarged kidney | ─ |
| **92** | male | Hydronephrosis | HNF1B(189907) | NM_000458.2 | c.716G>T** | p.(Gly239Val) | missense | Het | LP | PS2_PP, PM1_PP, PM2, PM5_PP, PP3_PM | De novo | AD | RENAL CYSTS AND DIABETES SYNDROME(137920) | Live birth | Prospective | Urogenital system | ─ |
| **93** | female | Hyperechogenic kidneys, Enlarged kidney | PKD2(173910) | NM_000297.3 | c.959G>A** | p.(Arg320Gln) | missense | Het | LP | PM1, PM2, PP3, PP4, PP1 | Mat(affected) | AD | POLYCYSTIC KIDNEY DISEASE 2 WITH OR WITHOUT POLYCYSTIC LIVER DISEASE(613095) | Termination | Prospective | Hyperechogenic kidneys, Enlarged kidney | ─ |
| **94** | female | Hyperechogenic kidneys, Renal cysts | HNF1B(189907) | NM_000458.2 | c.1397_1412delTGGCAGCCCTGCAGCC** | p.(Leu466ProfsTer34) | frameshift | Het | LP | PVS1, PM2 | Mat(asymptomatic) | AD | RENAL CYSTS AND DIABETES SYNDROME(137920) | Live birth | Prospective | Renal cysts | ─ |
| **95** | male | Multicystic kidney dysplasia | HNF1B(189907) | NM_000458.2 | c.544+1G>A | - | splice donor | Het | P | PVS1, PS4_PP, PM2 | Mat(affected) | AD | RENAL CYSTS AND DIABETES SYNDROME(137920) | Live birth | Prospective | Multicystic kidney dysplasia | ─ |
| **96** | female | Enlarged kidney, Hyperechogenic kidneys | PKHD1(606702) | NM_138694.3 | c.4742A>T** | p.(Lys1581Met) | missense | Het | LP | PM1, PM2, PM3, PP4 | Pat | AR | POLYCYSTIC KIDNEY DISEASE 4 WITH OR WITHOUT POLYCYSTIC LIVER DISEASE(263200) | Neonatal death | Prospective | Enlarged kidney | ─ |
|  |  |  |  | NM_138694.3 | c.2341C>T | p.(Arg781Ter) | nonsense | Het | P | PVS1, PM2, PM3, PP4 | Mat |  |  |  |  |  |  |
| **97** | female | Enlarged kidney, Hyperechogenic kidneys,Oligohydramnios | PKHD1(606702) | NM_138694.3 | c.5869G>A | p.(Asp1957Asn) | missense | Het | LP | PM1_PP, PM2, PM3, PP3, PP4 | Pat | AR | POLYCYSTIC KIDNEY DISEASE 4 WITH OR WITHOUT POLYCYSTIC LIVER DISEASE(263200) | Termination | Prospective | Enlarged kidney, Oligohydramnios | ─ |
|  |  |  |  | NM_138694.3 | c.107C>T | p.(Thr36Met) | missense | Het | P | PM2, PM3_PVS, PP1, PP3 | Mat |  |  |  |  |  |  |
| **98** | male | Enlarged kidney, Hyperechogenic kidneys | PKD1(601313) | NM_001009944.2 | c.11888G>A** | p.(Trp3963Ter) | nonsense | Het | LP | PVS1, PM2 | Pat(affected) | AD | POLYCYSTIC KIDNEY DISEASE 1 WITH OR WITHOUT POLYCYSTIC LIVER DISEASE(173900) | Live birth | Prospective | Enlarged kidney | ─ |
| **99** | male | Megacystis | ACTG2(102545) | NM_001615.3 | c.770G>A | p.(Arg257His) | missense | Het | P | PS2_PVS, PM2, PM5, PP3 | Mat(asymptomatic) | AD | VISCERAL MYOPATHY 1(155310) | Live birth | Prospective | Megacystis | ─ |
| **Skeletal** | | |  |  |  |  |  |  |  |  |  |  |  |  |  |  |  |
| **100** | female | Increased nuchal translucency, Short long bone, Bowing of the long bones, Narrow chest, Abnormal skull morphology | FGFR3(134934) | NM_000142.4 | c.742C>T | p.(Arg248Cys) | missense | Het | P | PS2, PS3_PM, PS4_PVS, PM2, PP3 | De novo | AD | THANATOPHORIC DYSPLASIA, TYPE I(187600) | Termination | Retrospective | Increased nuchal translucency, Short long bone, Bowing of the long bones, Narrow chest, Abnormal skull morphology | ─ |
| **101** | female | Abnormal skull morphology, Micromelia | FGFR3(134934) | NM_000142.5 | c.1118A>G | p.(Tyr373Cys) | missense | Het | P | PS2_PVS, PS4, PM2, PP3 | De novo | AD | THANATOPHORIC DYSPLASIA, TYPE I(187600) | Termination | Retrospective | Abnormal skull morphology, Micromelia | ─ |
| **102** | male | Short long bone,Wind-swept deformity of the knees | GDF5(601146)* | NM_000557.5 | c.1335T>G | p.(Asn445Lys) | missense | Het | P | PS2_PM, PM1_PP, PM2, PP3 | De novo | AD | MULTIPLE SYNOSTOSES SYNDROME 2(610017) | Termination | Retrospective | Short long bone | ─ |
| **103** | male | Decreased calvarial ossification,Multiple prenatal fractures | COL1A1(120150) | NM_000088.3 | c.2596G>A | p.(Gly866Ser) | missense | Het | P | PS2_PVS, PM1, PM2, PP3 | De novo | AD | OSTEOGENESIS IMPERFECTA, TYPE III(259420) | Termination | Retrospective | Decreased calvarial ossification,Multiple prenatal fractures | ─ |
| **104** | male | Short long bone | SHOX(312865) | NM_000451.3 | c.517C>T | p.(Arg173Cys) | missense | Het | P | PS2, PS4_PM, PM2, PM5 | De novo | AD | LERI-WEILL DYSCHONDROSTEOSIS(127300) | Termination | Retrospective | Short long bone | ─ |
| **105** | male | Talipes equinovarus | FLNB(603381) | NM_001457.4 | c.535G>A** | p.(Ala179Thr) | missense | Het | LP | PS2_PM, PM1_PP, PM2, PP3 | De novo | AD | ATELOSTEOGENESIS, TYPE III(108721) | Termination | Retrospective | Talipes equinovarus | ─ |
| **106** | female | Short long bone | FGFR3(134934) | NM_000142.5 | c.1620C>G | p.(Asn540Lys) | missense | Het | P | PS2, PS4, PM2 | De novo | AD | HYPOCHONDROPLASIA(146000) | Live birth | Retrospective | Short long bone | ─ |
| **107** | male | Redundant neck skin, Abnormal posturing, Flexion contracture | SCN4A(603967)* | NM_000334.4 | c.3502delC** | p.(Leu1168SerfsTer5) | frameshift | Het | LP | PVS1, PM2 | Mat | AR | MYASTHENIC SYNDROME, CONGENITAL, 16(614198) | Termination | Retrospective | Flexion contracture | ─ |
|  |  |  |  | NM_000334.4 | c.3395G>C** | p.(Arg1132Pro) | missense | Het | LP | PM2, PM3_PP, PM5, PP3 | De novo |  |  |  |  |  |  |
| **108** | male | Hypoplasia of the ulna, Radial dysplasia, Abnormality of digit | EZH2(601573)* | NM_004456.4 | c.47G>A** | p.(Arg16Gln) | missense | Het | LP | PS2_PM, PM1_PP, PM2, PP3 | De novo | AD | WEAVER SYNDROME(277590) | Termination | Retrospective | Abnormality of digit | Hypoplasia of the ulna, Radial dysplasia |
| **109** | female | Femoral bowing | COL1A2(120160) | NM_000089.3 | c.577G>A | p.(Gly193Ser) | missense | Het | P | PS2, PS4_PM, PM1, PM2, PP3 | De novo | AD | OSTEOGENESIS IMPERFECTA, TYPE IV(166220) | Live birth | Retrospective | Femoral bowing | ─ |
| **110** | female | Abnormality of the middle and the distal phalanx of the 3rd finger,Abnormality of the index finger,Split foot | PUF60(604819)* | NM_078480.2 | c.24+3A>T** | - | splice region | Het | LP | PVS1_PM, PS2_PM, PM2 | De novo | AD | VERHEIJ SYNDROME(615583) | Termination | Retrospective | Skeletal system | Split foot |
| **111** | female | Short long bone | FGFR3(134934) | NM_000142.5 | c.1138G>A | p.(Gly380Arg) | missense | Het | P | PS2_PVS, PS4_PVS, PM2 | De novo | AD | ACHONDROPLASIA(100800) | Live birth | Retrospective | Short long bone | ─ |
| **112** | female | Short long bone | COL2A1(120140) | NM_001844.4 | c.1365+1G>A** | - | splice donor | Het | P | PVS1, PS2_PM, PM2 | De novo | AD | ACHONDROGENESIS, TYPE II(200610) | Termination | Retrospective | Short long bone | ─ |
| **113** | male | Short long bone | GNPTAB(607840) | NM_024312.4 | c.1284+1G>T | - | splice donor | Het | LP | PVS1_PM, PM2, PM3_PS | Pat | AR | MUCOLIPIDOSIS III ALPHA/BETA (252600) | Live birth | Retrospective | Short long bone | ─ |
|  |  |  |  | NM_024312.4 | c.1090C>T | p.(Arg364Ter) | nonsense | Het | P | PVS1,PM2, PM3 | Mat |  |  |  |  |  |  |
| **114** | female | Talipes equinovarus, Abnormality of the hand | ZC4H2(300897)* | NM_018684.3 | c.562-1G>T** | - | splice acceptor | Het | P | PVS1_PS, PS2 | De novo | XL | WIEACKER-WOLFF SYNDROME, FEMALE-RESTRICTED(301041) | Termination | Retrospective | Talipes equinovarus, Abnormality of the hand | ─ |
| **115** | male | Hand clenching, Clinodactyly of hallux, Talipes equinovarus | TNNI2(191043) | NM_003282.3 | c.499_501delGAG | p.(Glu167del) | In-frame | Het | LP | PS4_PM, PM2, PM4 | Pat(affected) | AD | ARTHROGRYPOSIS, DISTAL, TYPE 2B1(601680) | Live birth | Retrospective | Talipes equinovarus | ─ |
| **116** | female | Short long bone | SMAD4(600993)* | NM_005359.5 | c.1498A>G | p.(Ile500Val) | missense | Het | P | PS2, PS4, PM2_PP, PM5, PP3 | De novo | AD | MYHRE SYNDROME(139210) | Live birth | Retrospective | Short long bone | ─ |
| **117** | female | Short long bone | EBP(300205) | NM_006579.2 | c.187C>T | p.(Arg63Ter) | nonsense | Het | P | PVS1, PS2_PM, PS4_PP, PM2 | De novo | XD | CHONDRODYSPLASIA PUNCTATA 2,X-LINKED DOMINANT(302960) | Live birth | Retrospective | Skeletal system | ─ |
| **118** | female | Talipes equinovarus, Hand clenching | TPM2(190990)* | NM_003289.4 | c.463G>A | p.(Ala155Thr) | missense | Het | LP | PS3_PP, PS4_PM, PM2, PP3 | Mat(affected) | AD | ARTHROGRYPOSIS, DISTAL, TYPE 1A(108120) | Live birth | Retrospective | Talipes equinovarus, Hand clenching | ─ |
| **119** | male | Camptodactyly, FGR | KMT2D(602113) | NM_003482.3 | c.11041C>T** | p.(Gln3681Ter) | nonsense | Het | P | PVS1, PS2_PP, PM2 | De novo | AD | KABUKI SYNDROME 1(147920) | Termination | Retrospective | Skeletal system | ─ |
| **120** | female | Arthrogryposis multiplex congenita | ASXL3(615115) | NM_030632.2 | c.3039+1G>T | - | splice donor | Het | P | PVS1, PS2_PM, PM2 | De novo | AD | BAINBRIDGE-ROPERS SYNDROM(615485) | Live birth | Retrospective | skeletal system | ─ |
| **121** | female | Short femur | TRPV4(605427) | NM_021625.4 | c.2353T>C** | p.(Trp785Arg) | missense | Het | LP | PS2, PM2, PP3 | De novo | AD | PARASTREMMATIC DWARFISM(168400) | Live birth | Retrospective | skeletal system | ─ |
| **122** | male | Talipes equinovarus | COL6A1(120220) | NM_001848.2 | c.525delT** | p.(Val176Ter) | frameshift | Het | LP | PVS1, PM2 | Mat | AR | ULLRICH CONGENITAL MUSCULAR DYSTROPHY 1(254090);BETHLEM MYOPATHY 1(158810) | Termination | Retrospective | Talipes equinovarus | ─ |
|  |  |  |  | NM_001848.2 | c.1057-2A>G** | - | splice acceptor | Het | P | PVS1, PM2, PS2_Supporting | De novo |  |  |  |  |  |  |
| **123** | male | Short femur, femur bowing | COL1A1(120150) | NM_000088.3 | c.2569G>T | p.(Gly857Cys) | missense | Het | P | PS2, PM, PM2, PP3, PP2 | De novo | AD | OSTEOGENESIS IMPERFECTA, TYPE IV(166220) | Termination | Retrospective | Femur bowing | ─ |
| **124** | female | Macrocephaly, Micromelia | FGFR3(134934) | NM_000142.5 | c.1138G>A | p.(Gly380Arg) | missense | Het | P | PS2, PM2, PP3, PS3, PS1 | De novo | AD | ACHONDROPLASIA(100800) | Termination | Retrospective | Macrocephaly, Micromelia | ─ |
| **125** | male | Short long bone, Bowed humerus, Femoral bowing | COL1A2(120160) | NM_000089.3 | c.2405G>A** | p.(Gly802Asp) | missense | Het | LP | PS2, PM1, PM2, PP3 | De novo | AD | OSTEOGENESIS IMPERFECTA, TYPE II(166210) | Termination | Retrospective | Femoral bowing | ─ |
| **126** | male | Hand polydactyly, decreased fetal movement, FGR | ATRX(300032) | NM_000489 | 1035 bp deletion** | - | structural variant | Hemi | LP | PVS1, PM2 | Mat | XR | MENTAL RETARDATION-HYPOTONIC FACIES SYNDROME, X-LINKED, 1(309580) | Live birth | Retrospective | Skeletal system | ─ |
| **127** | female | Micromelia, Increased nuchal translucency | FGFR3(134934) | NM_000142.5 | c.742C>T | p.(Arg248Cys) | missense | Het | P | PM2, PP3, PS2_PVS, PS3, PM1 | De novo | AD | THANATOPHORIC DYSPLASIA, TYPE I(187600) | Termination | Prospective | Micromelia, Increased nuchal translucency | ─ |
| **128** | male | Short long bone | COL2A1(120140) | NM_001844.4 | c.872G>T** | p.(Gly291Val) | missense | Het | LP | PS2, PM2, PP3_PM | De novo | AD | ACHONDROGENESIS, TYPE II(200610) | Termination | Prospective | Short long bone | ─ |
| **129** | male | Short long bone | FGFR3(134934) | NM_000142.5 | c.1138G>A | p.(Gly380Arg) | missense | Het | P | PS2, PM2, PP3, PS3, PS1 | De novo | AD | ACHONDROPLASIA(100800) | Termination | Prospective | Short long bone | ─ |
| **130** | female | Short long bone, Femoral bowing | COL1A1(120150) | NM_000088.3 | c.3118G>A | p.(Gly1040Ser) | missense | Het | P | PM1, PM2, PP3, PS4_PM, PS2_PVS | De novo | AD | OSTEOGENESIS IMPERFECTA, TYPE IV(166220) | Termination | Prospective | Femoral bowing | ─ |
| **131** | male | Short long bone, Increased head circumference, Polyhydramnios | FGFR3(134934) | NM_000142.5 | c.1138G>A | p.(Gly380Arg) | missense | Het | P | PS2, PM2, PP3, PS3, PS1 | De novo | AD | ACHONDROPLASIA(100800) | Termination | Prospective | Short long bone, Increased head circumference, Polyhydramnios | ─ |
| **132** | male | Short long bone, Femoral bowing, Bowed humerus | FGFR3(134934) | NM_000142.5 | c.1138G>A | p.(Gly380Arg) | missense | Het | P | PS2, PM2, PP3, PS3, PS1 | De novo | AD | ACHONDROPLASIA(100800) | Termination | Prospective | Short long bone, Femoral bowing | ─ |
| **133** | female | Short long bone, Femoral bowing, Echogenic fetal bowel | COL2A1(120140) | NM_001844.4 | c.1654G>A | p.(Gly552Arg) | missense | Het | LP | PS2, PM2, PP3, PP2 | De novo | AD | SPONDYLOEPIPHYSEAL DYSPLASIA CONGENITA(183900) | Termination | Prospective | Short long bone, Femoral bowing | ─ |
| **134** | male | Femur fracture | COL1A1(120150) | NM_000088.3 | c.1751G>A** | p.(Gly584Glu) | missense | Het | P | PS2, PM1, PM2, PP3_PM | De novo | AD | OSTEOGENESIS IMPERFECTA, TYPE II(166210) | Termination | Prospective | Multiple prenatal fractures | ─ |
| **135** | female | Multiple prenatal fractures | COL1A1(120150) | NM_000088.3 | c.4249-3_4249-2delCA | - | splice acceptor | Het | LP | PS2, PM2, PVS1_PM, PS4_PP | De novo | AD | OSTEOGENESIS IMPERFECTA, TYPE IV(166220) | Termination | Prospective | Multiple prenatal fractures | ─ |
| **136** | male | Short long bone | COL2A1(120140) | NM_001844.4 | c.1475G>A | p.(Gly492Asp) | missense | Het | LP | PS2, PM2, PP3, PM1, PP2 | De novo | AD | SPONDYLOEPIPHYSEAL DYSPLASIA CONGENITA(183900) | Termination | Prospective | Short long bone | ─ |
| **137** | female | Short femur, Femoral bowing | COL1A2(120160) | NM_000089 | c.3305G>A | p.(G1102D) | missense | Het | LP | PS2, PM1, PP3, PM2 | De novo | AD | OSTEOGENESIS IMPERFECTA, TYPE IV(166220) | Termination | Prospective | Femoral bowing | ─ |
| **138** | male | Short long bone, Femoral bowing | COL1A1(120150) | NM_000088.3 | c.2362G>A | p.(Gly788Ser) | missense | Het | P | PM1, PM2, PP3, PP2 | Pat(asymptomatic) | AD | OSTEOGENESIS IMPERFECTA, TYPE I(166200) | Termination | Prospective | Short long bone, Femoral bowing | ─ |
| **139** | male | Short long bone, Femoral bowing | COL1A1(120150) | NM_000088.3 | c.1678G>A | p.(Gly560Ser) | missense | Het | P | PM1, PM2, PP3, PP2, PP1, PM5, PS4 | Mat(affected) | AD | OSTEOGENESIS IMPERFECTA, TYPE IV(166220) | Termination | Prospective | Short long bone, Femoral bowing | ─ |
| **140** | female | Short long bone, Bowing of the long bones | FGFR3(134934) | NM_000142.5 | c.1949A>T | p.(Lys650Met) | missense | Het | P | PM2, PP3, PS3, PS2_PVS | De novo | AD | THANATOPHORIC DYSPLASIA, TYPE I(187600) | Termination | Prospective | Short long bone, Bowing of the long bones | ─ |
| **141** | female | Short long bone, Scoliosis | OBSL1(610991) | NM_015311.2 | c.1283-1G>A** | - | splice acceptor | Het | LP | PVS1,PM2 | Mat | AR | THREE M SYNDROME 2(612921) | Termination | Prospective | Scoliosis | ─ |
|  |  |  |  | NM_015311.2 | c.409C>T** | p.(Gln137Ter) | nonsense | Het | LP | PVS1, PM2 | Pat |  |  |  |  |  |  |
| **142** | female | Polyhydramnios, Abnormality of the vertebral column, Femoral bowing, Bowed humerus, | ALPL(171760) | NM_000478.5 | c.18delA | p.(Val7TyrfsTer12) | frameshift | Het | P | PVS1, PM2, PM3 | Pat | AR | HYPOPHOSPHATASIA, INFANTILE(241500) | Termination | Prospective | Polyhydramnios, Abnormality of the vertebral column | ─ |
|  |  |  |  | NM_000478.5 | c.346G>A | p.(Ala116Thr) | missense | Het | P | PS3_PP, PM1, PM2, PM3, PP3_PM | Mat |  |  |  |  |  |  |
| **143** | female | Hand oligodactyly, Toe syndactyly | TP63(603273) | NM_003722.4 | c.946A>G** | p.(Met316Val) | missense | Het | LP | PM1, PM2, PP3, PP1 | Mat(affected) | AD | SPLIT-HAND/FOOT MALFORMATION 4(605289) | Termination | Prospective | Toe syndactyly, Oligodactyly | ─ |
| **144** | female | Femoral bowing, Short femur | COL1A1(120150) | NM_000088.3 | c.1084G>T | p.(Gly362Cys) | missense | Het | P | PS2, PM1, PM2, PP3, PP2 | De novo | AD | OSTEOGENESIS IMPERFECTA, TYPE IV(166220) | Termination | Prospective | Femoral bowing | ─ |
| **145** | male | Talipes equinovarus, Long bone bowing | COL1A1(120150) | NM_000088.3 | c.3407G>C | p.(Gly1136Ala) | missense | Het | P | PS2, PM1, PM2, PP3, PP2 | De novo | AD | OSTEOGENESIS IMPERFECTA, TYPE II(166210) | Termination | Prospective | Long bone bowing | ─ |
| **146** | female | Polyhydramnios, Short long bone, Macrocephaly | FGFR3(134934) | NM_000142.5 | c.1138G>A | p.(Gly380Arg) | missense | Het | P | PS2, PM2, PP3, PS3, PS1 | De novo | AD | ACHONDROPLASIA(100800) | Termination | Prospective | Polyhydramnios, Short long bone, Macrocephaly | ─ |
| **147** | female | Short long bone, Hypoplasia of the ulna, Fibular hypoplasia, Thoracic hypoplasia | DYNC2H1(603297) | NM_001080463.1 | c.5984C>T | p.(Ala1995Val) | missense | Homo | LP | PM1, PM2, PM5 | Mat+Pat | AR | SHORT-RIB THORACIC DYSPLASIA 3 WITH OR WITHOUT POLYDACTYLY(613091) | Termination | Prospective | Short long bone, Hypoplasia of the ulna, Fibular hypoplasia, Thoracic hypoplasia | ─ |
| **148** | male | Increased nuchal translucency, Macrocephaly, Narrow chest | FGFR3(134934) | NM_000142.5 | c.1948A>G | p.(Lys650Glu) | missense | Het | P | PS2, PS3, PM1, PM2, PM5, PP3 | De novo | AD | THANATOPHORIC DYSPLASIA, TYPE II(187601) | Termination | Prospective | Increased nuchal translucency, Macrocephaly, Narrow chest | ─ |
| **149** | male | Increased nuchal translucency, Micromelia | COL2A1(120140) | NM_001844.4 | c.2717G>A** | p.(Gly906Glu) | missense | Het | LP | PS2, PM2, PP3, PP2 | De novo | AD | ACHONDROGENESIS, TYPE II(200610) | Termination | Prospective | Micromelia | ─ |
| **150** | male | Short long bone, Bowing of the long bones | COL1A1(120150) | NM_000088.3 | c.3470G>T | p.(Gly1157Val) | missense | Het | LP | PM1, PM2, PP3, PP2 | Mat(affected) | AD | OSTEOGENESIS IMPERFECTA, TYPE IV(166220) | Lost to follow up | Prospective | Bowing of the long bones | ─ |
| **151** | male | Short long bone | COL1A1(120150) | NM_000088.3 | c.986G>T | p.(Gly329Val) | missense | Het | LP | PM1, PM2, PM5, PP3, PP2 | Mat(affected) | AD | OSTEOGENESIS IMPERFECTA, TYPE IV(166220) | Termination | Prospective | Short long bone | ─ |
| **152** | male | Contractures of elbows, Extension contractures of knees | ECEL1(605896) | NM_004826.3 | c.110_155del46 | p.(Phe37CysfsTer151) | frameshift | Het | P | PVS1, PM2, PP4 | Mat | AR | ARTHROGRYPOSIS, DISTAL, TYPE 5D; DA5D(615065) | Termination | Prospective | Contractures of elbows, Extension contractures of knees | ─ |
|  |  |  |  | NM_004826.3 | c.633G>C** | p.(Trp211Cys) | missense | Het | LP | PM2, PM3, PP3, PP4 | Pat |  |  |  |  |  |  |
| **153** | male | Depressed nasal bridge, Epiphyseal stippling | ARSE(300180) | NM_000047.3 | c.331C>TT | p.(Arg111Cys) | missense | Hemi | LP | PM1, PM2, PP3, PP1, PP4 | Mat | XR | CHONDRODYSPLASIA PUNCTATA 1, X-LINKED RECESSIVE(302950) | Live birth | Prospective | Depressed nasal bridge, Epiphyseal stippling | ─ |
| **154** | male | Overlapping fingers, Talipes equinovarus | KLHL40(615340) | NM_152393.3 | c.602G>A | p.(Trp201Ter) | nonsense | Het | P | PVS1, PM2, PM3_PP | Pat | AR | NEMALINE MYOPATHY 8(615348) | Termination | Prospective | Abnormality of finger | ─ |
|  |  |  |  | NM_152393.3 | c.1516A>C | p.(Thr506Pro) | missense | Het | P | PM3_PVS, PM2, PP3 | Mat |  |  |  |  |  |  |
| **155** | female | Short femur, Increased head circumference | FGFR3(134934) | NM_000142.5 | c.1138G>A | p.(Gly380Arg) | missense | Het | P | PS2, PM2, PP3, PS3, PS1 | De novo | AD | ACHONDROPLASIA(100800) | Termination | Prospective | Short femur, Increased head circumference | ─ |
| **156** | male | Short long bone, Macrocephaly | FGFR3(134934) | NM_000142.5 | c.1138G>A | p.(Gly380Arg) | missense | Het | P | PS2, PM2, PP3, PS3, PS1 | De novo | AD | ACHONDROPLASIA(100800) | Termination | Prospective | Short long bone, Macrocephaly | ─ |
| **157** | male | Short long bone | COL1A2(120160) | NM_000089.3 | c.1684G>A** | p.(Gly562Ser) | missense | Het | LP | PM1, PM2, PP3, PP4 | Mat(affected) | AD | OSTEOGENESIS IMPERFECTA, TYPE II(166210); OSTEOGENESIS IMPERFECTA, TYPE IV(166220); OSTEOGENESIS IMPERFECTA, TYPE III(259420) | Termination | Prospective | Short long bone | ─ |
| **158** | female | Increased nuchal translucency, Micrognathia, Talipes equinovarus, Short femur | COL2A1(120140) | NM_001844.4 | c.2213G>T | p.(Gly738Val) | missense | Het | P | PS2, PM1, PM2, PP3, PP2 | De novo | AD | SPONDYLOEPIPHYSEAL DYSPLASIA CONGENITA(183900) | Termination | Prospective | Micrognathia, Talipes equinovarus, Short femur | ─ |
| **159** | female | Talipes equinovarus | BRPF1(602410)* | NM_001003694.1 | c.1723-1G>C** | - | splice acceptor | Het | P | PS2, PM2, PVS1_PS | De novo | AD | INTELLECTUAL DEVELOPMENTAL DISORDER WITH DYSMORPHIC FACIES AND PTOSIS (617333) | Termination | Prospective | Talipes equinovarus | ─ |
| **160** | female | Short long bone, Long bone bowing | COL1A1(120150) | NM_000088.3 | c.3854A>G** | p.(Asp1285Gly) | missense | Het | LP | PM1, PM2, PP3, PP2 | Mat(affected) | AD | OSTEOGENESIS IMPERFECTA, TYPE I(166200);OSTEOGENESIS IMPERFECTA, TYPE II(166210) | Live birth | Prospective | Short long bone, Long bone bowing | ─ |
| **161** | male | Polydactyly | EVC(604831) | NM_153717.2 | c.101T>C** | p.(Leu34Pro) | missense | Het | LP | PM2,P M3, PP1, PP4 | Mat | AR | ELLIS-VAN CREVELD SYNDROME(225500) | Termination | Prospective | Polydactyly | ─ |
|  |  |  |  | NM_153717.2 | c.2014C>T | p.(Gln672Ter) | nonsense | Het | P | PVS1, PM2, PP1 | Pat |  |  |  |  |  |  |
| **162** | female | Short long bone, Bowing of the long bones | SHOX(312865) | NM_000451 | 13.5 kb deletion (X1)** | - | structural variant | Het | P | PVS1, PM2, PP1 | Mat(affected) | AD | LERI-WEILL DYSCHONDROSTEOSIS(127300) | Live birth | Prospective | Bowing of the long bones | ─ |
| **FGR** |  |  |  |  |  |  |  |  |  |  |  |  |  |  |  |  |  |
| **163** | male | Intrauterine growth retardation | KAT6A(601408)* | NM_006766.4 | c.751C>T** | p.(Arg251Ter) | nonsense | Het | P | PVS1, PS2, PM2 | De novo | AD | ARBOLEDA-THAM SYNDROME(616268) | Live birth | Retrospective | Intrauterine growth retardation | ─ |
| **164** | female | Intrauterine growth retardation | NIPBL(608667) | NM_133433.3 | c.7789delC | p.(Leu2597CysfsTer14) | frameshift | Het | P | PVS1, PS2, PM2 | De novo | AD | CORNELIA DE LANGE SYNDROME 1(122470) | Termination | Prospective | Intrauterine growth retardation | ─ |
| **Isolated Hydrops** | | |  |  |  |  |  |  |  |  |  |  |  |  |  |  |  |
| **165** | female | Pleural effusion, ascites, Hydrops fetalis | PTPN11(176876) | NM_002834.3 | c.1507G>A | p.(Gly503Arg) | missense | Het | P | PS2, PM1, PM2, PP3, PS4, PS1, PP2 | De novo | AD | NOONAN SYNDROME 1(163950) | Termination | Prospective | PMID: 32144894, 18759865, 33686258 | ─ |
| **166** | male | Hydrops fetalis | RYR1(180901) | NM_000540.2 | c.844C>T | p.(Arg282Trp) | missense | Het | LP | PM3, PM2, PP3, PP4 | Mat | AR | MINICORE MYOPATHY WITH EXTERNAL OPHTHALMOPLEGIA(255320) | Termination | Prospective | Hydrops fetalis | ─ |
|  |  |  |  | NM_000540.2 | c.6158delC** | p.(Pro2053GlnfsTer12) | frameshift | Het | P | PVS1, PM2, PP4 | Pat |  |  |  |  |  |  |
| **Increased NT and cystic hygroma** | | | |  |  |  |  |  |  |  |  |  |  |  |  |  |  |
| **167** | male | Increased nuchal translucency | SETD5(615743) | NM_001080517.2 | c.2476+1G>A | - | splice donor | Het | P | PVS1, PS2, PM2 | De novo | AD | MENTAL RETARDATION, AUTOSOMAL DOMINANT 23(615761) | Live birth | Retrospective | Increased nuchal translucency | ─ |
| **168** | male | Increased nuchal translucency, cystic hygroma | PTPN11(176876) | NM_002834.3 | c.854T>C | p.(Phe285Ser) | missense | Het | P | PS2, PM1, PM2, PP3, PS4, PS3_PP, PP2 | De novo | AD | NOONAN SYNDROME 1(163950) | Termination | Retrospective | Cystic hygroma | ─ |
| **169** | male | Increased nuchal translucency | KMT2C(606833)* | NM_170606.2 | c.12906delT** | p.(Ala4303ProfsTer23) | frameshift | Het | P | PVS1, PS2, PM2 | De novo | AD | KLEEFSTRA SYNDROME 2(261652) | Termination | Prospective | Not reported previously. Homologous genes KMT2A/KMT2D HPO entry: Increased nuchal translucency | Increased nuchal translucency |
| **170** | male | Increased nuchal translucency, Bilateral choroid plexus cysts | KCNT1(608167)* | NM_020822.2 | c.1420C>T | p.(Arg474Cys) | missense | Het | P | PM2, PP3, PS2_PVS | De novo | AD | DEVELOPMENTAL AND EPILEPTIC ENCEPHALOPATHY 14(614959) | Termination | Prospective | Not reported previously | Increased nuchal translucency, Bilateral choroid plexus cysts |
| **171** | male | Increased nuchal translucency, cystic hygroma | HRAS(190020) | NM_005343.3 | c.38G>A | p.(Gly13Asp) | missense | Het | P | PS2, PM1, PM2, PP3, PM5_PS | De novo | AD | COSTELLO SYNDROME(218040) | Termination | Prospective | HPO entry: Redundant neck skin | ─ |
| **172** | female | Increased nuchal translucency, cystic hygroma | NFIB(600728)* | NM_001190737.1 | c.376A>G | p.(Lys126Glu) | missense | Het | LP | PM1, PP3, PS3_PP, PS2_PP, PM2 | De novo | AD | MACROCEPHALY, ACQUIRED, WITH IMPAIRED INTELLECTUAL DEVELOPMENT(618286) | Termination | Prospective | Not reported previously | Increased nuchal translucency, cystic hygroma |
| **Multisystem** | | |  |  |  |  |  |  |  |  |  |  |  |  |  |  |  |
| **173** | male | Increased nuchal translucency, Ventriculomegaly，Abnormal posturing of hand, Micropenis | RAF1(164760) | NM_002880.3 | c.770C>T | p.(Ser257Leu) | missense | Het | P | PP2, PM1, PM2, PS3, PS2 | De novo | AD | NOONAN SYNDROME 5(611553) | Neonatal death | Retrospective | Urogenital and skeletal systems; PMID: 23321623, 33568805, PMID: 23877478 | ─ |
| **174** | male | Spina bifida, Hemivertebrae | FLNB(603381) | NM_001164317.1 | c.7213C>A** | p.(Arg2405Ser) | missense | Het | LP | PS2, PM2, PP3 | De novo | AD | LARSEN SYNDROME(150250) | Termination | Retrospective | Spina bifida | ─ |
| **175** | male | Omphalocele, Abnormal foot morphology ,Short long bone, Narrow chest, Fibular aplasia | FLNA(300017) | NM_001110556.1 | c.583G>A** | p.(Gly195Ser) | missense | Hemi | LP | PM1, PM2, PP3_PS, PP2, PP4 | Mat | XL | MELNICK-NEEDLES SYNDROME(309350);OTOPALATODIGITAL SYNDROME, TYPE II(304120) | Termination | Retrospective | Omphalocele, Abnormal foot morphology ,Short long bone, Narrow chest, Fibular aplasia | ─ |
| **176** | male | Hydrocephalus, Talipes equinovarus | L1CAM(308840) | NM_000425.4 | c.3166+1G>A | - | splice donor | Hemi | LP | PM2, PP4, PVS1_PM, PP1 | Mat | XR | MASA SYNDROME(303350) | Termination | Retrospective | Hydrocephalus, Talipes equinovarus | ─ |
| **177** | female | Esophageal atresia, Right aortic arch | EFTUD2(603892) | NM_004247.3 | c.1705C>T | p.(Arg569Ter) | nonsense | Het | P | PVS1, PS2, PM2 | De novo | AD | MANDIBULOFACIAL DYSOSTOSIS, GUION-ALMEIDA TYPE(610536) | Termination | Retrospective | Esophageal atresia | ─ |
| **178** | female | Renal agenesis, Pulmonary hypoplasia, Left aortic arch with retroesophageal right subclavian artery, Pericardial effusion, Oligohydramnios | KMT2D(602113) | NM_003482.3 | c.10756_10758delAAG | p.(Lys3586del) | In-frame | Het | LP | PS2, PM2, PM4 | De novo | AD | KABUKI SYNDROME 1(147920) | Termination | Retrospective | Urogenital, cardiovascular and respiratory systems | ─ |
| **179** | female | Diaphragmatic hernia, Aplasia of the cerebellar vermis, Enlarged posterior fossa | SPECC1L(614140) | NM_015330.4 | c.1247C>T | p.(Ala416Val) | missense | Het | LP | PS2, PM2, PP3 | De novo | AD | OPITZ GBBB SYNDROME, TYPE II(145410) | Termination | Retrospective | Aplasia of the cerebellar vermis,Diaphragmatic hernia | ─ |
| **180** | male | Hydrocephalus, Ventricular septal defect | MRPS22(605810) | NM_020191.2 | c.732+1G>A | - | splice donor | Het | LP | PVS1, PM2 | Mat | AR | COMBINED OXIDATIVE PHOSPHORYLATION DEFICIENCY 5(611719) | Termination | Retrospective | central nervous and cardiovascular systems | ─ |
|  |  |  |  | NM_020191.2 | c.1009C>T | p.(Gln337Ter) | nonsense | Het | LP | PVS1, PM2 | Pat |  |  |  |  |  |  |
| **181** | male | Dandy-Walker malformation, Cleft palate, Cleft lip | MID1(300552) | NM_033290.3 | c.1222G>T | p.(Glu408Ter) | nonsense | Hemi | LP | PVS1, PM2 | Mat | XR | OPITZ GBBB SYNDROME, TYPE I(300000) | Termination | Retrospective | Cleft palate, Cleft lip | ─ |
| **182** | female | Interrupted aortic arch, Esophageal atresia, Persistent left superior vena cava | CHD7(608892) | NM_017780.3 | c.7252C>T | p.(Arg2418Ter) | nonsense | Het | P | PVS1, PS2, PM2 | De novo | AD | CHARGE SYNDROME(214800) | Termination | Retrospective | Interrupted aortic arch, Esophageal atresia | ─ |
| **183** | male | Cardiomegaly, Pulmonary artery stenosis, Ascites, Pericardial effusion | **MYH7(160760)** | NM_000257.3 | c.2135G>A | p.(Arg712His) | missense | Het | LP | PM1, PM2, PP3, PS2 | De novo | AD | CARDIOMYOPATHY, DILATED, 1S(613426) | Termination | Retrospective | Interrupted aortic arch, Tricuspid regurgitation, Cardiomegaly | ─ |
| **184** | male | Transposition of the great arteries, Pulmonary hypoplasia, Abdominal cystic mass, Enlarged kidney, Oligohydramnios | NEK8(609799) | NM_178170.2 | c.1418-1G>A | - | splice acceptor | Homo | P | PVS1, PM2, PP4 | Pat+Mat | AR | RENAL-HEPATIC-PANCREATIC DYSPLASIA 2(615415) | Termination | Retrospective | Pulmonary hypoplasia, Enlarged kidney, Oligohydramnios | ─ |
| **185** | female | Agenesis of the corpus callosum, Overlapping fingers, Overlapping toe, Hand clenching | FGFR2(176943) | NM_022970.3 | c.755C>G | p.(Ser252Trp) | missense | Het | P | PM2, PP3, PS4, PS2, PS3_PM | De novo | AD | APERT SYNDROME(101200) | Termination | Retrospective | Agenesis of the corpus callosum | ─ |
| **186** | male | Multicystic kidney dysplasia, Polydactyly | BBS7(607590) | NM_176824.2 | c.1611delA | p.(Gly538GlufsTer4) | frameshift | Het | LP | PVS1, PM2 | Mat | AR | BARDET-BIEDL SYNDROME 7(615984) | Termination | Retrospective | Multicystic kidney dysplasia, Polydactyly | ─ |
|  |  |  |  | NM_176824.2 | c.1002delT | p.(Asn335IlefsTer47) | frameshift | Het | LP | PVS1, PM2 | Pat |  |  |  |  |  |  |
| **187** | male | Renal agenesis, Pulmonary hypoplasia, Oligohydramnion, Ascites | FRAS1(607830) | NM_025074.6 | c.3563+1G>A | - | splice donor | Het | LP | PVS1, PM2 | Mat | AR | FRASER SYNDROME 1(219000) | Termination | Retrospective | Renal agenesis, Pulmonary hypoplasia | ─ |
|  |  |  |  | NM_025074.6 | c.10597C>T | p.(Arg3533Ter) | nonsense | Het | LP | PM2, PVS1 | Pat |  |  |  |  |  |  |
| **188** | female | Micrognathia, Ventricular septal defect | FGFR2(176943) | NM_022970.3 | c.833G>T | p.(Cys278Phe) | missense | Het | P | PP3_PM, PM2, PS4, PS2_PVS, PP1_PM | De novo | AD | APERT SYNDROME(101200) | Live birth | Retrospective | Micrognathia, Ventricular septal defect | ─ |
| **189** | male | Micrognathia, Arthrogryposis multiplex congenita, FGR | UBA1(314370)* | NM_153280.2 | c.1617G>T | p.(Met539Ile) | missense | Hemi | LP | PM1, PM2, PP3, PP4 | Mat | XR | SPINAL MUSCULAR ATROPHY, X-LINKED 2(301830) | Live birth | Retrospective | Micrognathia, Arthrogryposis multiplex congenita | ─ |
| **190** | female | Ventricular septal defect, Cleft palate | KMT2D(602113) | NM_003482.3 | c.15604delG** | p.(Ala5202ProfsTer41) | frameshift | Het | P | PVS1, PS2_PP, PM2 | De novo | AD | KABUKI SYNDROME 1(147920) | Live birth | Retrospective | Ventricular septal defect, Cleft palate | ─ |
| **191** | female | Short long bone, Coarctation of aorta,Intrauterine growth retardation | NIPBL(608667) | NM_133433.3 | c.7012G>C | p.(Ala2338Pro) | missense | Het | LP | PS2, PM2, PP3 | De novo | AD | CORNELIA DE LANGE SYNDROME 1(122470) | Termination | Retrospective | Intrauterine growth retardation; cardiovascular and skeletal systems | ─ |
| **192** | male | Coarctation of aorta, Cleft palate | KMT2D(602113) | NM_003482.3 | c.4223G>A | p.(Cys1408Tyr) | missense | Het | LP | PM2, PP3, PS2_PP, PM5 | De novo | AD | KABUKI SYNDROME 1(147920) | Termination | Retrospective | Coarctation of aorta, Cleft palate | ─ |
| **193** | male | Cleft palate, Hypospadias, Micropenis | NIPBL(608667) | NM_133433.3 | c.3855+5G>C** | - | splice region | Het | LP | PM2, PVS1_PS, PS2_PP | De novo | AD | CORNELIA DE LANGE SYNDROME 1(122470) | Termination | Retrospective | Cleft palate, Hypospadias | ─ |
| **194** | female | Intraventricular arachnoid cyst, Ventricular septal defect, Ventriculomegaly, Talipes equinovarus | USP9X(300072) | NM_001039590.2 | c.4607delG** | p.(Cys1536LeufsTer24) | frameshift | Het | P | PM2, PVS1, PS2_PP | De novo | XD | MENTAL RETARDATION, X-LINKED 99, SYNDROMIC, FEMALE-RESTRICTED(300968) | Termination | Retrospective | Ventriculomegaly | Intraventricular arachnoid cyst |
| **195** | male | Syndactyly, Cleft palate, Turricephaly | FGFR2(176943) | NM_022970.3 | c.755C>G | p.(Ser252Trp) | missense | Het | P | PM2_PP, PP3, PS4, PS2, PS3_PM | De novo | AD | APERT SYNDROME(101200) | Termination | Retrospective | Syndactyly, Cleft palate, Turricephaly | ─ |
| **196** | female | Ventricular septal defect, Coarctation of aorta, Ectopic kidney | KMT2D(602113) | NM_003482.3 | c.10394dupG | p.(Pro3466ThrfsTer2) | frameshift | Het | P | PVS1, PM2, PS2_PP | De novo | AD | KABUKI SYNDROME 1(147920) | Termination | Retrospective | Ventricular septal defect, Coarctation of aorta, Ectopic kidney | ─ |
| **197** | female | Persistent left superior vena cava, Micrognathia, FGR, Polyhydramnios | NIPBL(608667) | NM_133433.3 | c.6983C>A** | p.(Thr2328Lys) | missense | Het | LP | PS2, PM2, PP3_PM | De novo | AD | CORNELIA DE LANGE SYNDROME 1(122470) | Termination | Retrospective | Micrognathia, Intrauterine growth retardation | ─ |
| **198** | female | Dandy-Walker malformation, Hydrocephalus, Enlarged kidney, Hyperechogenic kidneys | CEP290(610142) | NM_025114.3 | c.5011C>T | p.(Gln1671Ter) | nonsense | Het | LP | PVS1, PM2 | Mat | AR | JOUBERT SYNDROME 5(610188) | Termination | Prospective | Dandy-Walker malformation, Hydrocephalus | ─ |
|  |  |  |  | NM_025114.3 | c.4156delA | p.(Ile1386SerfsTer33) | frameshift | Het | LP | PVS1, PM2 | Pat |  |  |  |  |  |  |
| **199** | male | Dandy-Walker malformation, Dilation of lateral ventricles, Multicystic kidney dysplasia, Oligohydramnios | CEP290(610142) | NM_025114.3 | c.4220G>A | p.(Trp1407Ter) | nonsense | Het | LP | PVS1, PM2 | Pat | AR | JOUBERT SYNDROME 5(610188) | Termination | Prospective | Dandy-Walker malformation, Multicystic kidney dysplasia | ─ |
|  |  |  |  | NM_025114.3 | c.2340_2341delGA | p.(Asn781Ter) | frameshift | Het | LP | PVS1, PM2 | Mat |  |  |  |  |  |  |
| **200** | male | Cystic hygroma, Arachnoid cyst, Ventriculomegaly, Micrognathia, Ventricular septal defect, Talipes equinovarus | SOX9(608160) | NM_000346.3 | c.1005G>A | p.(Trp335Ter) | nonsense | Het | P | PVS1_PS, PM2, PS2 | De novo | AD | CAMPOMELIC DYSPLASIA(114290) | Termination | Prospective | Ventriculomegaly, Micrognathia, Ventricular septal defect, Talipes equinovarus | ─ |
| **201** | female | Short long bone, Ventriculomegaly | FGFR3(134934) | NM_000142.5 | c.1138G>A | p.(Gly380Arg) | missense | Het | P | PM2, PS2_PVS, PS4_Very Strong | De novo | AD | ACHONDROPLASIA(100800) | Termination | Prospective | Short long bone, Ventriculomegaly | ─ |
| **202** | male | Increased nuchal translucency,Ventricular septal defect,Holoprosencephaly,Hypertelorism | RAF1(164760) | NM_002880.3 | c.770C>T | p.(Ser257Leu) | missense | Het | P | PP2, PM1, PM2, PS3, PS2 | De novo | AD | NOONAN SYNDROME 5(611553) | Termination | Prospective | Ventricular septal defect,Hypertelorism | ─ |
| **203** | female | Talipes equinovarus, Wrist flexion contracture, Abnormal external genitalia, Abnormality of the palm | MAGEL2(605283) | NM_019066.4 | c.2138C>A** | p.(Ser713Ter) | nonsense | Het | P | PS2, PM2, PVS1_PS | De novo | AD | SCHAAF-YANG SYNDROME(615547) | Termination | Prospective | Abnormal external genitalia, Abnormality of the palm | ─ |
| **204** | male | Thickened skin, Abnormal posturing, Open mouth | ABCA12(607800) | NM_173076.2 | c.6858delT | p.(Phe2286LeufsTer6) | frameshift | Het | P | PM2, PVS1, PM3_PP | Pat | AR | ICHTHYOSIS, CONGENITAL, AUTOSOMAL RECESSIVE 4B(242500) | Termination | Prospective | Thickened skin | ─ |
|  |  |  |  | NM_173076.2 | c.3456G>A | p.(S1152S) | synonymous | Het | LP | PM2, PM3, PM4 | Mat |  |  |  |  |  |  |
| **205** | male | Dandy-Walker malformation, Dysostosis multiplex | TMEM231(614949) | NM_001077416.2 | c.733C>T | p.(Arg245Ter) | nonsense | Het | P | PM2, PVS1, PM3 | Mat | AR | JOUBERT SYNDROME 20 | Termination | Prospective | Dandy-Walker malformation | ─ |
|  |  |  |  | NM_001077416.2 | c.597+1G>A | - | splice donor | Het | LP | PVS1,PM2 | Pat |  |  |  |  |  |  |
| **206** | female | Increased nuchal translucency, Abnormality of the hand, Intracranial hemorrhage, Widened posterior fossa, Porencephalic cyst | PLOD3(603066)* | NM_001084.4 | c.1890T>G | p.(Tyr630Ter) | nonsense | Het | LP | PVS1,PM2 | Mat | AR | BONE FRAGILITY WITH CONTRACTURES, ARTERIAL RUPTURE, AND DEAFNESS(612394) | Termination | Prospective | Abnormality of the hand, Intracranial hemorrhage | Increased nuchal translucency, Widened posterior fossa, Porencephalic cyst |
|  |  |  |  | NM_001084.4 | c.1354C>T | p.(Arg452Ter) | nonsense | Het | LP | PVS1,PM2 | Pat |  |  |  |  |  |  |
| **207** | male | Urachal cyst, Omphalocele, Hyperechogenic kidneys, Ventriculomegaly | CRB2(609720) | NM_173689.6 | c.3307T>C** | p.(Cys1103Arg) | missense | Het | LP | PP3, PM3, PM2, PP4 | Mat | AR | VENTRICULOMEGALY WITH CYSTIC KIDNEY DISEASE(219730) | Termination | Prospective | Hyperechogenic kidneys, Ventriculomegaly | Urachal cyst, Omphalocele |
|  |  |  |  | NM_173689.6 | c.3548T>A** | p.(Leu1183Ter) | nonsense | Het | P | PVS1, PM2, PP4 | Pat |  |  |  |  |  |  |
| **208** | male | Ectopic kidney, Ventricular septal defect | RET(164761) | NM_020975.4 | c.3148C>T | p.(Arg1050Ter) | nonsense | Het | LP | PVS1_PM, PM2, PS4_Moderate | Pat(asymptomatic) | AD | HIRSCHSPRUNG DISEASE, SUSCEPTIBILITY TO, 1(142623)) | Live birth | Prospective | PMID: 31471357, 22729463 | Ventricular septal defect |
| **209** | female | Multicystic kidney dysplasia, Polydactyly | BBS7(607590) | NM_176824.2 | c.1002delT | p.(Asn335IlefsTer47) | frameshift | Het | P | PVS1, PM2, PM3 | Pat | AR | BARDET-BIEDL SYNDROME 7(615984) | Termination | Prospective | Multicystic kidney dysplasia, Polydactyly | ─ |
|  |  |  |  | NM_176824.2 | c.849+1G>C** | - | splice donor | Het | P | PVS1, PM2, PM3 | Mat |  |  |  |  |  |  |
| **210** | female | Omphalocele, Hydronephrosis, Micromelia | WDR60(615462) | NM_018051.4 | c.1925G>A** | p.(Arg642Gln) | missense | Het | LP | PM2, PM3, PP3, PP4 | Pat | AR | SHORT-RIB THORACIC DYSPLASIA 8 WITH OR WITHOUT POLYDACTYLY(615503) | Termination | Prospective | Omphalocele, Hydronephrosis, Micromelia | ─ |
|  |  |  |  | NM_018051.4 | c.2095-1G>A** | - | splice acceptor | Het | P | PVS1, PM2, PP4 | Mat |  |  |  |  |  |  |
| **211** | female | Multicystic kidney dysplasia, Ascites, Oligohydramnios | BBS2(606151) | NM_031885.3 | c.2107C>T | p.(Arg703Ter) | nonsense | Het | LP | PM2, PVS1_PM, PM3 | Pat | AR | BARDET-BIEDL SYNDROME 2(615981) | Termination | Prospective | Multicystic kidney dysplasia | Ascites |
|  |  |  |  | NM_031885.3 | c.534+1G>T | - | splice donor | Het | LP | PM2, PVS1_PM, PM3 | Mat |  |  |  |  |  |  |
| **212** | female | Pulmonary hypoplasia, Hyperechogenic kidneys, Micrognathia, Hydrops fetalis | RAPSN(601592) | NM_005055.5 | c.280G>A | p.(Glu94Lys) | missense | Het | LP | PM2_PP, PM3, PP1_PS, PP4 | Pat | AR | FETAL AKINESIA DEFORMATION SEQUENCE 2(618388) | Termination | Prospective | Pulmonary hypoplasia, Micrognathia | ─ |
|  |  |  |  | NM_005055.5 | c.149_153delinsAGATGGGCCGCTACAAGGAGATGG** | p.Val50fs | frameshift | Het | P | PVS1, PM2, PP4 | Mat |  |  |  |  |  |  |
| **213** | male | Hydrops fetalis, Micrognathia, Talipes equinovarus, Polyhydramnios | ACTA1(102610) | NM_001100.3 | c.110T>G** | p.(Val37Gly) | missense | Het | LP | PM2, PP3, PS2_PP, PM5 | De novo | AD | NEMALINE MYOPATHY 3(161800) | Termination | Prospective | Micrognathia, Polyhydramnios | Hydrops fetalis |
| **214** | male | Cleft palate, Cleft lip, Persistent left superior vena cava, Widened posterior fossa | MID1(300552) | NM_000381.4 | c.1798dupC | p.(His600ProfsTer12) | frameshift | Hemi | LP | PVS1_PS, PM2, PS4_PM | Mat(affected) | XR | OPITZ GBBB SYNDROME, TYPE I(300000) | Termination | Prospective | Cleft palate, Cleft lip | ─ |
| **215** | male | Increased nuchal translucency, Ascites, Hyperechogenic kidneys, Tricuspid regurgitation | FLNA(300017) | NM_001110556.1 | c.1538_1539 delGGinsTA** | (p.Gly513Val) | In-frame | Hemi | LP | PM1, PM2, PP3_PM | Mat | XL | MELNICK-NEEDLES SYNDROME(309350) | Termination | Prospective | Tricuspid regurgitation; urogenital systems | Increased nuchal translucency, Ascites |
| **216** | male | Intrauterine growth retardation, Pleural effusion, Decreased fetal movement | GBA(606463) | NM_000157.4 | c.1448T>C(Sanger sequencing) | p.(Leu483Pro) | missense | Het | P | PM3_PVS, PP3, PS3_Moderate, PM5 | Mat | AR | GAUCHER DISEASE, TYPE I(230800) | Termination | Prospective | Intrauterine growth retardation, Decreased fetal movement | ─ |
|  |  |  |  | NM_000157.4 | c.521A>G | p.(Tyr174Cys) | missense | Het | LP | PS3_PM, PM2, PM3, PP3 | Pat |  |  |  |  |  |  |
| **217** | female | Ventriculomegaly, Right renal agenesis, Left renal duplication, Ventricular septal defect, Persistent left superior vena cava,Small for gestational age | FANCI(611360) | NM_001113378.1 | c.3015G>C** | p.(Gln1005His) | missense | Het | LP | PM2, PM3, PP3, PP4 | Pat | AR | FANCONI ANEMIA, COMPLEMENTATION GROUP I(609053) | Termination | Prospective | Ventriculomegaly, Ventricular septal defect,Hypoplastic kidneys | ─ |
|  |  |  |  | NM_001113378.1 | c.3187-2A>G** | - | splice acceptor | Het | P | PVS1, PM2, PP4 | Mat |  |  |  |  |  |  |
| **218** | female | Ventriculomegaly, Tetralogy of Fallot, Right aortic arch | MTOR(601231)* | NM_004958.3 | c.7255G>A | p.(Glu2419Lys) | missense | Het | P | PS2, PM2, PP3, PP2 | De novo | AD | SMITH-KINGSMORE SYNDROME(616638) | Termination | Prospective | Ventriculomegaly | Tetralogy of Fallot, Right aortic arch |
| **219** | male | Polyhydramnios, Short long bone, Cardiomegaly | KMT2A(159555)* | NM_001197104.1 | c.4219-1G>A** | - | splice acceptor | Het | P | PM2, PVS1, PS2_PM | De novo | AD | WIEDEMANN-STEINER SYNDROME(605130) | Live birth | Prospective | Short long bone | Polyhydramnios, Cardiomegaly |
| **220** | male | Dilation of lateral ventricles, Ventricular septal defect, Dilated third ventricle | PEX1(602136) | NM_000466.2 | c.1585delC** | p.(Gln529LysfsTer4) | frameshift | Het | LP | PVS1, PM2 | Mat | AR | PEROXISOME BIOGENESIS DISORDER 1A (ZELLWEGER)(214100) | Termination | Prospective | Ventricular septal defect | Dilation of lateral ventricles, Dilated third ventricle |
|  |  |  |  | NM_000466.2 | c.5G>A | p.(Trp2Ter) | nonsense | Het | LP | PVS1, PM2 | Pat |  |  |  |  |  |  |
| **221** | male | Aplasia of the nasal bone, Abnormality of ductus venosus blood flow,Single umbilical artery, Ventriculomegaly, Dandy-Walker malformation, Congenital diaphragmatic hernia | ARID1A(603024)* | NM_006015.4 | c.5853dupC** | p.(Ile1952HisfsTer11) | missense | Het | P | PS2, PM2, PVS1_PS | De novo | AD | COFFIN-SIRIS SYNDROME 2(614607) | Termination | Prospective | Ventriculomegaly, Dandy-Walker malformation | ─ |
| **222** | male | Ventriculomegaly, Frontal bossing,Lemon sign, Talipes equinovarus,Renal agenesis, Aplasia/Hypoplasia involving the vertebral column | FGFR2(176943) | NM_000141.4 | c.1019A>G | p.Try340Cys | missense | Het | P | PP3, PM2, PS4_PM, PS2, PM5, PM1 | De novo | AD | APERT SYNDROME(101200) | Termination | Prospective | Ventriculomegaly, Talipes equinovarus ,Frontal bossing | ─ |
| **223** | female | Cleft palate, Short femur | MYH3(160720) | NM_002470.3 | c.727_729delTCC | p.(Ser243del) | In-frame | Het | LP | PM2, PM4_PP, PS2_PM, PP1, PS3_PP | De novo | AD | CONTRACTURES, PTERYGIA, AND SPONDYLOCARPOTARSAL FUSION SYNDROME 1A(178110) | Termination | Prospective | Cleft palate | ─ |
| **224** | female | Ventriculomegaly, Pleural effusion | KRAS(190070) | NM_033360.3 | c.101C>T | p.(Pro34Leu) | missense | Het | P | PS2, PM2, PM5, PP3, PP2 | De novo | AD | NOONAN SYNDROME 3(609942) | Termination | Prospective | Ventriculomegaly | ─ |
| **225** | male | Supraventricular tachycardia, Enlarged cisterna magna, Ascites, Pleural effusion, Polyhydramnios | CSNK2A1(115440)* | NM_001895.3 | c.838C>T** | p.(Arg280Ter) | nonsense | Het | LP | PVS1_PS, PM1, PM2 | De novo | AD | OKUR-CHUNG NEURODEVELOPMENTAL SYNDROME(617062) | Termination | Prospective | Cardiovascular system; PMID: 29568000 | Supraventricular tachycardia, Pleural effusion, Polyhydramnios |
| **226** | female | Pulmonary hypoplasia, Right renal agenesis, Left renal dysplasia, Oligohydramnios | **PBX1(176310)** | NM_002585.3 | c.973_974delTC** | p.(Ser325AlafsTer14) | frameshift | Het | P | PVS1, PM2, PS2_PP | De novo | AD | CONGENITAL ANOMALIES OF KIDNEY AND URINARY TRACT SYNDROME WITH OR WITHOUT HEARING LOSS, ABNORMAL EARS, OR DEVELOPMENTAL DELAY(617641) | Termination | Prospective | Renal agenesis, Oligohydramnios | Pulmonary hypoplasia |
| **227** | female | Omphalocele, Tricuspid regurgitation, Polyhydramnios, Short long bone, Hepatomegaly | CDKN1C(600856) | NM_000076.2 | c.827_828delinsAA** | p.(Phe276Ter) | frameshift | Het | LP | PVS1_PS, PM2, PP4 | Mat(asymptomatic) | AD | BECKWITH-WIEDEMANN SYNDROME; BWS(130650) | Termination | Prospective | Omphalocele, Hepatomegaly | ─ |
| **228** | male | Microcephaly, Transverse facial cleft, FGR | ZEB2(605802) | NM_014795.3 | c.436G>T** | p.(Glu146Ter) | nonsense | Het | P | PVS1, PM2, PS2_PM | De novo | AD | MOWAT-WILSON SYNDROME(235730) | Live birth | Prospective | Microcephaly | Transverse facial cleft |
| **Others** | |  |  |  |  |  |  |  |  |  |  |  |  |  |  |  |  |
| **229** | male | Large for gestational age, Polyhydramnios | **CLCN5(300008)*** | NM_001127898.3 | c.934-1G>T** | - | splice acceptor | Hemi | LP | PVS1_PS, PM1, PM2 | Mat | XR | DENT DISEASE 1(300009) | Live birth | Prospective | PMID: 18540256, 27174143 | ─ |

**Bold genes:** the 11 genes outside the list in PAGE study (DDG2P 1856 genes downloaded in July 2019 plus 117 prenatal associated genes).

* Genes firstly reported in prenatal cases in this study.

** Novel variants identified in this study.

^#^ Cases obtained upgrading from negative to P/LP due to new disease gene identified in reanalysis.

**Additional file 2 Table S4. Diagnostic rates in different malformation subgroups**

| **Malformation categories** | **Positive diagnostic rate %** | | |  | **Inconclusive rate %** | |  |
| --- | --- | --- | --- | --- | --- | --- | --- |
|  | **Total** | **Retrospective cohort** | **Prospective cohort** |  | **Total** | **Retrospective cohort** | **Prospective cohort** |
| Central nervous | 12.6 (33/262) | 11.4 (15/132) | 13.8 (18/130) |  | 8.0 (21/262) | 6.1 (8/132) | 10.0 (13/130) |
| Facial | 9.7 (12/124) | 8.6 (5/58) | 10.6 (7/66) |  | 10.5 (13/124) | 12.1 (7/58) | 9.1 (6/66) |
| Chest | 0 (0/46) | 0 (0/8) | 0 (0/38) |  | 8.7 (4/46) | 25 (2/8) | 5.3 (2/38) |
| Cardiovascular | 12.8 (37/289) | 15.8 (16/101) | 11.2 (21/188) |  | 6.6 (19/289) | 9.9 (10/101) | 4.8 (9/188) |
| Abdominal | 3.5 (2/57) | 6.3 (1/16) | 2.4 (1/41) |  | 5.3 (3/57) | 6.3 (1/16) | 4.9 (2/41) |
| Urogenital | 8.1 (15/185) | 9.4 (6/64) | 7.4 (9/121) |  | 6.5 (12/185) | 7.8 (5/64) | 5.8 (7/121) |
| Skeletal | 30.4 (63/207) | 39.7 (27/68) | 25.9 (36/139) |  | 7.7 (16/207) | 4.4 (3/68) | 9.4 (13/139) |
| FGR | 3.3 (2/61) | 9.1 (1/11) | 2.0 (1/50) |  | 8.2 (5/61) | 9.1 (1/11) | 8.0 (4/50) |
| Isolated Hydrops | 6.9 (2/29) | 0 (0/4) | 8.0 (2/25) |  | 24.1 (7/29) | 25 (1/4) | 24 (6/25) |
| Increased NT and cystic hygroma | 5.0 (6/121) | 8.7 (2/23) | 4.1 (4/98) |  | 1.7 (2/121) | 8.7 (2/23) | 0 (0/98) |
| Others | 4.8 (1/21) | 0 (0/4) | 5.9 (1/17) |  | 0 (0/21) | 0 (0/4) | 0 (0/17) |
| Multisystem | 25.9 (56/216) | 32.9 (25/76) | 22.1 (31/140) |  | 12.0 (26/216) | 10.5 (8/76) | 14.3 (20/140) |
| **Total** | **14.2 (229/1618)** | **17.3 (98/565)** | **12.4 (131/1053)** |  | **8.1 (131/1618)** | **8.5 (48/565)** | **7.9 (83/1053)** |

**Additional file 2 Table S5. Diagnostic rates in relation to NT measurement range**

| **NT (mm)** | **Isolated group*** | |  | **Associated group** | |
| --- | --- | --- | --- | --- | --- |
|  | Number of cases | Diagnostic rate % |  | Number of cases | Diagnostic rate % |
| **3.0-3.4** | NA | NA |  | 522 | 12.8 (67/522) |
| **3.5-3.9** | 42 | 2.4 (1/42) |  | 6 | 16.7 (1/6) |
| **4.0-4.4** | 21 | 4.8 (1/21) |  | 7 | 28.6 (2/7) |
| **4.5-4.9** | 15 | 6.7 (1/15) |  | 6 | 50.0 (3/6) |
| **≥5.0** | 43 | 7.0 (3/43) |  | 28 | 21.4 (6/28) |
| **Total (increased NT)** | **121** | 1. **(6/121)** |  | **47** | **25.5 (12/47)** |

* Pearson correlation coefficient R = 0.913 with p value = 0.08

**Additional file 2 Table S6. Number of variants analyzed in step 1 and 2- case by case**

| **No.** | **Malformation classification** | **Genotype-driven** | |  | **Phenotype-driven** | | **Total Number of variants interpreted as irrelevant after quick review** | **Total Number of variants closely Reviewed (step 1+2)** | **Total Number of variants interpreted as positive diagnostic** | **Total Number of variants interpreted as potentially relevant (Inconclusive)** | **Total Number of variants interpreted as irrelevant after close review** | **Overall Conclusion** |
| --- | --- | --- | --- | --- | --- | --- | --- | --- | --- | --- | --- | --- |
|  |  | Total number of variants based on origin/ zygosity/pathogenicity | Number of variants for focused clinical correlation |  | Total number of variants based on HPO matching | Additional number of variants closely reviewed in this step |  |  |  |  |  |  |
| 1 | Central nervous | 3 | 2 |  | 112 | 3 | 110 | 5 | 1 | 0 | 4 | Positive |
| 2 | Central nervous | 6 | 1 |  | 20 | 1 | 24 | 2 | 1 | 0 | 1 | Positive |
| 3 | Central nervous | 0 | 0 |  | 29 | 0 | 29 | 0 | 0 | 0 | 0 | Negative |
| 4 | Central nervous | 2 | 0 |  | 19 | 0 | 21 | 0 | 0 | 0 | 0 | Negative |
| 5 | Central nervous | 2 | 1 |  | 46 | 1 | 46 | 2 | 1 | 0 | 1 | Positive |
| 6 | Central nervous | 2 | 2 |  | 40 | 1 | 39 | 3 | 0 | 1 | 2 | Inconclusive |
| 7 | Central nervous | 2 | 0 |  | 10 | 0 | 12 | 0 | 0 | 0 | 0 | Negative |
| 8 | Central nervous | 11 | 3 |  | 11 | 1 | 18 | 4 | 1 | 0 | 3 | Positive |
| 9 | Central nervous | 5 | 2 |  | 9 | 0 | 12 | 2 | 0 | 0 | 2 | Negative |
| 10 | Central nervous | 7 | 1 |  | 53 | 2 | 57 | 3 | 1 | 0 | 2 | Positive |
| 11 | Central nervous | 4 | 0 |  | 5 | 0 | 9 | 0 | 0 | 0 | 0 | Negative |
| 12 | Central nervous | 3 | 0 |  | 6 | 0 | 9 | 0 | 0 | 0 | 0 | Negative |
| 13 | Central nervous | 1 | 0 |  | 12 | 0 | 13 | 0 | 0 | 0 | 0 | Negative |
| 14 | Central nervous | 7 | 2 |  | 10 | 0 | 15 | 2 | 0 | 0 | 2 | Negative |
| 15 | Central nervous | 3 | 1 |  | 25 | 0 | 27 | 1 | 1 | 0 | 0 | Positive |
| 16 | Central nervous | 4 | 2 |  | 51 | 0 | 53 | 2 | 0 | 0 | 2 | Negative |
| 17 | Central nervous | 6 | 0 |  | 14 | 0 | 20 | 0 | 0 | 0 | 0 | Negative |
| 18 | Central nervous | 7 | 0 |  | 21 | 2 | 26 | 2 | 0 | 0 | 2 | Negative |
| 19 | Central nervous | 3 | 0 |  | 34 | 4 | 33 | 4 | 0 | 0 | 4 | Negative |
| 20 | Central nervous | 4 | 2 |  | 18 | 0 | 20 | 2 | 0 | 0 | 2 | Negative |
| 21 | Central nervous | 3 | 0 |  | 18 | 0 | 21 | 0 | 0 | 0 | 0 | Negative |
| 22 | Central nervous | 2 | 0 |  | 95 | 2 | 95 | 2 | 0 | 0 | 2 | Negative |
| 23 | Central nervous | 7 | 0 |  | 9 | 0 | 16 | 0 | 0 | 0 | 0 | Negative |
| 24 | Central nervous | 3 | 0 |  | 10 | 2 | 11 | 2 | 0 | 0 | 2 | Negative |
| 25 | Central nervous | 5 | 0 |  | 11 | 1 | 15 | 1 | 0 | 0 | 1 | Negative |
| 26 | Central nervous | 3 | 0 |  | 6 | 0 | 9 | 0 | 0 | 0 | 0 | Negative |
| 27 | Central nervous | 4 | 0 |  | 7 | 0 | 11 | 0 | 0 | 0 | 0 | Negative |
| 28 | Central nervous | 5 | 1 |  | 37 | 0 | 41 | 1 | 0 | 0 | 1 | Negative |
| 29 | Central nervous | 4 | 2 |  | 63 | 0 | 65 | 2 | 0 | 0 | 2 | Negative |
| 30 | Central nervous | 4 | 0 |  | 24 | 1 | 27 | 1 | 0 | 0 | 1 | Negative |
| 31 | Central nervous | 13 | 2 |  | 27 | 0 | 38 | 2 | 0 | 0 | 2 | Negative |
| 32 | Central nervous | 4 | 0 |  | 10 | 0 | 14 | 0 | 0 | 0 | 0 | Negative |
| 33 | Central nervous | 6 | 3 |  | 57 | 3 | 57 | 6 | 1 | 0 | 5 | Positive |
| 34 | Central nervous | 10 | 2 |  | 32 | 0 | 40 | 2 | 0 | 0 | 2 | Negative |
| 35 | Central nervous | 9 | 4 |  | 43 | 0 | 48 | 4 | 2 | 0 | 2 | Positive |
| 36 | Central nervous | 5 | 2 |  | 102 | 4 | 101 | 6 | 1 | 0 | 5 | Positive |
| 37 | Central nervous | 4 | 2 |  | 110 | 3 | 109 | 5 | 0 | 2 | 3 | Inconclusive |
| 38 | Central nervous | 3 | 1 |  | 21 | 1 | 22 | 2 | 1 | 0 | 1 | Positive |
| 39 | Central nervous | 8 | 5 |  | 97 | 1 | 99 | 6 | 0 | 2 | 4 | Inconclusive |
| 40 | Central nervous | 1 | 1 |  | 29 | 0 | 29 | 1 | 0 | 1 | 0 | Inconclusive |
| 41 | Central nervous | 6 | 1 |  | 25 | 0 | 30 | 1 | 1 | 0 | 0 | Positive |
| 42 | Central nervous | 9 | 1 |  | 93 | 4 | 97 | 5 | 0 | 1 | 4 | Inconclusive |
| 43 | Central nervous | 6 | 4 |  | 85 | 3 | 84 | 7 | 0 | 2 | 5 | Inconclusive |
| 44 | Central nervous | 3 | 1 |  | 64 | 0 | 66 | 1 | 1 | 0 | 0 | Positive |
| 45 | Central nervous | 1 | 0 |  | 25 | 2 | 24 | 2 | 0 | 0 | 2 | Negative |
| 46 | Central nervous | 2 | 0 |  | 92 | 2 | 92 | 2 | 0 | 0 | 2 | Negative |
| 47 | Central nervous | 7 | 4 |  | 83 | 1 | 85 | 5 | 0 | 0 | 5 | Negative |
| 48 | Central nervous | 2 | 2 |  | 151 | 2 | 149 | 4 | 0 | 0 | 4 | Negative |
| 49 | Central nervous | 3 | 1 |  | 9 | 1 | 10 | 2 | 0 | 1 | 1 | Inconclusive |
| 50 | Central nervous | 4 | 2 |  | 91 | 2 | 91 | 4 | 2 | 0 | 2 | Positive |
| 51 | Facial | 8 | 0 |  | 8 | 1 | 15 | 1 | 0 | 0 | 1 | Negative |
| 52 | Facial | 4 | 2 |  | 37 | 2 | 37 | 4 | 0 | 2 | 2 | Inconclusive |
| 53 | Facial | 9 | 0 |  | 5 | 0 | 14 | 0 | 0 | 0 | 0 | Negative |
| 54 | Facial | 2 | 0 |  | 10 | 0 | 12 | 0 | 0 | 0 | 0 | Negative |
| 55 | Facial | 3 | 1 |  | 30 | 2 | 30 | 3 | 1 | 0 | 2 | Positive |
| 56 | Facial | 5 | 2 |  | 7 | 0 | 10 | 2 | 0 | 2 | 0 | Inconclusive |
| 57 | Facial | 12 | 6 |  | 7 | 1 | 12 | 7 | 0 | 1 | 6 | Inconclusive |
| 58 | Facial | 4 | 2 |  | 11 | 2 | 11 | 4 | 1 | 0 | 3 | Positive |
| 59 | Facial | 8 | 0 |  | 11 | 1 | 18 | 1 | 1 | 0 | 0 | Positive |
| 60 | Facial | 5 | 0 |  | 16 | 0 | 21 | 0 | 0 | 0 | 0 | Negative |
| 61 | Facial | 15 | 0 |  | 4 | 0 | 19 | 0 | 0 | 0 | 0 | Negative |
| 62 | Facial | 5 | 0 |  | 6 | 1 | 10 | 1 | 0 | 0 | 1 | Negative |
| 63 | Facial | 4 | 0 |  | 6 | 0 | 10 | 0 | 0 | 0 | 0 | Negative |
| 64 | Facial | 11 | 1 |  | 5 | 0 | 15 | 1 | 0 | 0 | 1 | Negative |
| 65 | Facial | 6 | 2 |  | 5 | 0 | 9 | 2 | 0 | 0 | 2 | Negative |
| 66 | Facial | 6 | 2 |  | 17 | 1 | 20 | 3 | 0 | 2 | 1 | Inconclusive |
| 67 | Facial | 9 | 2 |  | 5 | 1 | 11 | 3 | 0 | 0 | 3 | Negative |
| 68 | Facial | 8 | 4 |  | 94 | 3 | 95 | 7 | 0 | 0 | 7 | Negative |
| 69 | Facial | 4 | 0 |  | 67 | 4 | 67 | 4 | 0 | 0 | 4 | Negative |
| 70 | Facial | 6 | 2 |  | 30 | 0 | 34 | 2 | 0 | 0 | 2 | Negative |
| 71 | Facial | 4 | 0 |  | 23 | 1 | 26 | 1 | 0 | 0 | 1 | Negative |
| 72 | Facial | 5 | 2 |  | 70 | 0 | 73 | 2 | 0 | 0 | 2 | Negative |
| 73 | Facial | 1 | 0 |  | 12 | 1 | 12 | 1 | 0 | 0 | 1 | Negative |
| 74 | Facial | 2 | 0 |  | 65 | 3 | 64 | 3 | 0 | 0 | 3 | Negative |
| 75 | Facial | 2 | 0 |  | 83 | 4 | 81 | 4 | 0 | 0 | 4 | Negative |
| 76 | Facial | 8 | 3 |  | 86 | 0 | 91 | 3 | 0 | 1 | 2 | Inconclusive |
| 77 | Facial | 8 | 2 |  | 11 | 0 | 17 | 2 | 0 | 0 | 2 | Negative |
| 78 | Facial | 5 | 0 |  | 27 | 0 | 32 | 0 | 0 | 0 | 0 | Negative |
| 79 | Facial | 4 | 2 |  | 65 | 1 | 66 | 3 | 1 | 0 | 2 | Positive |
| 80 | Facial | 9 | 0 |  | 11 | 0 | 20 | 0 | 0 | 0 | 0 | Negative |
| 81 | Facial | 6 | 0 |  | 15 | 0 | 21 | 0 | 0 | 0 | 0 | Negative |
| 82 | Facial | 10 | 5 |  | 28 | 2 | 31 | 7 | 0 | 0 | 7 | Negative |
| 83 | Facial | 8 | 3 |  | 38 | 2 | 41 | 5 | 1 | 0 | 4 | Positive |
| 84 | Facial | 7 | 1 |  | 87 | 3 | 90 | 4 | 1 | 0 | 3 | Positive |
| 85 | Facial | 8 | 1 |  | 27 | 0 | 34 | 1 | 0 | 1 | 0 | Inconclusive |
| 86 | Facial | 9 | 0 |  | 8 | 0 | 17 | 0 | 0 | 0 | 0 | Negative |
| 87 | Facial | 5 | 2 |  | 41 | 0 | 44 | 2 | 0 | 2 | 0 | Inconclusive |
| 88 | Facial | 1 | 0 |  | 12 | 1 | 12 | 1 | 0 | 0 | 1 | Negative |
| 89 | Facial | 7 | 4 |  | 14 | 0 | 17 | 4 | 1 | 0 | 3 | Positive |
| 90 | Facial | 4 | 0 |  | 55 | 1 | 58 | 1 | 0 | 0 | 1 | Negative |
| 91 | Facial | 6 | 0 |  | 4 | 0 | 10 | 0 | 0 | 0 | 0 | Negative |
| 92 | Facial | 8 | 0 |  | 13 | 2 | 19 | 2 | 0 | 1 | 1 | Inconclusive |
| 93 | Facial | 4 | 0 |  | 12 | 1 | 15 | 1 | 0 | 0 | 1 | Negative |
| 94 | Facial | 4 | 0 |  | 55 | 2 | 57 | 2 | 0 | 0 | 2 | Negative |
| 95 | Facial | 4 | 0 |  | 8 | 0 | 12 | 0 | 0 | 0 | 0 | Negative |
| 96 | Facial | 3 | 0 |  | 12 | 2 | 13 | 2 | 0 | 0 | 2 | Negative |
| 97 | Facial | 5 | 3 |  | 51 | 2 | 51 | 5 | 1 | 0 | 4 | Positive |
| 98 | Facial | 8 | 0 |  | 13 | 1 | 20 | 1 | 0 | 1 | 0 | Inconclusive |
| 99 | Facial | 2 | 0 |  | 3 | 1 | 4 | 1 | 1 | 0 | 0 | Positive |
| 100 | Facial | 8 | 3 |  | 36 | 2 | 39 | 5 | 1 | 0 | 4 | Positive |
| 101 | Chest | 11 | 0 |  | 3 | 0 | 14 | 0 | 0 | 0 | 0 | Negative |
| 102 | Chest | 18 | 5 |  | 50 | 5 | 58 | 10 | 0 | 2 | 8 | Inconclusive |
| 103 | Chest | 4 | 0 |  | 2 | 0 | 6 | 0 | 0 | 0 | 0 | Negative |
| 104 | Chest | 9 | 0 |  | 1 | 0 | 10 | 0 | 0 | 0 | 0 | Negative |
| 105 | Chest | 3 | 1 |  | 3 | 1 | 4 | 2 | 0 | 0 | 2 | Negative |
| 106 | Chest | 6 | 0 |  | 34 | 0 | 40 | 0 | 0 | 0 | 0 | Negative |
| 107 | Chest | 8 | 0 |  | 9 | 2 | 15 | 2 | 0 | 0 | 2 | Negative |
| 108 | Chest | 5 | 0 |  | 15 | 0 | 20 | 0 | 0 | 0 | 0 | Negative |
| 109 | Chest | 3 | 2 |  | 43 | 1 | 43 | 3 | 0 | 0 | 3 | Negative |
| 110 | Chest | 8 | 0 |  | 59 | 1 | 66 | 1 | 0 | 0 | 1 | Negative |
| 111 | Chest | 5 | 3 |  | 32 | 0 | 34 | 3 | 0 | 0 | 3 | Negative |
| 112 | Chest | 4 | 2 |  | 8 | 0 | 10 | 2 | 0 | 0 | 2 | Negative |
| 113 | Chest | 5 | 2 |  | 49 | 2 | 50 | 4 | 0 | 0 | 4 | Negative |
| 114 | Chest | 4 | 0 |  | 1 | 0 | 5 | 0 | 0 | 0 | 0 | Negative |
| 115 | Chest | 2 | 0 |  | 1 | 0 | 3 | 0 | 0 | 0 | 0 | Negative |
| 116 | Chest | 3 | 0 |  | 1 | 0 | 4 | 0 | 0 | 0 | 0 | Negative |
| 117 | Chest | 7 | 1 |  | 36 | 2 | 40 | 3 | 0 | 0 | 3 | Negative |
| 118 | Chest | 5 | 3 |  | 72 | 1 | 73 | 4 | 0 | 1 | 3 | Inconclusive |
| 119 | Chest | 5 | 0 |  | 31 | 0 | 36 | 0 | 0 | 0 | 0 | Negative |
| 120 | Chest | 4 | 2 |  | 29 | 1 | 30 | 3 | 0 | 0 | 3 | Negative |
| 121 | Chest | 3 | 0 |  | 51 | 0 | 54 | 0 | 0 | 0 | 0 | Negative |
| 122 | Chest | 3 | 0 |  | 5 | 0 | 8 | 0 | 0 | 0 | 0 | Negative |
| 123 | Chest | 5 | 0 |  | 24 | 1 | 28 | 1 | 0 | 0 | 1 | Negative |
| 124 | Chest | 3 | 0 |  | 2 | 0 | 5 | 0 | 0 | 0 | 0 | Negative |
| 125 | Chest | 2 | 0 |  | 44 | 0 | 46 | 0 | 0 | 0 | 0 | Negative |
| 126 | Chest | 5 | 0 |  | 2 | 0 | 7 | 0 | 0 | 0 | 0 | Negative |
| 127 | Chest | 6 | 0 |  | 5 | 0 | 11 | 0 | 0 | 0 | 0 | Negative |
| 128 | Chest | 2 | 0 |  | 1 | 0 | 3 | 0 | 0 | 0 | 0 | Negative |
| 129 | Chest | 12 | 0 |  | 4 | 0 | 16 | 0 | 0 | 0 | 0 | Negative |
| 130 | Chest | 3 | 0 |  | 1 | 0 | 4 | 0 | 0 | 0 | 0 | Negative |
| 131 | Chest | 6 | 2 |  | 65 | 1 | 68 | 3 | 0 | 0 | 3 | Inconclusive |
| 132 | Chest | 4 | 0 |  | 22 | 2 | 24 | 2 | 0 | 0 | 2 | Negative |
| 133 | Chest | 4 | 0 |  | 34 | 0 | 38 | 0 | 0 | 0 | 0 | Negative |
| 134 | Chest | 6 | 0 |  | 43 | 3 | 46 | 3 | 0 | 0 | 3 | Negative |
| 135 | Chest | 4 | 0 |  | 31 | 0 | 35 | 0 | 0 | 0 | 0 | Negative |
| 136 | Chest | 11 | 0 |  | 44 | 2 | 53 | 2 | 0 | 0 | 2 | Negative |
| 137 | Chest | 12 | 0 |  | 28 | 0 | 40 | 0 | 0 | 0 | 0 | Negative |
| 138 | Chest | 2 | 0 |  | 17 | 0 | 19 | 0 | 0 | 0 | 0 | Negative |
| 139 | Chest | 4 | 0 |  | 59 | 0 | 63 | 0 | 0 | 0 | 0 | Negative |
| 140 | Chest | 0 | 0 |  | 20 | 1 | 19 | 1 | 0 | 0 | 1 | Negative |
| 141 | Chest | 4 | 0 |  | 28 | 2 | 30 | 2 | 0 | 0 | 2 | Negative |
| 142 | Chest | 6 | 0 |  | 13 | 0 | 19 | 0 | 0 | 0 | 0 | Negative |
| 143 | Chest | 3 | 1 |  | 67 | 1 | 68 | 2 | 0 | 1 | 1 | Inconclusive |
| 144 | Chest | 4 | 0 |  | 27 | 0 | 31 | 0 | 0 | 0 | 0 | Negative |
| 145 | Chest | 4 | 2 |  | 52 | 0 | 54 | 2 | 0 | 0 | 2 | Negative |
| 146 | Chest | 6 | 0 |  | 45 | 1 | 50 | 1 | 0 | 0 | 1 | Negative |
| 147 | Cardiovascular | 3 | 0 |  | 7 | 0 | 10 | 0 | 0 | 0 | 0 | Negative |
| 148 | Cardiovascular | 12 | 0 |  | 2 | 0 | 14 | 0 | 0 | 0 | 0 | Negative |
| 149 | Cardiovascular | 7 | 0 |  | 23 | 0 | 30 | 0 | 0 | 0 | 0 | Negative |
| 150 | Cardiovascular | 11 | 0 |  | 9 | 1 | 19 | 1 | 0 | 0 | 1 | Negative |
| 151 | Cardiovascular | 4 | 2 |  | 7 | 0 | 9 | 2 | 0 | 0 | 2 | Negative |
| 152 | Cardiovascular | 7 | 0 |  | 9 | 0 | 16 | 0 | 0 | 0 | 0 | Negative |
| 153 | Cardiovascular | 5 | 0 |  | 11 | 1 | 15 | 1 | 0 | 0 | 1 | Negative |
| 154 | Cardiovascular | 0 | 0 |  | 16 | 0 | 16 | 0 | 0 | 0 | 0 | Negative |
| 155 | Cardiovascular | 5 | 0 |  | 2 | 0 | 7 | 0 | 0 | 0 | 0 | Negative |
| 156 | Cardiovascular | 4 | 0 |  | 3 | 0 | 7 | 0 | 0 | 0 | 0 | Negative |
| 157 | Cardiovascular | 6 | 2 |  | 21 | 2 | 23 | 4 | 0 | 0 | 4 | Negative |
| 158 | Cardiovascular | 2 | 0 |  | 2 | 1 | 3 | 1 | 0 | 0 | 1 | Negative |
| 159 | Cardiovascular | 5 | 1 |  | 54 | 0 | 58 | 1 | 1 | 0 | 0 | Positive |
| 160 | Cardiovascular | 2 | 0 |  | 2 | 0 | 4 | 0 | 0 | 0 | 0 | Negative |
| 161 | Cardiovascular | 7 | 0 |  | 5 | 1 | 11 | 1 | 0 | 0 | 1 | Negative |
| 162 | Cardiovascular | 2 | 0 |  | 48 | 3 | 47 | 3 | 0 | 0 | 3 | Negative |
| 163 | Cardiovascular | 10 | 0 |  | 7 | 1 | 16 | 1 | 0 | 0 | 1 | Negative |
| 164 | Cardiovascular | 6 | 0 |  | 10 | 1 | 15 | 1 | 0 | 0 | 1 | Negative |
| 165 | Cardiovascular | 6 | 2 |  | 4 | 1 | 7 | 3 | 0 | 0 | 3 | Negative |
| 166 | Cardiovascular | 7 | 2 |  | 8 | 0 | 13 | 2 | 0 | 2 | 0 | Inconclusive |
| 167 | Cardiovascular | 6 | 0 |  | 6 | 0 | 12 | 0 | 0 | 0 | 0 | Negative |
| 168 | Cardiovascular | 3 | 0 |  | 44 | 0 | 47 | 0 | 0 | 0 | 0 | Negative |
| 169 | Cardiovascular | 7 | 0 |  | 7 | 0 | 14 | 0 | 0 | 0 | 0 | Negative |
| 170 | Cardiovascular | 4 | 0 |  | 41 | 4 | 41 | 4 | 0 | 0 | 4 | Negative |
| 171 | Cardiovascular | 6 | 1 |  | 4 | 0 | 9 | 1 | 1 | 0 | 0 | Positive |
| 172 | Cardiovascular | 2 | 0 |  | 4 | 0 | 6 | 0 | 0 | 0 | 0 | Negative |
| 173 | Cardiovascular | 8 | 2 |  | 7 | 0 | 13 | 2 | 0 | 0 | 2 | Negative |
| 174 | Cardiovascular | 4 | 3 |  | 14 | 0 | 15 | 3 | 0 | 0 | 3 | Negative |
| 175 | Cardiovascular | 10 | 4 |  | 10 | 0 | 16 | 4 | 0 | 0 | 4 | Negative |
| 176 | Cardiovascular | 5 | 0 |  | 6 | 1 | 10 | 1 | 0 | 0 | 1 | Negative |
| 177 | Cardiovascular | 7 | 2 |  | 6 | 2 | 9 | 4 | 0 | 0 | 4 | Negative |
| 178 | Cardiovascular | 6 | 0 |  | 12 | 0 | 18 | 0 | 0 | 0 | 0 | Negative |
| 179 | Cardiovascular | 10 | 2 |  | 18 | 0 | 26 | 2 | 0 | 0 | 2 | Negative |
| 180 | Cardiovascular | 8 | 0 |  | 16 | 1 | 23 | 1 | 0 | 0 | 1 | Negative |
| 181 | Cardiovascular | 6 | 0 |  | 58 | 1 | 63 | 1 | 0 | 0 | 1 | Negative |
| 182 | Cardiovascular | 3 | 0 |  | 29 | 1 | 31 | 1 | 0 | 0 | 1 | Negative |
| 183 | Cardiovascular | 6 | 2 |  | 5 | 0 | 9 | 2 | 0 | 0 | 2 | Negative |
| 184 | Cardiovascular | 4 | 2 |  | 48 | 2 | 48 | 4 | 0 | 0 | 4 | Negative |
| 185 | Cardiovascular | 2 | 0 |  | 7 | 0 | 9 | 0 | 0 | 0 | 0 | Negative |
| 186 | Cardiovascular | 8 | 0 |  | 31 | 0 | 39 | 0 | 0 | 0 | 0 | Negative |
| 187 | Cardiovascular | 2 | 0 |  | 5 | 0 | 7 | 0 | 0 | 0 | 0 | Negative |
| 188 | Cardiovascular | 0 | 0 |  | 4 | 0 | 4 | 0 | 0 | 0 | 0 | Negative |
| 189 | Cardiovascular | 4 | 0 |  | 2 | 1 | 5 | 1 | 0 | 0 | 1 | Negative |
| 190 | Cardiovascular | 9 | 1 |  | 29 | 0 | 37 | 1 | 1 | 0 | 0 | Positive |
| 191 | Cardiovascular | 11 | 0 |  | 41 | 1 | 51 | 1 | 0 | 1 | 0 | Inconclusive |
| 192 | Cardiovascular | 1 | 0 |  | 3 | 0 | 4 | 0 | 0 | 0 | 0 | Negative |
| 193 | Cardiovascular | 8 | 0 |  | 3 | 0 | 11 | 0 | 0 | 0 | 0 | Negative |
| 194 | Cardiovascular | 12 | 0 |  | 3 | 0 | 15 | 0 | 0 | 0 | 0 | Negative |
| 195 | Cardiovascular | 7 | 0 |  | 3 | 0 | 10 | 0 | 0 | 0 | 0 | Negative |
| 196 | Cardiovascular | 4 | 0 |  | 3 | 0 | 7 | 0 | 0 | 0 | 0 | Negative |
| 197 | Abdominal | 1 | 0 |  | 4 | 0 | 5 | 0 | 0 | 0 | 0 | Negative |
| 198 | Abdominal | 2 | 0 |  | 2 | 0 | 4 | 0 | 0 | 0 | 0 | Negative |
| 199 | Abdominal | 8 | 0 |  | 3 | 0 | 11 | 0 | 0 | 0 | 0 | Negative |
| 200 | Abdominal | 4 | 0 |  | 11 | 2 | 13 | 2 | 0 | 0 | 2 | Negative |
| 201 | Abdominal | 6 | 0 |  | 3 | 0 | 9 | 0 | 0 | 0 | 0 | Inconclusive |
| 202 | Abdominal | 7 | 0 |  | 3 | 0 | 10 | 0 | 0 | 0 | 0 | Negative |
| 203 | Abdominal | 3 | 0 |  | 51 | 0 | 54 | 0 | 0 | 0 | 0 | Negative |
| 204 | Abdominal | 0 | 0 |  | 3 | 0 | 3 | 0 | 0 | 0 | 0 | Negative |
| 205 | Abdominal | 7 | 0 |  | 26 | 0 | 33 | 0 | 0 | 0 | 0 | Negative |
| 206 | Abdominal | 2 | 0 |  | 55 | 1 | 56 | 1 | 0 | 0 | 1 | Negative |
| 207 | Abdominal | 4 | 0 |  | 4 | 0 | 8 | 0 | 0 | 0 | 0 | Negative |
| 208 | Abdominal | 6 | 0 |  | 5 | 0 | 11 | 0 | 0 | 0 | 0 | Negative |
| 209 | Abdominal | 3 | 0 |  | 6 | 0 | 9 | 0 | 0 | 0 | 0 | Negative |
| 210 | Abdominal | 5 | 0 |  | 4 | 0 | 9 | 0 | 0 | 0 | 0 | Negative |
| 211 | Abdominal | 8 | 2 |  | 55 | 1 | 60 | 3 | 0 | 0 | 3 | Negative |
| 212 | Abdominal | 3 | 0 |  | 5 | 0 | 8 | 0 | 0 | 0 | 0 | Negative |
| 213 | Abdominal | 4 | 0 |  | 11 | 0 | 15 | 0 | 0 | 0 | 0 | Negative |
| 214 | Abdominal | 7 | 2 |  | 8 | 0 | 13 | 2 | 0 | 0 | 2 | Negative |
| 215 | Abdominal | 4 | 2 |  | 40 | 1 | 41 | 3 | 0 | 0 | 3 | Negative |
| 216 | Abdominal | 1 | 0 |  | 24 | 0 | 25 | 0 | 0 | 0 | 0 | Negative |
| 217 | Abdominal | 2 | 0 |  | 3 | 0 | 5 | 0 | 0 | 0 | 0 | Negative |
| 218 | Abdominal | 7 | 3 |  | 32 | 1 | 35 | 4 | 0 | 2 | 2 | Inconclusive |
| 219 | Abdominal | 2 | 0 |  | 27 | 2 | 27 | 2 | 0 | 0 | 2 | Negative |
| 220 | Abdominal | 2 | 0 |  | 7 | 1 | 8 | 1 | 0 | 0 | 1 | Negative |
| 221 | Abdominal | 2 | 2 |  | 63 | 1 | 62 | 3 | 0 | 0 | 3 | Negative |
| 222 | Abdominal | 8 | 2 |  | 19 | 0 | 25 | 2 | 0 | 0 | 2 | Negative |
| 223 | Abdominal | 7 | 0 |  | 18 | 2 | 23 | 2 | 0 | 0 | 2 | Negative |
| 224 | Abdominal | 6 | 0 |  | 5 | 0 | 11 | 0 | 0 | 0 | 0 | Negative |
| 225 | Abdominal | 8 | 0 |  | 3 | 0 | 11 | 0 | 0 | 0 | 0 | Negative |
| 226 | Abdominal | 12 | 0 |  | 4 | 0 | 16 | 0 | 0 | 0 | 0 | Negative |
| 227 | Abdominal | 6 | 0 |  | 5 | 0 | 11 | 0 | 0 | 0 | 0 | Negative |
| 228 | Abdominal | 6 | 0 |  | 4 | 0 | 10 | 0 | 0 | 0 | 0 | Negative |
| 229 | Abdominal | 6 | 4 |  | 53 | 0 | 55 | 4 | 0 | 0 | 4 | Negative |
| 230 | Abdominal | 2 | 0 |  | 49 | 3 | 48 | 3 | 0 | 0 | 3 | Negative |
| 231 | Abdominal | 2 | 0 |  | 49 | 2 | 49 | 2 | 0 | 0 | 2 | Negative |
| 232 | Abdominal | 11 | 1 |  | 37 | 2 | 45 | 3 | 1 | 0 | 2 | Positive |
| 233 | Abdominal | 2 | 0 |  | 6 | 0 | 8 | 0 | 0 | 0 | 0 | Negative |
| 234 | Abdominal | 1 | 0 |  | 71 | 2 | 70 | 2 | 0 | 0 | 2 | Negative |
| 235 | Abdominal | 2 | 0 |  | 11 | 0 | 13 | 0 | 0 | 0 | 0 | Negative |
| 236 | Abdominal | 6 | 0 |  | 63 | 2 | 67 | 2 | 0 | 0 | 2 | Negative |
| 237 | Abdominal | 0 | 0 |  | 32 | 3 | 29 | 3 | 0 | 0 | 3 | Negative |
| 238 | Abdominal | 4 | 0 |  | 21 | 1 | 24 | 1 | 0 | 0 | 1 | Negative |
| 239 | Abdominal | 6 | 4 |  | 56 | 1 | 57 | 5 | 0 | 0 | 5 | Negative |
| 240 | Abdominal | 10 | 0 |  | 48 | 3 | 55 | 3 | 0 | 0 | 3 | Negative |
| 241 | Abdominal | 2 | 0 |  | 26 | 1 | 27 | 1 | 0 | 0 | 1 | Negative |
| 242 | Abdominal | 6 | 0 |  | 67 | 3 | 70 | 3 | 0 | 0 | 3 | Negative |
| 243 | Abdominal | 5 | 0 |  | 11 | 0 | 16 | 0 | 0 | 0 | 0 | Negative |
| 244 | Abdominal | 2 | 0 |  | 7 | 0 | 9 | 0 | 0 | 0 | 0 | Negative |
| 245 | Abdominal | 8 | 2 |  | 50 | 0 | 56 | 2 | 0 | 2 | 0 | Inconclusive |
| 246 | Abdominal | 6 | 1 |  | 38 | 0 | 43 | 1 | 1 | 0 | 0 | Positive |
| 247 | Urogenital | 2 | 0 |  | 4 | 0 | 6 | 0 | 0 | 0 | 0 | Negative |
| 248 | Urogenital | 6 | 4 |  | 55 | 1 | 56 | 5 | 0 | 2 | 3 | Inconclusive |
| 249 | Urogenital | 5 | 2 |  | 11 | 0 | 14 | 2 | 2 | 0 | 0 | Positive |
| 250 | Urogenital | 11 | 3 |  | 15 | 0 | 23 | 3 | 0 | 1 | 2 | Inconclusive |
| 251 | Urogenital | 2 | 0 |  | 46 | 0 | 48 | 0 | 0 | 0 | 0 | Negative |
| 252 | Urogenital | 6 | 0 |  | 5 | 0 | 11 | 0 | 0 | 0 | 0 | Negative |
| 253 | Urogenital | 2 | 0 |  | 66 | 2 | 66 | 2 | 0 | 1 | 1 | Inconclusive |
| 254 | Urogenital | 7 | 1 |  | 29 | 0 | 35 | 1 | 1 | 0 | 0 | Positive |
| 255 | Urogenital | 3 | 0 |  | 8 | 1 | 10 | 1 | 0 | 0 | 1 | Negative |
| 256 | Urogenital | 8 | 1 |  | 74 | 3 | 78 | 4 | 0 | 1 | 3 | Inconclusive |
| 257 | Urogenital | 0 | 0 |  | 3 | 0 | 3 | 0 | 0 | 0 | 0 | Negative |
| 258 | Urogenital | 2 | 0 |  | 35 | 1 | 36 | 1 | 0 | 0 | 1 | Negative |
| 259 | Urogenital | 8 | 2 |  | 37 | 0 | 43 | 2 | 0 | 0 | 2 | Negative |
| 260 | Urogenital | 4 | 0 |  | 62 | 1 | 65 | 1 | 0 | 0 | 1 | Negative |
| 261 | Urogenital | 2 | 0 |  | 8 | 0 | 10 | 0 | 0 | 0 | 0 | Negative |
| 262 | Urogenital | 4 | 0 |  | 9 | 1 | 12 | 1 | 0 | 0 | 1 | Negative |
| 263 | Urogenital | 5 | 0 |  | 45 | 0 | 50 | 0 | 0 | 0 | 0 | Negative |
| 264 | Urogenital | 7 | 2 |  | 83 | 2 | 86 | 4 | 0 | 0 | 4 | Negative |
| 265 | Urogenital | 7 | 0 |  | 48 | 1 | 54 | 1 | 0 | 0 | 1 | Negative |
| 266 | Urogenital | 3 | 0 |  | 8 | 1 | 10 | 1 | 0 | 0 | 1 | Negative |
| 267 | Urogenital | 6 | 2 |  | 11 | 0 | 15 | 2 | 0 | 2 | 0 | Inconclusive |
| 268 | Urogenital | 1 | 1 |  | 14 | 0 | 14 | 1 | 0 | 1 | 0 | Inconclusive |
| 269 | Urogenital | 4 | 2 |  | 18 | 1 | 19 | 3 | 0 | 0 | 3 | Negative |
| 270 | Urogenital | 5 | 0 |  | 37 | 0 | 42 | 0 | 0 | 0 | 0 | Negative |
| 271 | Urogenital | 7 | 0 |  | 5 | 1 | 11 | 1 | 1 | 0 | 0 | Positive |
| 272 | Urogenital | 3 | 0 |  | 13 | 0 | 16 | 0 | 0 | 0 | 0 | Negative |
| 273 | Urogenital | 5 | 0 |  | 16 | 1 | 20 | 1 | 1 | 0 | 0 | Positive |
| 274 | Urogenital | 4 | 0 |  | 8 | 1 | 11 | 1 | 0 | 0 | 1 | Negative |
| 275 | Urogenital | 2 | 0 |  | 28 | 0 | 30 | 0 | 0 | 0 | 0 | Positive |
| 276 | Urogenital | 3 | 0 |  | 11 | 0 | 14 | 0 | 0 | 0 | 0 | Negative |
| 277 | Urogenital | 5 | 0 |  | 3 | 0 | 8 | 0 | 0 | 0 | 0 | Negative |
| 278 | Urogenital | 4 | 0 |  | 8 | 0 | 12 | 0 | 0 | 0 | 0 | Negative |
| 279 | Urogenital | 4 | 0 |  | 31 | 1 | 34 | 1 | 0 | 0 | 1 | Negative |
| 280 | Urogenital | 6 | 2 |  | 55 | 2 | 57 | 4 | 0 | 0 | 4 | Negative |
| 281 | Urogenital | 6 | 0 |  | 8 | 0 | 14 | 0 | 0 | 0 | 0 | Negative |
| 282 | Urogenital | 4 | 2 |  | 10 | 0 | 12 | 2 | 2 | 0 | 0 | Positive |
| 283 | Urogenital | 4 | 0 |  | 11 | 0 | 15 | 0 | 0 | 0 | 0 | Negative |
| 284 | Urogenital | 6 | 2 |  | 19 | 0 | 23 | 2 | 0 | 0 | 2 | Negative |
| 285 | Urogenital | 8 | 0 |  | 5 | 0 | 13 | 0 | 0 | 0 | 0 | Negative |
| 286 | Urogenital | 5 | 3 |  | 23 | 2 | 23 | 5 | 2 | 0 | 3 | Positive |
| 287 | Urogenital | 4 | 0 |  | 11 | 0 | 15 | 0 | 0 | 0 | 0 | Negative |
| 288 | Urogenital | 7 | 1 |  | 65 | 2 | 69 | 3 | 0 | 0 | 3 | Negative |
| 289 | Urogenital | 8 | 0 |  | 19 | 1 | 26 | 1 | 0 | 0 | 1 | Negative |
| 290 | Urogenital | 4 | 0 |  | 5 | 1 | 8 | 1 | 0 | 1 | 0 | Inconclusive |
| 291 | Urogenital | 6 | 0 |  | 2 | 0 | 8 | 0 | 0 | 0 | 0 | Negative |
| 292 | Urogenital | 10 | 0 |  | 4 | 1 | 13 | 1 | 1 | 0 | 0 | Positive |
| 293 | Urogenital | 4 | 0 |  | 3 | 0 | 7 | 0 | 0 | 0 | 0 | Negative |
| 294 | Urogenital | 0 | 0 |  | 22 | 1 | 21 | 1 | 0 | 0 | 1 | Negative |
| 295 | Urogenital | 7 | 4 |  | 22 | 0 | 25 | 4 | 2 | 0 | 2 | Positive |
| 296 | Urogenital | 10 | 0 |  | 8 | 1 | 17 | 1 | 1 | 0 | 0 | Positive |
| 297 | Skeletal | 4 | 2 |  | 45 | 1 | 46 | 3 | 0 | 2 | 1 | Inconclusive |
| 298 | Skeletal | 2 | 0 |  | 2 | 0 | 4 | 0 | 0 | 0 | 0 | Negative |
| 299 | Skeletal | 2 | 0 |  | 100 | 0 | 102 | 0 | 0 | 0 | 0 | Negative |
| 300 | Skeletal | 7 | 0 |  | 73 | 0 | 80 | 0 | 0 | 0 | 0 | Negative |
| 301 | Skeletal | 4 | 0 |  | 51 | 3 | 52 | 3 | 0 | 0 | 3 | Negative |
| 302 | Skeletal | 5 | 2 |  | 44 | 1 | 46 | 3 | 2 | 0 | 1 | Positive |
| 303 | Skeletal | 2 | 2 |  | 12 | 0 | 12 | 2 | 2 | 0 | 0 | Positive |
| 304 | Skeletal | 2 | 0 |  | 61 | 0 | 63 | 0 | 0 | 0 | 0 | Negative |
| 305 | Skeletal | 0 | 0 |  | 72 | 3 | 69 | 3 | 0 | 0 | 3 | Negative |
| 306 | Skeletal | 2 | 0 |  | 38 | 2 | 38 | 2 | 1 | 0 | 1 | Positive |
| 307 | Skeletal | 3 | 1 |  | 38 | 0 | 40 | 1 | 1 | 0 | 0 | Negative |
| 308 | Skeletal | 3 | 1 |  | 8 | 0 | 10 | 1 | 1 | 0 | 0 | Positive |
| 309 | Skeletal | 5 | 1 |  | 12 | 0 | 16 | 1 | 1 | 0 | 0 | Positive |
| 310 | Skeletal | 9 | 1 |  | 30 | 2 | 36 | 3 | 0 | 1 | 2 | Inconclusive |
| 311 | Skeletal | 5 | 3 |  | 42 | 1 | 43 | 4 | 0 | 1 | 3 | Inconclusive |
| 312 | Skeletal | 10 | 0 |  | 15 | 0 | 25 | 0 | 0 | 0 | 0 | Negative |
| 313 | Skeletal | 0 | 0 |  | 51 | 2 | 49 | 2 | 0 | 0 | 2 | Negative |
| 314 | Skeletal | 4 | 2 |  | 57 | 2 | 57 | 4 | 0 | 0 | 4 | Negative |
| 315 | Skeletal | 6 | 0 |  | 47 | 1 | 52 | 1 | 0 | 0 | 1 | Negative |
| 316 | Skeletal | 2 | 1 |  | 39 | 1 | 39 | 2 | 1 | 0 | 1 | Positive |
| 317 | Skeletal | 6 | 4 |  | 79 | 1 | 80 | 5 | 0 | 2 | 3 | Inconclusive |
| 318 | Skeletal | 4 | 0 |  | 85 | 5 | 84 | 5 | 1 | 0 | 4 | Positive |
| 319 | Skeletal | 2 | 2 |  | 56 | 2 | 54 | 4 | 2 | 0 | 2 | Positive |
| 320 | Skeletal | 4 | 1 |  | 35 | 0 | 38 | 1 | 1 | 0 | 0 | Positive |
| 321 | Skeletal | 2 | 0 |  | 40 | 1 | 41 | 1 | 0 | 0 | 1 | Negative |
| 322 | Skeletal | 0 | 0 |  | 71 | 1 | 70 | 1 | 0 | 0 | 1 | Negative |
| 323 | Skeletal | 8 | 4 |  | 49 | 1 | 52 | 5 | 2 | 0 | 3 | Positive |
| 324 | Skeletal | 3 | 0 |  | 45 | 2 | 46 | 2 | 0 | 0 | 2 | Negative |
| 325 | Skeletal | 5 | 1 |  | 72 | 2 | 74 | 3 | 1 | 0 | 2 | Positive |
| 326 | Skeletal | 11 | 1 |  | 75 | 4 | 81 | 5 | 1 | 0 | 4 | Positive |
| 327 | Skeletal | 6 | 0 |  | 42 | 3 | 45 | 3 | 1 | 0 | 2 | Positive |
| 328 | Skeletal | 3 | 3 |  | 59 | 0 | 59 | 3 | 1 | 0 | 2 | Positive |
| 329 | Skeletal | 5 | 1 |  | 32 | 3 | 33 | 4 | 0 | 0 | 4 | Negative |
| 330 | Skeletal | 1 | 1 |  | 56 | 0 | 56 | 1 | 0 | 0 | 1 | Negative |
| 331 | Skeletal | 4 | 0 |  | 28 | 0 | 32 | 0 | 0 | 0 | 0 | Negative |
| 332 | Skeletal | 3 | 1 |  | 38 | 0 | 40 | 1 | 0 | 1 | 0 | Inconclusive |
| 333 | Skeletal | 6 | 2 |  | 42 | 1 | 45 | 3 | 0 | 0 | 3 | Negative |
| 334 | Skeletal | 8 | 4 |  | 94 | 2 | 96 | 6 | 0 | 0 | 6 | Negative |
| 335 | Skeletal | 2 | 0 |  | 47 | 0 | 49 | 0 | 0 | 0 | 0 | Negative |
| 336 | Skeletal | 0 | 0 |  | 11 | 3 | 8 | 3 | 0 | 1 | 2 | Inconclusive |
| 337 | Skeletal | 8 | 2 |  | 85 | 1 | 90 | 3 | 0 | 0 | 3 | Negative |
| 338 | Skeletal | 5 | 0 |  | 17 | 1 | 21 | 1 | 0 | 0 | 1 | Negative |
| 339 | Skeletal | 10 | 5 |  | 27 | 0 | 32 | 5 | 1 | 0 | 4 | Positive |
| 340 | Skeletal | 2 | 0 |  | 64 | 1 | 65 | 1 | 0 | 0 | 1 | Negative |
| 341 | Skeletal | 10 | 2 |  | 19 | 1 | 26 | 3 | 0 | 0 | 3 | Negative |
| 342 | Skeletal | 4 | 2 |  | 71 | 1 | 72 | 3 | 0 | 0 | 3 | Negative |
| 343 | Skeletal | 4 | 0 |  | 20 | 0 | 24 | 0 | 0 | 0 | 0 | Negative |
| 344 | Skeletal | 8 | 2 |  | 55 | 1 | 60 | 3 | 0 | 0 | 3 | Negative |
| 345 | Skeletal | 6 | 2 |  | 70 | 1 | 73 | 3 | 0 | 0 | 3 | Negative |
| 346 | Skeletal | 3 | 0 |  | 31 | 0 | 34 | 0 | 0 | 0 | 0 | Negative |
| 347 | FGR | 6 | 3 |  | 83 | 1 | 85 | 4 | 0 | 0 | 4 | Negative |
| 348 | FGR | 4 | 0 |  | 6 | 1 | 9 | 1 | 0 | 0 | 1 | Negative |
| 349 | FGR | 0 | 0 |  | 8 | 1 | 7 | 1 | 0 | 0 | 1 | Negative |
| 350 | FGR | 2 | 0 |  | 6 | 0 | 8 | 0 | 0 | 0 | 0 | Negative |
| 351 | FGR | 2 | 0 |  | 39 | 0 | 41 | 0 | 0 | 0 | 0 | Negative |
| 352 | FGR | 6 | 0 |  | 8 | 1 | 13 | 1 | 0 | 0 | 1 | Negative |
| 353 | FGR | 6 | 0 |  | 14 | 1 | 19 | 1 | 0 | 0 | 1 | Negative |
| 354 | FGR | 7 | 2 |  | 29 | 0 | 34 | 2 | 0 | 2 | 0 | Inconclusive |
| 355 | FGR | 4 | 0 |  | 15 | 0 | 19 | 0 | 0 | 0 | 0 | Negative |
| 356 | FGR | 2 | 0 |  | 37 | 1 | 38 | 1 | 0 | 0 | 1 | Negative |
| 357 | FGR | 10 | 0 |  | 9 | 0 | 19 | 0 | 0 | 0 | 0 | Negative |
| 358 | FGR | 7 | 0 |  | 9 | 0 | 16 | 0 | 0 | 0 | 0 | Negative |
| 359 | FGR | 0 | 0 |  | 15 | 0 | 15 | 0 | 0 | 0 | 0 | Negative |
| 360 | FGR | 4 | 0 |  | 6 | 0 | 10 | 0 | 0 | 0 | 0 | Negative |
| 361 | FGR | 1 | 0 |  | 4 | 0 | 5 | 0 | 0 | 0 | 0 | Negative |
| 362 | FGR | 1 | 1 |  | 18 | 0 | 18 | 1 | 0 | 1 | 0 | Inconclusive |
| 363 | FGR | 4 | 0 |  | 4 | 0 | 8 | 0 | 0 | 0 | 0 | Negative |
| 364 | FGR | 2 | 0 |  | 46 | 0 | 48 | 0 | 0 | 0 | 0 | Inconclusive |
| 365 | FGR | 3 | 0 |  | 59 | 1 | 61 | 1 | 0 | 0 | 1 | Negative |
| 366 | FGR | 1 | 1 |  | 72 | 2 | 70 | 3 | 1 | 0 | 2 | Positive |
| 367 | FGR | 4 | 2 |  | 45 | 1 | 46 | 3 | 0 | 0 | 3 | Negative |
| 368 | FGR | 3 | 0 |  | 13 | 0 | 16 | 0 | 0 | 0 | 0 | Negative |
| 369 | FGR | 4 | 2 |  | 32 | 0 | 34 | 2 | 0 | 0 | 2 | Negative |
| 370 | FGR | 0 | 0 |  | 74 | 2 | 72 | 2 | 0 | 0 | 2 | Negative |
| 371 | FGR | 0 | 0 |  | 37 | 2 | 35 | 2 | 0 | 0 | 2 | Negative |
| 372 | FGR | 2 | 0 |  | 20 | 1 | 21 | 1 | 0 | 0 | 1 | Negative |
| 373 | FGR | 2 | 0 |  | 26 | 0 | 28 | 0 | 0 | 0 | 0 | Negative |
| 374 | FGR | 6 | 2 |  | 31 | 1 | 34 | 3 | 0 | 0 | 3 | Negative |
| 375 | FGR | 3 | 0 |  | 32 | 1 | 34 | 1 | 0 | 0 | 1 | Negative |
| 376 | FGR | 4 | 0 |  | 48 | 1 | 51 | 1 | 0 | 0 | 1 | Negative |
| 377 | FGR | 7 | 0 |  | 44 | 0 | 51 | 0 | 0 | 0 | 0 | Negative |
| 378 | FGR | 6 | 3 |  | 68 | 1 | 70 | 4 | 0 | 0 | 4 | Negative |
| 379 | FGR | 4 | 2 |  | 32 | 1 | 33 | 3 | 0 | 0 | 3 | Negative |
| 380 | FGR | 6 | 0 |  | 33 | 1 | 38 | 1 | 0 | 0 | 1 | Negative |
| 381 | FGR | 5 | 0 |  | 46 | 3 | 48 | 3 | 0 | 0 | 3 | Negative |
| 382 | FGR | 7 | 1 |  | 90 | 0 | 96 | 1 | 1 | 0 | 0 | Positive |
| 383 | FGR | 10 | 1 |  | 13 | 0 | 22 | 1 | 0 | 1 | 0 | Inconclusive |
| 384 | FGR | 5 | 0 |  | 8 | 0 | 13 | 0 | 0 | 0 | 0 | Negative |
| 385 | FGR | 5 | 2 |  | 35 | 3 | 35 | 5 | 0 | 0 | 5 | Negative |
| 386 | FGR | 5 | 0 |  | 21 | 0 | 26 | 0 | 0 | 0 | 0 | Negative |
| 387 | FGR | 4 | 2 |  | 35 | 2 | 35 | 4 | 0 | 2 | 2 | Inconclusive |
| 388 | FGR | 2 | 0 |  | 27 | 1 | 28 | 1 | 0 | 0 | 1 | Negative |
| 389 | FGR | 4 | 2 |  | 70 | 0 | 72 | 2 | 0 | 0 | 2 | Negative |
| 390 | FGR | 6 | 0 |  | 41 | 1 | 46 | 1 | 0 | 0 | 1 | Negative |
| 391 | FGR | 8 | 4 |  | 52 | 3 | 53 | 7 | 0 | 0 | 7 | Negative |
| 392 | FGR | 6 | 0 |  | 31 | 0 | 37 | 0 | 0 | 0 | 0 | Negative |
| 393 | FGR | 0 | 0 |  | 35 | 0 | 35 | 0 | 0 | 0 | 0 | Negative |
| 394 | FGR | 8 | 0 |  | 42 | 3 | 47 | 3 | 0 | 0 | 3 | Negative |
| 395 | FGR | 6 | 2 |  | 25 | 0 | 29 | 2 | 0 | 0 | 2 | Negative |
| 396 | FGR | 0 | 0 |  | 31 | 1 | 30 | 1 | 0 | 0 | 1 | Negative |
| 397 | Hydrops | 10 | 2 |  | 12 | 0 | 20 | 2 | 0 | 0 | 2 | Negative |
| 398 | Hydrops | 8 | 0 |  | 4 | 0 | 12 | 0 | 0 | 0 | 0 | Negative |
| 399 | Hydrops | 1 | 1 |  | 4 | 2 | 2 | 3 | 0 | 0 | 3 | Negative |
| 400 | Hydrops | 9 | 0 |  | 8 | 0 | 17 | 0 | 0 | 0 | 0 | Negative |
| 401 | Hydrops | 2 | 0 |  | 7 | 0 | 9 | 0 | 0 | 0 | 0 | Negative |
| 402 | Hydrops | 7 | 0 |  | 5 | 0 | 12 | 0 | 0 | 0 | 0 | Negative |
| 403 | Hydrops | 4 | 0 |  | 5 | 0 | 9 | 0 | 0 | 0 | 0 | Negative |
| 404 | Hydrops | 8 | 0 |  | 11 | 0 | 19 | 0 | 0 | 0 | 0 | Inconclusive |
| 405 | Hydrops | 4 | 0 |  | 4 | 0 | 8 | 0 | 0 | 0 | 0 | Negative |
| 406 | Hydrops | 6 | 2 |  | 6 | 0 | 10 | 2 | 0 | 0 | 2 | Negative |
| 407 | Hydrops | 8 | 0 |  | 5 | 0 | 13 | 0 | 0 | 0 | 0 | Negative |
| 408 | Hydrops | 6 | 0 |  | 6 | 0 | 12 | 0 | 0 | 0 | 0 | Negative |
| 409 | Hydrops | 4 | 1 |  | 36 | 0 | 39 | 1 | 0 | 1 | 0 | Inconclusive |
| 410 | Hydrops | 7 | 0 |  | 24 | 0 | 31 | 0 | 0 | 0 | 0 | Negative |
| 411 | Hydrops | 0 | 0 |  | 29 | 1 | 28 | 1 | 0 | 1 | 0 | Inconclusive |
| 412 | Hydrops | 11 | 0 |  | 18 | 0 | 29 | 0 | 0 | 0 | 0 | Negative |
| 413 | Hydrops | 9 | 0 |  | 18 | 1 | 26 | 1 | 0 | 0 | 1 | Negative |
| 414 | Hydrops | 2 | 0 |  | 47 | 0 | 49 | 0 | 0 | 0 | 0 | Negative |
| 415 | Hydrops | 7 | 1 |  | 23 | 0 | 29 | 1 | 0 | 1 | 0 | Inconclusive |
| 416 | Hydrops | 4 | 1 |  | 24 | 1 | 26 | 2 | 1 | 0 | 1 | Positive |
| 417 | Hydrops | 4 | 0 |  | 38 | 0 | 42 | 0 | 0 | 0 | 0 | Negative |
| 418 | Hydrops | 10 | 6 |  | 63 | 1 | 66 | 7 | 0 | 2 | 5 | Inconclusive |
| 419 | Hydrops | 0 | 0 |  | 46 | 2 | 44 | 2 | 0 | 0 | 2 | Negative |
| 420 | Hydrops | 6 | 2 |  | 25 | 1 | 28 | 3 | 0 | 0 | 3 | Negative |
| 421 | Hydrops | 4 | 0 |  | 19 | 0 | 23 | 0 | 0 | 0 | 0 | Negative |
| 422 | Hydrops | 6 | 4 |  | 54 | 0 | 56 | 4 | 0 | 2 | 2 | Inconclusive |
| 423 | Hydrops | 4 | 0 |  | 18 | 0 | 22 | 0 | 0 | 0 | 0 | Negative |
| 424 | Hydrops | 4 | 2 |  | 22 | 0 | 24 | 2 | 2 | 0 | 0 | Positive |
| 425 | Hydrops | 2 | 0 |  | 17 | 1 | 18 | 1 | 0 | 1 | 0 | Inconclusive |
| 426 | Increased NT and cystic hygroma | 6 | 1 |  | 13 | 1 | 17 | 2 | 0 | 0 | 2 | Negative |
| 427 | Increased NT and cystic hygroma | 4 | 0 |  | 15 | 0 | 19 | 0 | 0 | 0 | 0 | Negative |
| 428 | Increased NT and cystic hygroma | 5 | 1 |  | 15 | 0 | 19 | 1 | 1 | 0 | 0 | Positive |
| 429 | Increased NT and cystic hygroma | 7 | 1 |  | 12 | 1 | 17 | 2 | 0 | 1 | 1 | Inconclusive |
| 430 | Increased NT and cystic hygroma | 3 | 0 |  | 31 | 1 | 33 | 1 | 0 | 0 | 1 | Negative |
| 431 | Increased NT and cystic hygroma | 5 | 1 |  | 7 | 0 | 11 | 1 | 1 | 0 | 0 | Positive |
| 432 | Increased NT and cystic hygroma | 4 | 0 |  | 25 | 0 | 29 | 0 | 0 | 0 | 0 | Negative |
| 433 | Increased NT and cystic hygroma | 4 | 0 |  | 8 | 1 | 11 | 1 | 0 | 0 | 1 | Negative |
| 434 | Increased NT and cystic hygroma | 3 | 2 |  | 10 | 1 | 10 | 3 | 0 | 0 | 3 | Negative |
| 435 | Increased NT and cystic hygroma | 5 | 0 |  | 9 | 0 | 14 | 0 | 0 | 0 | 0 | Negative |
| 436 | Increased NT and cystic hygroma | 0 | 0 |  | 10 | 0 | 10 | 0 | 0 | 0 | 0 | Negative |
| 437 | Increased NT and cystic hygroma | 3 | 2 |  | 7 | 0 | 8 | 2 | 0 | 0 | 2 | Negative |
| 438 | Increased NT and cystic hygroma | 8 | 2 |  | 8 | 0 | 14 | 2 | 1 | 0 | 1 | Positive |
| 439 | Increased NT and cystic hygroma | 10 | 0 |  | 9 | 0 | 19 | 0 | 0 | 0 | 0 | Negative |
| 440 | Increased NT and cystic hygroma | 0 | 0 |  | 10 | 0 | 10 | 0 | 0 | 0 | 0 | Negative |
| 441 | Increased NT and cystic hygroma | 4 | 0 |  | 8 | 1 | 11 | 1 | 0 | 0 | 1 | Negative |
| 442 | Increased NT and cystic hygroma | 1 | 0 |  | 16 | 3 | 14 | 3 | 0 | 0 | 3 | Negative |
| 443 | Increased NT and cystic hygroma | 9 | 0 |  | 11 | 0 | 20 | 0 | 0 | 0 | 0 | Negative |
| 444 | Increased NT and cystic hygroma | 2 | 0 |  | 33 | 1 | 34 | 1 | 0 | 0 | 1 | Negative |
| 445 | Increased NT and cystic hygroma | 8 | 0 |  | 23 | 0 | 31 | 0 | 0 | 0 | 0 | Negative |
| 446 | Increased NT and cystic hygroma | 2 | 0 |  | 8 | 0 | 10 | 0 | 0 | 0 | 0 | Negative |
| 447 | Increased NT and cystic hygroma | 3 | 0 |  | 49 | 0 | 52 | 0 | 0 | 0 | 0 | Negative |
| 448 | Increased NT and cystic hygroma | 1 | 1 |  | 37 | 2 | 35 | 3 | 0 | 1 | 2 | Inconclusive |
| 449 | Increased NT and cystic hygroma | 8 | 4 |  | 44 | 1 | 47 | 5 | 0 | 0 | 5 | Negative |
| 450 | Increased NT and cystic hygroma | 5 | 0 |  | 56 | 2 | 59 | 2 | 0 | 0 | 2 | Negative |
| 451 | Increased NT and cystic hygroma | 5 | 2 |  | 35 | 1 | 37 | 3 | 1 | 0 | 2 | Positive |
| 452 | Increased NT and cystic hygroma | 11 | 0 |  | 43 | 0 | 54 | 0 | 0 | 0 | 0 | Negative |
| 453 | Increased NT and cystic hygroma | 6 | 0 |  | 14 | 0 | 20 | 0 | 0 | 0 | 0 | Negative |
| 454 | Increased NT and cystic hygroma | 7 | 0 |  | 56 | 1 | 62 | 1 | 0 | 0 | 1 | Negative |
| 455 | Increased NT and cystic hygroma | 1 | 1 |  | 36 | 0 | 36 | 1 | 1 | 0 | 0 | Positive |
| 456 | Increased NT and cystic hygroma | 4 | 2 |  | 25 | 1 | 26 | 3 | 0 | 0 | 3 | Negative |
| 457 | Increased NT and cystic hygroma | 0 | 0 |  | 70 | 3 | 67 | 3 | 0 | 0 | 3 | Negative |
| 458 | Increased NT and cystic hygroma | 0 | 0 |  | 81 | 3 | 78 | 3 | 0 | 0 | 3 | Negative |
| 459 | Increased NT and cystic hygroma | 4 | 0 |  | 58 | 1 | 61 | 1 | 0 | 0 | 1 | Negative |
| 460 | Increased NT and cystic hygroma | 1 | 1 |  | 48 | 0 | 48 | 1 | 1 | 0 | 0 | Positive |
| 461 | Increased NT and cystic hygroma | 12 | 2 |  | 8 | 0 | 18 | 2 | 0 | 0 | 2 | Negative |
| 462 | Increased NT and cystic hygroma | 6 | 0 |  | 42 | 1 | 47 | 1 | 0 | 0 | 1 | Negative |
| 463 | Increased NT and cystic hygroma | 4 | 2 |  | 61 | 1 | 62 | 3 | 0 | 0 | 3 | Negative |
| 464 | Increased NT and cystic hygroma | 2 | 0 |  | 47 | 0 | 49 | 0 | 0 | 0 | 0 | Negative |
| 465 | Increased NT and cystic hygroma | 8 | 0 |  | 16 | 1 | 23 | 1 | 0 | 0 | 1 | Negative |
| 466 | Increased NT and cystic hygroma | 9 | 0 |  | 26 | 1 | 34 | 1 | 0 | 0 | 1 | Negative |
| 467 | Increased NT and cystic hygroma | 5 | 0 |  | 28 | 0 | 33 | 0 | 0 | 0 | 0 | Negative |
| 468 | Increased NT and cystic hygroma | 6 | 2 |  | 22 | 0 | 26 | 2 | 0 | 0 | 2 | Negative |
| 469 | Increased NT and cystic hygroma | 6 | 0 |  | 26 | 0 | 32 | 0 | 0 | 0 | 0 | Negative |
| 470 | Increased NT and cystic hygroma | 5 | 2 |  | 16 | 0 | 19 | 2 | 0 | 0 | 2 | Negative |
| 471 | Increased NT and cystic hygroma | 6 | 2 |  | 51 | 2 | 53 | 4 | 0 | 2 | 2 | Inconclusive |
| 472 | Increased NT and cystic hygroma | 8 | 2 |  | 14 | 1 | 19 | 3 | 0 | 0 | 3 | Negative |
| 473 | Increased NT and cystic hygroma | 3 | 0 |  | 27 | 2 | 28 | 2 | 0 | 0 | 2 | Negative |
| 474 | Increased NT and cystic hygroma | 2 | 0 |  | 19 | 2 | 19 | 2 | 0 | 0 | 2 | Negative |
| 475 | Increased NT and cystic hygroma | 0 | 0 |  | 19 | 1 | 18 | 1 | 0 | 0 | 1 | Negative |
| 476 | Multisystem | 5 | 2 |  | 52 | 0 | 55 | 2 | 0 | 2 | 0 | Inconclusive |
| 477 | Multisystem | 2 | 0 |  | 33 | 0 | 35 | 0 | 0 | 0 | 0 | Negative |
| 478 | Multisystem | 6 | 0 |  | 34 | 1 | 39 | 1 | 1 | 0 | 0 | Positive |
| 479 | Multisystem | 4 | 0 |  | 69 | 1 | 72 | 1 | 0 | 0 | 1 | Negative |
| 480 | Multisystem | 11 | 4 |  | 65 | 1 | 71 | 5 | 2 | 0 | 3 | Positive |
| 481 | Multisystem | 6 | 1 |  | 42 | 0 | 47 | 1 | 0 | 1 | 0 | Inconclusive |
| 482 | Multisystem | 4 | 2 |  | 41 | 1 | 42 | 3 | 0 | 0 | 3 | Negative |
| 483 | Multisystem | 8 | 4 |  | 46 | 1 | 49 | 5 | 2 | 0 | 3 | Positive |
| 484 | Multisystem | 5 | 4 |  | 53 | 2 | 52 | 6 | 0 | 0 | 6 | Negative |
| 485 | Multisystem | 6 | 4 |  | 67 | 0 | 69 | 4 | 2 | 0 | 2 | Positive |
| 486 | Multisystem | 5 | 3 |  | 52 | 1 | 53 | 4 | 0 | 0 | 4 | Negative |
| 487 | Multisystem | 10 | 4 |  | 72 | 3 | 75 | 7 | 2 | 2 | 3 | Positive |
| 488 | Multisystem | 4 | 0 |  | 33 | 2 | 35 | 2 | 0 | 0 | 2 | Negative |
| 489 | Multisystem | 6 | 2 |  | 64 | 1 | 67 | 3 | 0 | 0 | 3 | Negative |
| 490 | Multisystem | 4 | 2 |  | 72 | 4 | 70 | 6 | 0 | 0 | 6 | Negative |
| 491 | Multisystem | 4 | 0 |  | 27 | 0 | 31 | 0 | 0 | 0 | 0 | Negative |
| 492 | Multisystem | 5 | 0 |  | 59 | 3 | 61 | 3 | 0 | 0 | 3 | Negative |
| 493 | Multisystem | 0 | 0 |  | 60 | 0 | 60 | 0 | 0 | 0 | 0 | Negative |
| 494 | Multisystem | 5 | 1 |  | 28 | 0 | 32 | 1 | 1 | 0 | 0 | Positive |
| 495 | Multisystem | 0 | 0 |  | 87 | 1 | 86 | 1 | 0 | 0 | 1 | Negative |
| 496 | Multisystem | 9 | 2 |  | 44 | 2 | 49 | 4 | 1 | 0 | 3 | Positive |
| 497 | Multisystem | 4 | 2 |  | 70 | 2 | 70 | 4 | 0 | 2 | 2 | Inconclusive |
| 498 | Multisystem | 9 | 2 |  | 47 | 1 | 53 | 3 | 0 | 0 | 3 | Negative |
| 499 | Multisystem | 0 | 0 |  | 59 | 1 | 58 | 1 | 0 | 0 | 1 | Negative |
| 500 | Multisystem | 3 | 1 |  | 49 | 1 | 50 | 2 | 0 | 1 | 1 | Inconclusive |
| 501 | Multisystem | 6 | 1 |  | 74 | 4 | 75 | 5 | 1 | 0 | 4 | Positive |
| 502 | Multisystem | 6 | 5 |  | 52 | 0 | 53 | 5 | 0 | 1 | 4 | Inconclusive |
| 503 | Multisystem | 8 | 2 |  | 53 | 1 | 58 | 3 | 0 | 0 | 3 | Negative |
| 504 | Multisystem | 5 | 2 |  | 14 | 0 | 17 | 2 | 0 | 2 | 0 | Inconclusive |
| 505 | Multisystem | 2 | 2 |  | 48 | 2 | 46 | 4 | 1 | 0 | 3 | Positive |
| 506 | Multisystem | 6 | 2 |  | 29 | 0 | 33 | 2 | 0 | 2 | 0 | Inconclusive |
| 507 | Multisystem | 7 | 3 |  | 45 | 2 | 47 | 5 | 2 | 1 | 2 | Positive |
| 508 | Multisystem | 5 | 1 |  | 19 | 0 | 23 | 1 | 1 | 0 | 0 | Positive |
| 509 | Multisystem | 4 | 4 |  | 49 | 0 | 49 | 4 | 1 | 0 | 3 | Positive |
| 510 | Multisystem | 6 | 2 |  | 50 | 2 | 52 | 4 | 2 | 0 | 2 | Positive |
| 511 | Multisystem | 6 | 2 |  | 36 | 1 | 39 | 3 | 0 | 0 | 3 | Negative |
| 512 | Multisystem | 4 | 0 |  | 58 | 1 | 61 | 1 | 0 | 0 | 1 | Negative |
| 513 | Multisystem | 8 | 4 |  | 53 | 3 | 54 | 7 | 0 | 0 | 7 | Negative |
| 514 | Multisystem | 2 | 0 |  | 61 | 2 | 61 | 2 | 0 | 0 | 2 | Negative |
| 515 | Multisystem | 4 | 0 |  | 36 | 1 | 39 | 1 | 0 | 0 | 1 | Negative |
| 516 | Multisystem | 11 | 3 |  | 45 | 1 | 52 | 4 | 0 | 0 | 4 | Negative |
| 517 | Multisystem | 1 | 1 |  | 73 | 3 | 70 | 4 | 1 | 0 | 3 | Positive |
| 518 | Multisystem | 6 | 0 |  | 52 | 0 | 58 | 0 | 0 | 0 | 0 | Negative |
| 519 | Multisystem | 1 | 1 |  | 81 | 1 | 80 | 2 | 1 | 0 | 1 | Positive |
| 520 | Multisystem | 7 | 3 |  | 71 | 3 | 72 | 6 | 1 | 0 | 5 | Positive |
| 521 | Multisystem | 8 | 2 |  | 43 | 1 | 48 | 3 | 0 | 0 | 3 | Negative |
| 522 | Multisystem | 9 | 0 |  | 62 | 3 | 68 | 3 | 0 | 0 | 3 | Negative |
| 523 | Multisystem | 2 | 2 |  | 98 | 5 | 93 | 7 | 0 | 0 | 7 | Negative |
| 524 | Multisystem | 14 | 2 |  | 73 | 1 | 84 | 3 | 0 | 0 | 3 | Negative |
| 525 | Multisystem | 3 | 3 |  | 53 | 1 | 52 | 4 | 0 | 0 | 4 | Negative |

**Additional file 2 Table S7. Number of variants analyzed in step 1 and 2 based on malformation classification or overall result category**

| **Malformation classification** | **Genotype-driven** | |  |  | **Phenotype-driven** | | | **Total Number of variants closely Reviewed** [Mean/Median (Range)] | **Total Number of variants interpreted as irrelevant after quick review** [Mean/Median (Range)] |
| --- | --- | --- | --- | --- | --- | --- | --- | --- | --- |
|  | **Total number of variants based on origin/ zygosity/ pathogenicity** [Mean/Median (Range)] | **Number of variants for focused clinical correlation** [Mean/Median (Range)] |  | **Total number of variants based on HPO matching** [Mean/Median (Range)] | |  | **Additional number of variants closely reviewed in this step** [Mean/Median (Range)] |  |  |
| Central nervous (n=50) | 4.6 / 4 (0-13) | 1.2 / 1 (0-5) |  | 41.4 / 26 (5-151) | |  | 1 / 0.5 (0-4) | 2.2 / 2 (0-7) | 43.8 / 29 (9-149) |
| Facial (n=50) | 6.0 / 5.5 (1-15) | 1.1 / 0 (0-6) |  | 27.3 / 13.5 (3-94) | |  | 1.0 / 1 (0-4) | 2.2 / 2 (0-7) | 31.1 / 20 (4-95) |
| Chest (n=46) | 5.3 / 5 (0-18) | 0.6 / 0 (0-5) |  | 26.4 / 27 (1-72) | |  | 0.7 / 0 (0-5) | 1.2 / 1 (0-10) | 30.4 / 30 (3-73) |
| Cardiovascular (n=50) | 5.7 / 6 (0-12) | 0.6 / 0 (0-4) |  | 14.4 / 7 (2-58) | |  | 0.5 / 0 (0-4) | 1.1 / 1 (0-4) | 19.0 / 14 (3-63) |
| Abdominal (n=50) | 4.7/ 4.5 (0-12) | 0.5 / 0 (0-4) |  | 24.2 / 14.5 (2-71) | |  | 0.7 / 0 (0-3) | 1.2 / 0.5 (0-5) | 27.6 / 19.5 (3-70) |
| Urogenital (n=50) | 4.9 / 5 (0-11) | 0.7 / 0 (0-4) |  | 22.9 / 13.5 (2-83) | |  | 0.6 / 0 (0-3) | 1.3 / 1 (0-5) | 26.5 / 16.5 (3-86) |
| Skeletal (n=50) | 4.4 / 4 (0-11) | 1.1 / 1 (0-5) |  | 47.0 / 45 (2-100) | |  | 1.1 / 1 (0-5) | 2.3 / 2.5 (0-6) | 49.2 / 46 (4-102) |
| FGR (n=50) | 4.1 / 4 (0-10) | 0.6 / 0 (0-4) |  | 32.5 / 31.5 (4-90) | |  | 0.8 / 1 (0-3) | 1.4 / 1 (0-7) | 35.2 / 34 (5-96) |
| Hydrops (n=29) | 5.4/ 6 (0-11) | 0.8 / 0 (0-6) |  | 20.6 / 18 (4-63) | |  | 0.3 / 0 (0-2) | 1.1 / 0 (0-7) | 24.9 / 23 (2-66) |
| Increased NT and cystic hygroma (n=50) | 4.6 / 4.5 (0-12) | 0.7 / 0 (0-4) |  | 27.2 / 22.5 (7-81) | |  | 0.7 / 1 (0-3) | 1.4 / 1 (0-5) | 30.5 / 26 (8-78) |
| Multisystem (n=50) | 5.3 / 5 (0-14) | 1.7 / 2 (0-5) |  | 53.1 / 41 (14-98) | |  | 1.3 / 1 (0-5) | 3.1 / 3 (0-7) | 55.3 / 53 (17-93) |
| **Total** | **5.0 / 5 (0-18)** | **0.9 / 0 (0-6)** |  | **31.1 / 26 (1-151)** | |  | **0.8 / 0 (0-5)** | **1.7 / 1 (0-10)** | **34.3 / 29 (2-149)** |

| **Overall**  **Result**  **Category** | **Genotype-driven** | |  |  | **Phenotype-driven** | | | **Total Number of variants closely Reviewed** [Mean/Median (Range)] | **Total Number of variants interpreted as irrelevant after quick review** [Mean/Median (Range)] |
| --- | --- | --- | --- | --- | --- | --- | --- | --- | --- |
|  | **Total number of variants based on origin/ zygosity/ pathogenicity** [Mean/Median (Range)] | **Number of variants for focused clinical correlation** [Mean/Median (Range)] |  | **Total number of variants based on HPO matching** [Mean/Median (Range)] | |  | **Additional number of variants closely reviewed in this step** [Mean/Median (Range)] |  |  |
| Positive (n=79) | 5.4 / 5 (1-11) | 1.7 / 1 (0-5) |  | 40.6 / 38 (3-112) | |  | 1.2 / 1 (0-5) | 2.8 / 3 (0-7) | 43.2 / 39 (4-110) |
| Inconclusive (n=60) | 5.7 / 6 (0-18) | 1.9 / 2 (0-6) |  | 39.4 / 37 (3-110) | |  | 1.0 / 1 (0-5) | 2.8 / 2 (0-10) | 42.3 / 38 (8-109) |
| Negative (n=386) | 4.8 / 4 (0-15) | 0.6 / 0 (0-5) |  | 27.8 / 20 (8-151) | |  | 0.7 / 0 (0-5) | 1.3 / 1 (0-7) | 31.3 / 25 (2-149) |
| p value (ANOVA) | 0.0185 | <0.01 |  | <0.01 | |  | <0.01 | <0.01 | <0.01 |
| **Total** | **5.0 / 5 (0-18)** | **0.9 / 0 (0-6)** |  | **31.1 / 26 (1-151)** | |  | **0.8 / 0 (0-5)** | **1.7 / 1 (0-10)** | **34.3 / 29 (2-149)** |

**Additional file 2 Table S8. Fetuses with VUS results detected by ES**

| **Case ID** | **Gender** | **Ultrasound findings** | **Gene(OMIM ID)** | **Transcript** | **Nucleotide change** | **Amino acid change** | **Variant type** | **Zygosity** | **Classification** | **Origin** | **Inheritance** | **Disease(OMIM ID)** | **Pregnancy outcome** | **Group** |
| --- | --- | --- | --- | --- | --- | --- | --- | --- | --- | --- | --- | --- | --- | --- |
| **Central nervus** | | |  |  |  |  |  |  |  |  |  |  |  |  |
| **230** | female | Microcephaly | BCL11A(606557) | NM_022893.3 | c.2437A>G | p.(Ser813Gly) | missense | Het | VUS | De novo | AD | INTELLECTUAL DEVELOPMENTAL DISORDER WITH PERSISTENCE OF FETAL HEMOGLOBIN (617101) | Termination | Retrospective |
| **231** | male | Agenesis of corpus callosum,Ventriculomegaly | PTDSS1(612792) | NM_014754.2 | c.735C>A | p.(Tyr245Ter) | nonsense | Het | VUS | De novo | AD | LENZ-MAJEWSKI HYPEROSTOTIC DWARFISM(151050) | Termination | Retrospective |
| **232** | female | Microcephaly | CREBBP(600140) | NM_004380.2 | c.2393A>G | p.(Gln798Arg) | missense | Het | VUS | De novo | AD | RUBINSTEIN-TAYBI SYNDROME 1(180849); MENKE-HENNEKAM SYNDROME 1(618332) | Live birth | Retrospective |
| **233** | female | Hydrocephalus | PIEZO2(613629) | NM_022068.3 | c.7139G>T | p.(Cys2380Phe) | missense | Het | VUS | De novo | AD | MARDEN-WALKER SYNDROME(248700) | Live birth | Retrospective |
| **234** | male | Dilation of lateral ventricles | BRAF(164757) | NM_001374258.1 | c.1061G>A | p.(Arg354Gln) | missense | Het | VUS | De novo | AD | CARDIOFACIOCUTANEOUS SYNDROME 1(115150) | Live birth | Retrospective |
| **235** | male | Cerebellar vermis hypoplasia | CEP120(613446) | NM_153223.3 | c.2132_2133delAA | p.(Lys711ThrfsTer7) | frameshift | Het | LP | Mat | AR | JOUBERT SYNDROME 31(617761) | Live birth | Retrospective |
|  |  |  |  | NM_153223.3 | c.322-8T>C |  | splice_region | Het | VUS-high risk | Pat |  |  |  |  |
| **236** | female | Microcephaly | CEP135(611423) | NM_025009.4 | c.1306C>T | p.(Arg436Ter) | nonsense | Het | LP | Pat | AR | MICROCEPHALY 8, PRIMARY, AUTOSOMAL RECESSIVE(614673) | Termination | Retrospective |
|  |  |  |  | NM_025009.4 | c.3229C>T | p.(Arg1077Trp) | missense | Het | VUS-high risk | Mat |  |  |  |  |
| **237** | female | Spina bifida, Ventriculomegaly | ASXL2(612991) | NM_018263.4 | c.1586A>G | p.(Lys529Arg) | missense | Het | VUS | De novo | AD | SHASHI-PENA SYNDROME(617190) | Termination | Retrospective |
| **238** | male | Agenesis of corpus callosum | L1CAM(308840) | NM_000425.4 | c.2254G>A | p.(Val752Met) | missense | Hemi | VUS-high risk | Mat | XR | MASA SYNDROME(303350) | Termination | Prospective |
| **239** | female | Microcephaly, FGR | TBCD(604649) | NM_005993.4 | c.1573C>T | p.(Pro525Ser) | missense | Het | VUS-high risk | Mat | AR | ENCEPHALOPATHY, PROGRESSIVE, EARLY-ONSET, WITH BRAIN ATROPHY AND THIN CORPUS CALLOSUM(617193) | Live birth | Prospective |
|  |  |  |  | NM_005993.4 | c.2178+5A>G |  | splice_region | Het | VUS | Pat |  |  |  |  |
| **240** | male | Dilation of lateral ventricles, FGR | HSPG2(142461) | NM_005529 | c.11926G>A | p.(Val3976Met) | missense | Het | VUS | Mat | AR | SCHWARTZ-JAMPEL SYNDROME, TYPE 1(255800) | Live birth | Prospective |
|  |  |  |  | NM_005529 | c.4627-8G>A | - | splice_region | Het | VUS | Pat |  |  |  |  |
| **241** | male | Hypodysplasia of the corpus callosum | HERC1(605109) | NM_003922.3 | c.6110C>T | p.(Pro2037Leu) | missense | Het | VUS | Pat | AR | MACROCEPHALY, DYSMORPHIC FACIES, AND PSYCHOMOTOR RETARDATION(617011) | Termination | Prospective |
|  |  |  |  | NM_003922.3 | c.5582T>C | p.(Met1861Thr) | missense | Het | VUS | Mat |  |  |  |  |
| **242** | female | Microcephaly | FOXRED1(613622) | NM_017547.3 | c.10A>G | p.(Arg4Gly) | missense | Hom | VUS | Mat+Pat | AR | MITOCHONDRIAL COMPLEX I DEFICIENCY, NUCLEAR TYPE 19(618241) | Live birth | Prospective |
| **243** | female | Microcephaly, FGR | TUBGCP6(610053) | NM_020461.3 | c.3117C>T | p.(Tyr1039=) | synonymous | Het | VUS | Pat | AR | MICROCEPHALY AND CHORIORETINOPATHY, AUTOSOMAL RECESSIVE, 1(251270) | Termination | Prospective |
|  |  |  |  | NM_020461.3 | c.2066-6A>G |  | splice_region | Het | VUS-high risk | Mat |  |  |  |  |
| **244*** | male | Microcephaly | TUBGCP6(610053) | NM_020461.3 | c.4485-31_4485-22delGCCCGCCCTG |  | intron_variant | Het | VUS | Pat | AR | MICROCEPHALY AND CHORIORETINOPATHY, AUTOSOMAL RECESSIVE, 1(251270) | Termination | Prospective |
|  |  |  |  | NM_020461.3 | c.2066-6A>G |  | splice_region | Het | VUS-high risk | Mat |  |  |  |  |
| **245** | male | Hemivertebrae, Abnormality of the spinal cord | PTCH1(601309) | NM_000264.3 | c.2687C>T | p.(Pro896Leu) | missense | Het | VUS | Mat | AD | BASAL CELL NEVUS SYNDROM(109400) | Live birth | Prospective |
| **246*** | male | Spina bifida, Myelomeningocele | BCOR(300485) | NM_001123385.1 | c.2939T>C | p.(Val980Ala) | missense | Hemi | VUS | Mat | XD | MICROPHTHALMIA, SYNDROMIC 2(300166) | Termination | Prospective |
| **247** | male | Microcephaly, FGR | KANSL1(612452) | NM_015443.3 | c.428C>T | p.(Thr143Met) | missense | Het | VUS | De novo | AD | KOOLEN-DE VRIES SYNDROME(610443) | In Pregnancy | Prospective |
| **248** | male | Agenesis of corpus callosum | STAG2(300826) | NM_001042749.2 | c.1726G>A | p.(Ala576Thr) | missense | Hemi | VUS | Mat | XL | HOLOPROSENCEPHALY 13, X-LINKED(301043) | Termination | Prospective |
| **249** | female | Microcephaly, FGR | PRKDC(600899) | NM_006904.6 | c.11694G>A | p.(Trp3898Ter) | nonsense | Het | VUS | Mat | AR | IMMUNODEFICIENCY 26 WITH OR WITHOUT NEUROLOGIC ABNORMALITIES(615966) | Live birth | Prospective |
|  |  |  |  | NM_006904.6 | c.8043C>T | p.(Pro2681=) | synonymous | Het | VUS | Pat |  |  |  |  |
| **250** | male | Hypoplasia of the corpus callosum, Ventriculomegaly, Abnormality of the septum pellucidum | TUBB2B(612850) | NM_178012.4 | c.173A>G | p.(Lys58Arg) | missense | Het | VUS | De novo | AD | CORTICAL DYSPLASIA, COMPLEX, WITH OTHER BRAIN MALFORMATIONS 7(610031) | Termination | Prospective |
| **Facial** | | |  |  |  |  |  |  |  |  |  |  |  |  |
| **251** | male | Cleft lip,Cleft palate | CHD7(608892) | NM_017780.3 | c.5027C>T | p.(Thr1676Ile) | missense | Het | VUS | De novo | AD | CHARGE SYNDROME(214800) | Live birth | Retrospective |
| **252** | female | Cleft lip,Cleft palate | IRF6(607199) | NM_006147.3 | c.607C>T | p.(Pro203Ser) | missense | Het | VUS | De novo | AD | VAN DER WOUDE SYNDROME 1(119300) | Live birth | Retrospective |
| **253** | male | Cleft palate, Cleft lip | DHCR7(602858) | NM_001360.2 | c.1060G>A | p.(Ala354Thr) | missense | Het | VUS | Pat | AR | SMITH-LEMLI-OPITZ SYNDROME (270400) | Live birth | Retrospective |
|  |  |  |  | NM_001360.2 | c.289G>A | p.(Ala97Thr) | missense | Het | VUS | Mat |  |  |  |  |
| **254** | male | Cleft palate, Cleft lip | HSPG2(142461) | NM_001291860.1 | c.6467G>A | p.(Arg2156His) | missense | Het | VUS | Mat | AR | DYSSEGMENTAL DYSPLASIA, SILVERMAN-HANDMAKER TYPE(224410);SCHWARTZ-JAMPEL SYNDROME, TYPE 1(255800) | Live birth | Retrospective |
|  |  |  |  | NM_001291860.1 | c.1189C>T | p.(Arg397Trp) | missense | Het | VUS | Pat |  |  |  |  |
| **255** | male | Cleft palate, Cleft lip | NIPBL(608667) | NM_133433.3 | c.6954+8T>C | - | splice_region | Het | VUS | De novo | AD | CORNELIA DE LANGE SYNDROME 1(122470) | Termination | Retrospective |
| **256** | female | Cleft palate | LRP2(600073) | NM_004525.2 | c.10027C>T | p.(Arg3343Cys) | missense | Het | VUS | Mat | AR | DONNAI-BARROW SYNDROME(222448) | Live birth | Retrospective |
|  |  |  |  | NM_004525.2 | c.7306A>G | p.(Thr2436Ala) | missense | Het | VUS | Pat |  |  |  |  |
| **257** | male | Cleft palate | HUWE1(300697) | NM_031407.5 | c.12067C>T | p.(Arg4023Cys) | missense | Hemi | VUS-high risk | Mat | XL | MENTAL RETARDATION, X-LINKED, SYNDROMIC, TURNER TYPE(309590) | Live birth | Retrospective |
| **258** | male | Cleft palate,Cleft lip | FREM2(608945) | NM_207361.5 | c.5237T>C | p.(Met1746Thr) | missense | Het | VUS | Mat | AR | FRASER SYNDROME 1(219000) | Termination | Prospective |
|  |  |  |  | NM_207361.5 | c.6322G>A | p.(Ala2108Thr) | missense | Het | VUS | Pat |  |  |  |  |
| **259** | female | Micrognathia, Glossoptosis, Polyhydramnios | COL11A2(120290) | NM_080680.2 | c.4392+1G>A |  | splice_donor | Het | VUS-high risk | Pat | AD | OTOSPONDYLOMEGAEPIPHYSEAL DYSPLASIA, AUTOSOMAL RECESSIVE(215150) | Live birth | Prospective |
| **260** | male | Cataract | CENPF(600236) | NM_016343.3 | c.1373A>C | p.(Gln458Pro) | missense | Het | VUS | Mat | AR | STROMME SYNDROME(243605) | In Pregnancy | Prospective |
|  |  |  |  | NM_016343.3 | c.4382T>C | p.(Val1461Ala) | missense | Het | VUS | Pat |  |  |  |  |
| **261*** | female | Cleft lip and palate | FGF8(600483) | NM_033163.3 | c.418A>T | p.(Asn140Tyr) | missense | Het | VUS | Pat | AD | HYPOGONADOTROPIC HYPOGONADISM 6 WITH OR WITHOUT ANOSMIA(612702) | Termination | Prospective |
| **262** | female | Micrognathia, Fetal choroid plexus cysts | POLR1A(616404) | NM_015425.4 | c.1306C>G | p.(Arg436Gly) | missense | Het | VUS | De novo | AD | ACROFACIAL DYSOSTOSIS, CINCINNATI TYPE(616462) | Neonatal death | Prospective |
| **Chest** | |  |  |  |  |  |  |  |  |  |  |  |  |  |
| **263** | female | Pulmonary sequestration,Congenital cystic adenomatoid malformation of the lung | HSPG2(142461) | NM_001291860.1 | c.11717G>T | p.(Gly3906Val) | missense | Het | VUS | Pat | AR | DYSSEGMENTAL DYSPLASIA, SILVERMAN-HANDMAKER TYPE(224410) | Termination | Retrospective |
|  |  |  |  | NM_001291860.1 | c.1078C>T | p.(Pro360Ser) | missense | Het | VUS | Mat |  |  |  |  |
| **264** | male | Hypoplasia of the lungs | ZIC3(300265) | NM_003413.3 | c.1306C>G | p.(Leu436Val) | missense | Hemi | VUS-high risk | Mat | XR | X-LINKED HETEROTAXY, VISCERAL 1 (306955), X-LINKED VACTERL ASSOCIATION WITH OR WITHOUT HYDROCEPHALUS(314390) | Live birth | Retrospective |
| **265** | male | Pulmonary sequestration | STRA6(610745) | NM_001199042.1 | c.436T>C | p.(Phe146Leu) | missense | Het | VUS | Pat | AR | MICROPHTHALMIA, SYNDROMIC 9(601186) | Live birth | Prospective |
|  |  |  |  | NM_001199042.1 | c.367A>G | p.(Arg123Gly) | missense | Het | VUS | Mat |  |  |  |  |
| **266** | male | Increased nuchal translucency, Pleural effusion | RLIM(300379) | NM_016120 | c.1093C>T | p.(R365C) | missense | Hemi | VUS-high risk | De novo | XL | TONNE-KALSCHEUER SYNDROME(300978) | Termination | Prospective |
| **267*** | male | Cleft palate,Cleft lip | CDH1(192090) | NM_004360.4 | c.1625T>G | p.(Ile542Ser) | missense | Het | VUS-high risk | Mat(affected) | AD | BLEPHAROCHEILODONTIC SYNDROME 1(119580) | Termination | Prospective |
| **Cardiovascular** | | |  |  |  |  |  |  |  |  |  |  |  |  |
| **268** | male | Tetralogy of Fallot | ABL1(189980) | NM_007313.2 | c.431A>G | p.(His144Arg) | missense | Het | VUS | De novo | AD | CONGENITAL HEART DEFECTS AND SKELETAL MALFORMATIONS SYNDROME(617602) | Termination | Retrospective |
| **269** | female | Single ventricle, Single atrium, Persistent truncus arteriosus | GATA6(601656) | NM_005257.5 | c.1756C>T | p.(Pro586Ser) | missense | Het | VUS | De novo | AD | HEART DEFECTS, CONGENITAL, AND OTHER CONGENITAL ANOMALIES(600001) | Termination | Retrospective |
| **270** | female | Coarctation of aorta, Ventricular septal defect | SMARCA4(603254) | NM_001128849.1 | c.4795G>A | p.(Glu1599Lys) | missense | Het | VUS | De novo | AD | COFFIN-SIRIS SYNDROME 4(614609) | Live birth | Retrospective |
| **271** | female | Ventricular septal defect, Polyhydramnios | EFTUD2(603892) | NM_004247.3 | c.2407C>T | p.(Arg803Trp) | missense | Het | VUS | De novo | AD | MANDIBULOFACIAL DYSOSTOSIS, GUION-ALMEIDA TYPE(610536) | Live birth | Retrospective |
| **272** | female | Dextrocardia, Pulmonary artery atresia, Ventricular septal defect | MMP21(608416) | NM_147191.1 | c.557G>T | p.(Ser186Ile) | missense | Het | VUS-high risk | Mat | AR | HETEROTAXY, VISCERAL,7(608416) | Termination | Retrospective |
|  |  |  |  | NM_147191.1 | c.551C>T | p.(Ala184Val) | missense | Het | VUS | Pat |  |  |  |  |
| **273** | male | Tetralogy of fallot | FLNA(300017) | NM_001110556.1 | c.6002G>A | p.(Arg2001Gln) | missense | Hemi | VUS | Mat | XL | CARDIAC VALVULAR DYSPLASIA, X-LINKED(314400) | Live birth | Retrospective |
| **274** | male | Complete atrioventricular canal defect | MED12(300188) | NM_005120.2 | c.1963A>G | p.(Ser655Gly) | missense | Hemi | VUS | Mat | XR | LUJAN-FRYNS SYNDROME(309520) | Termination | Retrospective |
| **275** | female | Double outlet right ventricle, Ventricular septal defect, Coarctation of aorta | NOTCH1(190198) | NM_017617.4 | c.4781G>A | p.(Arg1594Gln) | missense | Het | VUS-high risk | De novo | AD | ADAMS-OLIVER SYNDROME 5(616028) | Live birth | Retrospective |
| **276** | male | Ventricular septal defect | PKD1L1(609721) | NM_138295.3 | c.6558+3A>G | - | splice_region | Het | VUS | Mat | AR | HETEROTAXY, VISCERAL, 8, AUTOSOMAL(617205) | Live birth | Retrospective |
|  |  |  |  | NM_138295.3 | c.3511G>A | p.(Val1171Met) | missense | Het | VUS | Pat |  |  |  |  |
| **277** | male | Ventricular septal defect | MIB1(608677) | NM_020774.3 | c.2119_2120dupCT | p.(Arg708Ter) | frameshift | Het | VUS | De novo | AD | LEFT VENTRICULAR NONCOMPACTION 7(615092) | Live birth | Retrospective |
| **278*** | male | Cardiac rhabdomyoma | TSC2(191092) | NM_000548.4 | c.778A>T | p.(Met260Leu) | missense | Het | VUS | Mat | AD | TUBEROUS SCLEROSIS 2(613254) | Live birth | Prospective |
|  |  |  | TSC2(191092) | NM_000548.4 | c.5017_5019dupGTC | p.(Val1673dup) | inframe | Het | VUS | Pat |  |  |  |  |
| **279** | female | Transposition of the great arteries | MED13L(608771) | NM_015335.4 | c.5456C>T | p.(Ala1819Val) | missense | Het | VUS | Mat | AD | TRANSPOSITION OF THE GREAT ARTERIES(608808) | Live birth | Prospective |
| **280** | male | Tetralogy of Fallot | JAG1(601920) | NM_000214.2 | c.776G>A | p.(Gly259Asp) | missense | Het | VUS | Mat | AD | ALAGILLE SYNDROME 1(118450) | Termination | Prospective |
| **281** | female | Congenitally corrected transposition of the great arteries with ventricular septal defect | GDF1(602880) | NM_001492.5 | c.480_481delinsA | p.(Gly161AlafsTer5) | frameshift | Het | VUS | Pat | AD | CONGENITAL HEART DEFECTS, MULTIPLE TYPES(613854) | Termination | Prospective |
| **282** | male | Complete atrioventricular canal defec, Right aortic arch, Left atrial isomerism, Heterotaxy | STAG2(300826) | NM_001042749.2 | c.3407A>T | p.(Asp1136Val) | missense | Hemi | VUS | Mat | XL | MULLEGAMA-KLEIN-MARTINEZ SYNDROME(301022) | Termination | Prospective |
| **283** | female | Cardiac rhabdomyoma | TSC2(191092) | NM_000548.4 | c.3989C>T | p.(Thr1330Met) | missense | Het | VUS | Pat | AD | TUBEROUS SCLEROSIS 2(613254) | Live birth | Prospective |
| **284** | female | Tetralogy of Fallot | IFIH1(606951) | NM_022168.3 | c.557G>A | p.(Arg186His) | missense | Het | VUS | De novo | AD | SINGLETON-MERTEN SYNDROME 1(182250) | Termination | Prospective |
| **285** | female | Ventricular septal defect, Cardiomegaly | MYH7(160760) | NM_000257.3 | c.545C>A | p.(Ala182Glu) | missense | Het | VUS | Pat | AD | CARDIOMYOPATHY, FAMILIAL HYPERTROPHIC, 1(192600) | Live birth | Prospective |
| **286** | male | Cardiac rhabdomyoma | TSC2(191092) | NM_000548.4 | c.677T>A | p.(Val226Asp) | missense | Het | VUS | Pat | AD | TUBEROUS SCLEROSIS 2(613254) | In Pregnancy | Prospective |
| **Isolated Hydrops** | | |  |  |  |  |  |  |  |  |  |  |  |  |
| **287** | male | Hydrops fetalis, Polyhydramnios | FLNB(603381) | NM_001164317.1 | c.2323+5G>C | - | splice_region | Het | VUS | De novo | AD | ATELOSTEOGENESIS, TYPE I(108720);ATELOSTEOGENESIS, TYPE (108721);LARSEN SYNDROME(150250) | Termination | Retrospective |
| **288** | female | Ascites, Pericardial effusion | LAMB2(150325) | NM_002292.3 | c.4304C>T | p.(Pro1435Leu) | missense | Het | VUS | Pat | AR | PIERSON SYNDROME(609049) | Live birth | Prospective |
|  |  |  |  | NM_002292.3 | c.3339G>T | p.(Gln1113His) | missense | Het | VUS | Mat |  |  |  |  |
| **289*** | male | Hydrops fetalis, Polyhydramnios | EPHB4(600011) | NM_004444.4 | c.2354G>A | p.(Arg785Gln) | missense | Het | VUS-high risk | Mat | AD | LYMPHATIC MALFORMATION 7(617300) | Live birth | Prospective |
| **290** | female | Pericardial effusion, Pleural effusion, Bradycardia | RAPSN(601592) | NM_005055.4 | c.690+4G>A |  | splice_region | Het | VUS | Pat | AR | FETAL AKINESIA DEFORMATION SEQUENCE 2(618388) | Termination | Prospective |
|  |  |  |  | NM_005055.4 | c.457G>T | p.(Ala153Ser) | missense | Het | VUS | Mat |  |  |  |  |
| **291** | male | Hydrops fetalis | UBR1(605981) | NM_174916.2 | c.4750G>A | p.(Val1584Met) | missense | Het | VUS | Mat | AR | JOHANSON-BLIZZARD SYNDROME(243800) | Neonatal death | Prospective |
|  |  |  |  | NM_174916.2 | c.604G>A | p.(Val202Ile) | missense | Het | VUS | Pat |  |  |  |  |
| **292** | female | Polyhydramnios, Hydrops fetalis | EPHB4(600011) | NM_004444.4 | c.2209G>A | p.(Val737Ile) | missense | Het | VUS | Pat | AD | LYMPHATIC MALFORMATION 7(617300) | Live birth | Prospective |
| **293** | male | Increased nuchal translucency, Hydrops fetalis | ADNP(611386) | NM_015339.4 | c.553T>A | p.(Tyr185Asn) | missense | Het | VUS | De novo | AD | HELSMOORTEL-VAN DER AA SYNDROME(615873) | Termination | Prospective |
| **Urogenital** | | |  |  |  |  |  |  |  |  |  |  |  |  |
| **294** | male | Multicystic kidney dysplasia | NOTCH2(600275) | NM_024408.3 | c.2453C>T | p.(Thr818Ile) | missense | Het | VUS | De novo | AD | HAJDU-CHENEY SYNDROME(102500);ALAGILLE SYNDROME 2(610205) | Live birth | Retrospective |
| **295** | male | Multicystic kidney dysplasia | NPHP3(608002) | NM_153240.4 | c.2261C>A | p.(Ser754Tyr) | missense | Het | VUS | Mat | AR | RENAL-HEPATIC-PANCREATIC DYSPLASIA 1(208540) | Live birth | Retrospective |
|  |  |  |  | NM_153240.4 | c.652A>C | p.(Asn218His) | missense | Het | VUS | Pat |  |  |  |  |
| **296** | female | Renal agenesis | GATA3(131320) | NM_001002295.1 | c.59A>G | p.(Asn20Ser) | missense | Het | VUS | De novo | AD | HYPOPARATHYROIDISM, SENSORINEURAL DEAFNESS, AND RENAL DISEASE(146255) | Live birth | Retrospective |
| **297** | male | Ectopic kidney | IFT172(607386) | NM_015662.2 | c.3925G>A | p.(Gly1309Ser) | missense | Het | VUS | Pat | AR | SHORT-RIB THORACIC DYSPLASIA 10 WITH OR WITHOUT POLYDACTYLY(615630) | Live birth | Retrospective |
|  |  |  |  | NM_015662.2 | c.2218C>T | p.(Arg740Cys) | missense | Het | VUS | Mat |  |  |  |  |
| **298** | male | Multicystic kidney dysplasia | PKHD1(606702) | NM_138694.3 | c.10058T>G | p.(Leu3353Arg) | missense | Het | VUS-high risk | Pat | AR | POLYCYSTIC KIDNEY DISEASE 4 WITH OR WITHOUT POLYCYSTIC LIVER DISEASE(263200) | Live birth | Retrospective |
|  |  |  |  | NM_138694.3 | c.4798G>A | p.(Gly1600Arg) | missense | Het | VUS-high risk | Mat |  |  |  |  |
| **299** | male | Hydronephrosis, Renal duplication, Micropenis | LAS1L(300964) | NM_031206.4 | c.23G>A | p.(Gly8Glu) | missense | Hemi | VUS | Mat | XR | WILSON-TURNER X-LINKED MENTAL RETARDATION SYNDROME(309585) | Live birth | Prospective |
| **300** | male | Multicystic kidney dysplasia | TBX18(604613) | NM_001080508.2 | c.1649C>T | p.(Ser550Phe) | missense | Het | VUS | Mat | AD | CONGENITAL ANOMALIES OF KIDNEY AND URINARY TRACT 2; CAKUT2(143400) | Live birth | Prospective |
| **301** | male | Hydronephrosis | PGK1(311800) | NM_000291.3 | c.989G>A | p.(Arg330Gln) | missense | Hemi | VUS | Mat | XR | PHOSPHOGLYCERATE KINASE 1 DEFICIENCY(300653) | Live birth | Prospective |
| **302** | female | Enlarged kidney, Hyperechogenic kidneys | PKD1(601313) | NM_001009944.2 | c.8302G>A | p.(Val2768Met) | missense | Het | VUS | Pat | AD | POLYCYSTIC KIDNEY DISEASE 1 WITH OR WITHOUT POLYCYSTIC LIVER DISEASE(173900) | In Pregnancy | Prospective |
| **303** | female | Multicystic kidney dysplasia | BICC1(614295) | NM_001080512.2 | c.954C>G | p.(Ile318Met) | missense | Het | VUS | Pat | AD | RENAL DYSPLASIA, CYSTIC, SUSCEPTIBILITY TO(6014331) | Termination | Prospective |
| **304** | male | Multicystic kidney dysplasia, Ureterocele | SALL1(602218) | NM_002968.2 | c.598C>T | p.(Leu200Phe) | missense | Het | VUS | De novo | AD | TOWNES-BROCKS SYNDROME 1(107480) | Live birth | Prospective |
| **305** | male | Enlarged kidney, Hyperechogenic kidneys,Oligohydramnios | PKHD1(606702) | NM_138694.3 | c.3629G>A | p.(Gly121Glu） | missense | Het | LP | Mat | AR | POLYCYSTIC KIDNEY DISEASE 4 WITH OR WITHOUT POLYCYSTIC LIVER DISEASE(263200) | Termination | Prospective |
|  |  |  |  | NM_138694.3 | c.2891T>A | p.(Val964Asp) | missense | Het | VUS-high risk | Pat |  |  |  |  |
| **Skeletal** | | |  |  |  |  |  |  |  |  |  |  |  |  |
| **306** | female | Aphalangy of the hands, Abnormality of the metacarpal bones | FBN1(134797) | NM_000138.4 | c.2727A>C | p.(Glu909Asp) | missense | Het | VUS | De novo | AD | ACROMICRIC DYSPLASIA(102370) | Termination | Retrospective |
|  |  |  | NIPBL(608667) | NM_133433.3 | c.3365G>A | p.(Arg1122Lys) | missense | Het | VUS | De novo | AD | CORNELIA DE LANGE SYNDROME 1(122470) |  |  |
| **307** | male | Talipes equinovarus | FLNA(300017) | NM_001110556.1 | c.4537C>T | p.(Pro1513Ser) | missense | Hemi | VUS | De novo | XL | OTOPALATODIGITAL SYNDROME, TYPE I(311300) | Termination | Retrospective |
| **308** | male | Short long bone, Hand polydactyly | ACAN(155760) | NM_013227.3 | c.703A>T | p.(Ile235Phe) | missense | Hom | VUS | Pat/Mat | AR | SPONDYLOEPIMETAPHYSEAL DYSPLASIA, AGGRECAN TYPE(612813) | Termination | Retrospective |
| **309** | female | Short long bone | NPR2(108961) | NM_003995.3 | c.3058C>T | p.(Arg1020Trp) | missense | Het | VUS | Mat(affected) | AD | SHORT STATURE WITH NONSPECIFIC SKELETAL ABNORMALITIES; SNSK(616255) | Live birth | Prospective |
| **310** | male | Calcaneovalgus deformity | LOXL3(607163) | NM_032603.4 | c.39dupG | p.(Leu14AlafsTer21) | frameshift | Homo | VUS | Mat+Pat | AR | Autosomal recessive Stickler syndrome(ORPHA:250984) | Live birth | Prospective |
| **311** | male | Talipes equinovarus | AP4B1(607245) | NM_006594.4 | c.1591C>T | p.(Arg531Trp) | missense | Het | VUS | Pat | AR | SPASTIC PARAPLEGIA 47(614066) | Live birth | Prospective |
|  |  |  |  | NM_006594.4 | c.1136C>T | p.(Thr379Ile) | missense | Het | VUS | Mat |  |  |  |  |
| **312** | female | Talipes equinovarus | CHRNG(100730) | NM_005199.4 | c.130G>A | p.(Ala44Thr) | missense | Het | VUS | Pat | AR | MULTIPLE PTERYGIUM SYNDROME, ESCOBAR VARIANT(265000) | Termination | Prospective |
|  |  |  |  | NM_005199.4 | c.1036-4_1036-3delCT | - | splice_region | Het | VUS | Mat |  |  |  |  |
| **313** | male | Abnormal posturing of hand | ATP7A(300011) | NM_000052.6 | c.4479G>C | p.(Arg1493Ser) | missense | Hemi | VUS | Mat | XR | OCCIPITAL HORN SYNDROME(304150);SPINAL MUSCULAR ATROPHY, DISTAL, X-LINKED 3(300489) | Live birth | Prospective |
| **314** | male | Scoliosis, Cervical C5/C6 vertebrae fusion, Lumbar hemivertebrae | RBM10(300080) | NM_001204468.1 | c.1980+7G>C | - | splice_region | Hemi | VUS | Mat | XR | TARP SYNDROME(311900) | Termination | Prospective |
| **315** | male | Butterfly vertebrae, Lumbar hemivertebrae | ERCC6(609413) | NM_000124.3 | c.3061A>G | p.(Ile1021Val) | missense | Het | VUS | Pat | AR | COCKAYNE SYNDROME B(133540) | Termination | Prospective |
|  |  |  |  | NM_000124.3 | c.1822-6T>A |  | splice_region | Het | VUS | Mat |  |  |  |  |
| **316** | male | Short long bone | TTC21B(612014) | NM_024753.4 | c.2449G>T | p.(Ala817Ser) | missense | Het | VUS | Pat | AR | SHORT-RIB THORACIC DYSPLASIA 4 WITH OR WITHOUT POLYDACTYLY(613819) | Live birth | Prospective |
|  |  |  |  | NM_024753.4 | c.235T>C | p.(Tyr79His) | missense | Het | VUS | Mat |  |  |  |  |
| **317** | male | Severe short-limb dwarfism | CENPJ(609279) | NM_018451.4 | c.3477+3delT |  | splice_region | Het | VUS | Pat | AR | SECKEL SYNDROME 4(613676) | Termination | Prospective |
|  |  |  |  | NM_018451.4 | c.1270A>G | p.(Thr424Ala) | missense | Het | VUS | Mat |  |  |  |  |
| **318** | male | Tetraphocomelia | LBR(600024) | NM_002296.4 | c.1757G>A | p.(Arg586His) | missense | Homo | VUS-high risk | Mat+Pat | AR | GREENBERG DYSPLASIA(215140) | Termination | Prospective |
| **319** | male | Short femur | HCFC1(300019) | NM_005334.2 | c.3238T>C | p.(Cys1080Arg) | missense | Hemi | VUS | Mat | XR | METHYLMALONIC ACIDEMIA AND HOMOCYSTEINEMIA, cblX TYPE(309541) | Live birth | Prospective |
| **320** | female | Hemivertebrae, Thoracic kyphosis | DCHS1(603057) | NM_003737.3 | c.3101_3107delATCACCT | p.(Tyr1034LeufsTer25) | frameshift | Het | LP | Mat | AR | VAN MALDERGEM SYNDROME 1(601390) | Live birth | Prospective |
|  |  |  |  | NM_003737.3 | c.700C>T | p.(Pro234Ser) | missense | Het | VUS | Pat |  |  |  |  |
| **321** | male | Short long bone, Abnormality of calvarial morphology, Short fetal femur length | TRPV6(606680) | NM_018646.5 | c.1168C>T | p.(Arg390Cys) | missense | Homo | VUS-high risk | Mat+Pat | AR | HYPERPARATHYROIDISM, TRANSIENT NEONATAL(618188) | Termination | Prospective |
| **Abdominal** | | |  |  |  |  |  |  |  |  |  |  |  |  |
| **322** | female | Duodenal atresia | PIEZO2(613629) | NM_022068.3 | c.3476G>A | p.(Arg1159Lys) | missense | Het | VUS | De novo | AD | MARDEN-WALKER SYNDROME(248700) | Live birth | Retrospective |
| **323** | male | Omphalocele | FREM1(608944) | NM_144966.5 | c.5596C>G | p.(His1866Asp) | missense | Het | VUS | Mat | AR | MANITOBA OCULOTRICHOANAL SYNDROME(248450) | Termination | Prospective |
|  |  |  |  | NM_144966.5 | c.4738C>T | p.(Arg1580Trp) | missense | Het | VUS | Pat |  |  |  |  |
| **324** | female | Omphalocele | LRP2(600073) | NM_004525.2 | c.11341T>G | p.(Tyr3781Asp) | missense | Het | VUS | Pat | AR | DONNAI-BARROW SYNDROME(222448) | Live birth | Prospective |
|  |  |  |  | NM_004525.2 | c.9070G>A | p.(Glu3024Lys) | missense | Het | VUS | De novo |  |  |  |  |
| **Increased NT and cystic hygroma** | | |  |  |  |  |  |  |  |  |  |  |  |  |
| **325** | male | Increased nuchal translucency | CC2D2A(612013) | NM_001080522.2 | c.1484G>A | p.(Arg495His) | missense | Het | VUS | Mat | AR | JOUBERT SYNDROME 9(612285) | Live birth | Retrospective |
|  |  |  |  | NM_001080522.2 | c.4186A>G | p.(Thr1396Ala) | missense | Het | VUS | Pat |  |  |  |  |
| **326** | male | Increased nuchal translucency | TPM2(190990) | NM_213674.1 | c.67G>A | p.(Glu23Lys) | missense | Het | VUS | De novo | AD | ARTHROGRYPOSIS, DISTAL, TYPE 1A(108120) | Live birth | Retrospective |
| **327** | male | Increased nuchal translucency | MECP2(300005) | NM_001110792.1 | c.1450G>C | p.(Glu484Gln) | missense | Hemi | VUS | Mat | XR | MENTAL RETARDATION, X-LINKED, SYNDROMIC 13(300055) | Live birth | Prospective |
| **FGR** | | |  |  |  |  |  |  |  |  |  |  |  |  |
| **328** | male | FGR, Oligohydramnios | FLNB(603381) | NM_001164317.1 | c.3661A>G | p.(Lys1221Glu) | missense | Het | VUS | Mat | AR | SPONDYLOCARPOTARSAL SYNOSTOSIS SYNDROME(272460) | Live birth | Retrospective |
|  |  |  |  | NM_001164317.1 | c.7762G>A | p.(Glu2588Lys) | missense | Het | VUS | Pat |  |  |  |  |
| **329** | female | FGR, Polyhydramnios | DOCK6(614194) | NM_020812.3 | c.5227G>A | p.(Gly1743Ser) | missense | Het | VUS | Pat | AR | ADAMS-OLIVER SYNDROME 2; AOS2(614219) | Live birth | Prospective |
|  |  |  |  | NM_020812.3 | c.4579C>T | p.(Arg1527Cys) | missense | Het | VUS | Mat |  |  |  |  |
| **330** | male | FGR, Polyhydramnios | NEB(161650) | NM_001164508.2 | c.6325A>G | p.(Thr2109Ala) | missense | Homo | VUS | Mat+Pat | AR | NEMALINE MYOPATHY 2(256030) | Live birth | Prospective |
| **331** | male | FGR, Polyhydramnios | CTU2(617057) | NM_001318507.1 | c.469G>A | p.(Glu157Lys) | missense | Het | VUS | Pat | AR | MICROCEPHALY, FACIAL DYSMORPHISM, RENAL AGENESIS, AND AMBIGUOUS GENITALIA SYNDROME(618142) | In Pregnancy | Prospective |
|  |  |  |  | NM_001318507.1 | c.1473C>T | p.(Pro491=) | synonymous | Het | VUS | Mat |  |  |  |  |
| **332** | male | Intrauterine growth retardation | SCN3A(182391) | NM_006922.3 | c.5197C>T | p.(Pro1733Ser) | missense | Het | VUS | De novo | AD | EPILEPTIC ENCEPHALOPATHY, EARLY INFANTILE, 62(617938) | Termination | Prospective |
| **Multisystem** | | |  |  |  |  |  |  |  |  |  |  |  |  |
| **333** | male | Dilation of lateral ventricles, Spina bifida, Abnormal joint morphology, Arnold-Chiari malformation | KCNQ5(607357) | NM_001160133.1 | c.1799C>T | p.(Thr600Ile) | missense | Het | VUS | De novo | AD | MENTAL RETARDATION, AUTOSOMAL DOMINANT 46(617601) | Termination | Retrospective |
| **334** | male | Renal hypoplasia, Ectopic kidney, Ascites, Pericardial effusion | JAG1(601920) | NM_000214.2 | c.138C>A | p.(Asn46Lys) | missense | Het | VUS | De novo | AD | ALAGILLE SYNDROME 1(118450) | Termination | Retrospective |
| **335** | male | Omphalocele, Lymphatic vessel neoplasm | CD96(606037) | NM_198196.2 | c.1733C>A | p.(Pro578His) | missense | Het | VUS | De novo | AD | C SYNDROME(211750) | Termination | Retrospective |
| **336** | female | Transposition of the great arteries, Ventricular septal defect, Pulmonic stenosis, Renal agenesis | SMARCE1(603111) | NM_003079.4 | c.665C>G | p.(Ala222Gly) | missense | Het | VUS | De novo | AD | COFFIN-SIRIS SYNDROME 5(616938) | Termination | Retrospective |
| **337** | female | Micrognathia, Cleft palate, Ventricular septal defect, Left aortic arch with retroesophageal right subclavian artery, Persistent left superior vena cava | NONO(300084) | NM_007363.4 | c.425G>A | p.(Arg142His) | missense | Het | VUS | De novo | XL | MENTAL RETARDATION, X-LINKED, SYNDROMIC 34(300967) | Live birth | Retrospective |
| **338** | male | Left atrial isomerism,Double outlet right ventricle,Ventricular septal defect | DYNC2H1(603297) | NM_001080463.1 | c.6044G>A | p.(Arg2015Gln) | missense | Het | VUS | Mat | AR | SHORT-RIB THORACIC DYSPLASIA 3 WITH OR WITHOUT POLYDACTYLY(613091) | Termination | Retrospective |
|  |  |  |  | NM_001080463.1 | c.6857C>T | p.(Pro2286Leu) | missense | Het | VUS | Pat |  |  |  |  |
| **339** | male | Ventriculomegaly,Hypoplasia of fetal nasal bone,Short middle phalanx of the 5th finger | TP63(603273) | NM_003722.4 | c.1318C>G | p.(Gln440Glu) | missense | Het | VUS | De novo | AD | ECTRODACTYLY, ECTODERMAL DYSPLASIA, AND CLEFT LIP/PALATE SYNDROME 3(604292)；RAPP-HODGKIN SYNDROME(129400) | Live birth | Retrospective |
| **340** | male | Edema, Dysostosis multiplex, Decreased fetal movement, Abnormal posturing, Appendicular hypotonia, Absent gallbladder, Umbilical cord cyst, Polyhydramnios | HERC2(605837) | NM_004667.5 | c.11645G>A | p.(Arg3882His) | missense | Het | VUS | Pat | AR | MENTAL RETARDATION, AUTOSOMAL RECESSIVE 3(615516) | Termination | Retrospective |
|  |  |  |  | NM_004667.5 | c.7398G>A | p.(Met2466Ile) | missense | Het | VUS | Mat |  |  |  |  |
| **341*** | female | Agenesis of corpus callosum, Arachnoid cyst, Polydactyly | IFT80 | NM_020800.2 | c.560A>C | p.(His187Pro) | missense | Het | VUS | Pat | AR | SHORT-RIB THORACIC DYSPLASIA 2 WITH OR WITHOUT POLYDACTYLY(611263) | Termination | Prospective |
|  |  |  |  | NM_020800.2 | c.1392G>A | p.(Leu464=) | synonymous | Het | VUS | Mat |  |  |  |  |
|  |  |  | NOTCH3(600276) | NM_000435.2 | c.4738T>A | p.(Ser1580Thr) | missense | Het | VUS | De novo | AD | LATERAL MENINGOCELE SYNDROME(130720) | Termination | Prospective |
| **342** | male | Bilateral cleft lip and palate,Micrognathia,Single ventricle,Pulmonic stenosis,Situs inversus totalis | FLNA(300017) | NM_001110556.1 | c.560A>G | p.(Asn187Ser) | missense | Hemi | VUS-high risk | Mat | XL | CARDIAC VALVULAR DYSPLASIA, X-LINKED(314400) | Termination | Prospective |
| **343*** | female | Talipes equinovarus, Micrognathia,Hydrops fetalis, Hyperechogenic kidneys, Abnormal hand morphology | HSPG2(142461) | NM_005529.6 | c.12238G>A | p.(Val4080Met) | missense | Het | VUS | Mat | AR | DYSSEGMENTAL DYSPLASIA, SILVERMAN-HANDMAKER TYPE(224410);SCHWARTZ-JAMPEL SYNDROME, TYPE 1(255800) | Lost to follow up | Prospective |
|  |  |  |  | NM_005529.6 | c.10803C>T | p.(Tyr3601=) | synonymous | Het | VUS | Pat |  |  |  |  |
| **344** | male | Absent radius, Hypospadias, Persistent left superior vena cava, Single umbilical artery | FLNB(603381) | NM_001164317.1 | c.6414C>T | p.(Ser2138=) | synonymous | Het | VUS | Pat | AR | SPONDYLOCARPOTARSAL SYNOSTOSIS SYNDROME (272460) | Termination | Prospective |
|  |  |  |  | NM_001164317.1 | c.6682C>T | p.(Arg2228Trp) | missense | Het | VUS | Mat |  |  |  |  |
| **345** | male | Dilation of lateral ventricles,Pleural effusion,Complete atrioventricular canal defect,Double outlet right ventricle,Coarctation in the transverse aortic arch,Hypoplastic left heart | PIEZO1(611184) | NM_001142864.3 | c.5214+7C>T | - | splice_region | Het | VUS | Mat | AR | LYMPHATIC MALFORMATION 6(616843) | Termination | Prospective |
|  |  |  |  | NM_001142864.3 | c.3590T>C | p.(Leu1197Pro) | missense | Het | VUS | Pat |  |  |  |  |
| **346*** | male | Cerebellar agenesis, Abnormality of the septum pellucidum, Congenital cataract, Aphalangy of the hands,Abnormality of the metacarpal bones, Intrauterine growth retardation | PHGDH(606879) | NM_006623.3 | c.511-5A>G | - | splice_region | Hom | VUS-high risk | Pat&Mat | AR | NEU-LAXOVA SYNDROME 1; NLS1(256520) | Termination | Prospective |
| **347*** | female | Meningoencephalocele, Multicystic kidney dysplasia, Ventricular septal defect | CC2D2A(612013) | NM_001080522.2 | c.1260T>G | p.(Cys420Trp) | missense | Het | VUS-high risk | Mat | AR | MECKEL SYNDROME, TYPE 6(612284) | Termination | Prospective |
|  |  |  |  | NM_001080522.2 | c.1266C>G | p.(Ser422Arg) | missense | Het | VUS-high risk | Mat |  |  |  |  |
|  |  |  |  | NM_001080522.2 | c.3641A>G | p.(Tyr1214Cys) | missense | Het | VUS-high risk | Pat |  |  |  |  |
| **348** | male | Cardiomegaly, Tricuspid regurgitation, Pericardial effusion, Hyperechogenic kidneys, Echogenic fetal bowel | SOS1(182530) | NM_005633.3 | c.979A>G | p.(Ile327Val) | missense | Het | VUS | De novo | AD | NOONAN SYNDROME 4(610733) | Termination | Prospective |
| **212*** | female | Pulmonary hypoplasia, Hyperechogenic kidneys, Micrognathia, Hydrops fetalis | TBCK(616899) | NM_001163436.2 | c.2501delC | p.(Thr834MetfsTer30) | frameshift | Het | LP | Pat | AR | HYPOTONIA, INFANTILE, WITH PSYCHOMOTOR RETARDATION AND CHARACTERISTIC FACIES 3(616900) | Termination | Prospective |
|  |  |  |  | NM_001163436.2 | c.2420G>A | p.(Arg807His) | missense | Het | VUS | Mat |  |  |  |  |
| **349** | male | Retrognathia, Coarctation of aorta, Dilatation of the descending thoracic aorta | CPLANE1(614571) | NM_023073.3 | c.4575_4577delAGA | p.(Glu1525del) | inframe | Het | VUS | Mat | AR | OROFACIODIGITAL SYNDROME VI(277170) | Live birth | Prospective |
|  |  |  |  | NM_023073.3 | c.3777_3788delCCACAAGCTTGA | p.(His1260_Asp1263del) | inframe | Het | VUS | Pat |  |  |  |  |
| **350**** | male | Omphalocele, Abnormality of the adrenal glands, Hyperechogenic kidneys | SOX4(184430) | NM_003107.2 | c.1022_1044dupGCCGCAGCAGCGCCGCCTCGTCC | p.(Pro349AlafsTer68) | frameshift | Het | VUS | De novo | AD | COFFIN-SIRIS SYNDROME 10(618506) | In Pregnancy | Prospective |
| **351*** | male | Abnormal posturing of hand,Micrognathia,Polyhydramnios,FGR | DYNC2I2 | NM_052844.3 | c.1551G>A | p.(Thr517=) | missense | Homo | VUS | Mat+Pat | AR | SHORT-RIB THORACIC DYSPLASIA 11 WITH OR WITHOUT POLYDACTYLY(615633) | Termination | Prospective |
| **352** | male | Oligohydramnios, Congenital posterior urethral valve, Hydronephrosis, Hydroureter | FREM2(608945) | NM_207361.5 | c.4396C>T | p.(Arg1466Ter) | missense | Het | LP | Pat | AR | FRASER SYNDROME 2(617666) | Termination | Prospective |
|  |  |  |  | NM_207361.5 | c.8347A>T | p.(Ile2783Leu) | missense | Het | VUS-high risk | Mat |  |  |  |  |
| **217*** | female | Ventriculomegaly, Right renal agenesis, Left renal duplication, Ventricular septal defect, Persistent left superior vena cava,Small for gestational age | TBCK(616899) | NM_001163436.2 | c.1589G>A | p.(Arg530His) | missense | Homo | VUS | Mat+Pat | AR | HYPOTONIA, INFANTILE, WITH PSYCHOMOTOR RETARDATION AND CHARACTERISTIC FACIES 3(616900) | Termination | Prospective |
| **353** | female | Ventricular septal defect, Hyperechogenic kidneys | KIF14(611279) | NM_014875.2 | c.1672A>G | p.(Thr558Ala) | missense | Het | VUS | Pat | AR | MECKEL SYNDROME 12; MKS12(616258), MICROCEPHALY 20, PRIMARY, AUTOSOMAL RECESSIVE; MCPH20(617914) | Lost to follow up | Prospective |
|  |  |  |  | NM_014875.2 | c.1408T>A | p.(Phe470Ile) | missense | Het | VUS | Mat |  |  |  |  |
| **354** | female | Fetal cystic hygroma, Hydrops fetalis,Visceral isomerism syndrome,Double outlet right ventricle, Complete atrioventricular canal defect,Hyperechogenic kidneys | CACNA1D(114206) | NM_000720.3 | c.3271G>A | p.(Val1091Met) | missense | Het | VUS | De novo | AD | PRIMARY ALDOSTERONISM, SEIZURES, AND NEUROLOGIC ABNORMALITIES(615474) | Termination | Prospective |
| **355** | male | Ascites, Hepatomegaly, Splenomegaly, Oligohydramnios,Increased placental thickness | NPC1(607623) | NM_000271.4 | c.2526T>A | p.(Phe842Leu) | missense | Het | VUS-high risk | Mat | AR | NIEMANN-PICK DISEASE, TYPE C1(257220) | Termination | Prospective |
|  |  |  |  | NM_000271.4 | c.1226T>C | p.(Ile409Thr) | missense | Het | LP | De novo |  |  |  |  |
| **356*** | female | Ascites, Cardiomegaly, Tricuspid regurgitation, Agenesis of corpus callosum, Cerebellar hypoplasia, Polyhydramnios, Hydrops fetalis | TMEM260(617449) | NM_017799.3 | c.107T>C | p.(Val36Ala) | missense | Het | VUS | Mat | AR | STRUCTURAL HEART DEFECTS AND RENAL ANOMALIES SYNDROME (617478) | Termination | Prospective |
|  |  |  |  | NM_017799.3 | c.284A>G | p.(Asn95Ser) | missense | Het | VUS | Pat |  |  |  |  |
| **357*** | female | Situs inverse totalis, Abnormal vena cana morphology, Ventriculomegaly, Dextrocardia,Gastrointestinal obstruction | DNAH11(603339) | NM_001277115.2 | c.11392G>T | p.(Glu3798Ter) | nonsense | Het | P | Pat | AR | CILIARY DYSKINESIA PRIMARY, 7, WITH OR WITHOUT SITUS INVERSUS(611884) | Termination | Prospective |
|  |  |  |  | NM_001277115.2 | c.11374-18A>G | - | splice region | Het | VUS-high risk | Mat |  |  |  |  |
| **358** | male | Increased nuchal translucency, Cystic hygroma, Hypertelorism, Pleural effusion, Ascites | LZTR1(600574) | NM_006767.3 | c.741C>A | p.(Ser247Arg) | missense | Het | VUS | Mat | AR | NOONAN SYNDROME 2(605275) | Termination | Prospective |
|  |  |  |  | NM_006767.3 | c.1349G>A | p.(Gly450Asp) | missense | Het | VUS | Pat |  |  |  |  |
|  |  |  |  |  |  |  |  |  |  |  |  |  |  |  |
|  |  |  |  |  |  |  |  |  |  |  |  |  |  |  |

* 15 cases with significant family history.

** Case 350 diagnosed as Beckwith-Wiedemann syndrome by MS-MLPA.

**Additional file Table S9. Fetuses with IFs and SFs results detected by ES**

| **Case ID** | **Gender** | **Ultrasound finding** | **Gene(OMIM ID)** | **Transcript** | **Nucleotide change** | **Amino acid change** | **Variant type** | **Zygosity** | **Classification** | **Origin** | **Inheritance** | **Disease(OMIM ID)** | **Pregnancy outcome** | **Group** |
| --- | --- | --- | --- | --- | --- | --- | --- | --- | --- | --- | --- | --- | --- | --- |
| **ACMG secondary findings** | | |  |  |  |  |  |  |  |  |  |  |  |  |
| **46** | male | Tetralogy of Fallot | MSH6(600678) | NM_000179.2 | c.651_652insCT | p.(Lys218LeufsTer29) | frameshift | het | LP | Pat | AD | MISMATCH REPAIR CANCER SYNDROME 3(619097) | Termination | Retrospective |
| **177** | female | Esophageal atresia, Right aortic arch | COL3A1(120180) | NM_000090.3 | c.3271C>T | p.(Arg1091Cys) | missense | het | LP | De novo | AD | EHLERS-DANLOS SYNDROME, VASCULAR TYPE(130050) | Termination | Retrospective |
| **359** | female | Pulmonary artery atresia, Tetralogy of Fallot | DSG2(125671) | NM_001943.4 | c.952G>T | p.(Gly318Ter) | nonsense | het | LP | Mat | AD | ARRHYTHMOGENIC RIGHT VENTRICULAR DYSPLASIA, FAMILIAL, 10(610193) | Live birth | Retrospective |
| **360** | female | Double outlet right ventricle, Ventricular septal defect, Mitral stenosis, Abnormal morphology of the left ventricle | TGFBR1(190181)* | NM_001306210.1 | c.775C>T | p.(Arg259Cys) | missense | het | LP | De novo | AD | LOEYS-DIETZ SYNDROME 1(609192) | Termination | Retrospective |
| **361** | male | Multicystic kidney dysplasia | BRCA2(600185) | NM_000059.3 | c.4363G>T | p.(Glu1455Ter) | nonsense | het | LP | Mat | AD | BREAST-OVARIAN CANCER, FAMILIAL, SUSCEPTIBILITY TO, 2(612555) | Live birth | Retrospective |
| **362** | male | Increased nuchal translucency | SCN5A(600163)* | NM_198056.2 | c.4339T>C | p.(Tyr1447His) | missense | het | LP | De novo | AD | LONG QT SYNDROME 3(603830) | Live birth | Retrospective |
| **363** | male | Increased nuchal translucency | DSG2(125671) | NM_001943.4 | c.1879+1G>C | - | splice_donor | het | LP | Pat | AD | ARRHYTHMOGENIC RIGHT VENTRICULAR DYSPLASIA, FAMILIAL, 10(610193) | Live birth | Retrospective |
| **70** | male | Mitral regurgitation, Tricuspid regurgitation, Hypoplastic tricuspid valve, Cardiomegaly | TTN(188840) | NM_001267550.2 | c.96670delG | p.(Glu32224LysfsTer27) | frameshift | het | LP | De novo | AD | CARDIOMYOPATHY, DILATED, 1G(604145) | Termination | Prospective |
| **171** | male | Increased nuchal translucency, cystic hygroma | MSH2(609309) | NM_000251.3 | c.1457_1460delATGA | p.(Asn486ThrfsTer10) | frameshift | het | P | Pat(affected) | AD | MUIR-TORRE SYNDROME(158320) | Termination | Prospective |
| **364** | male | Single ventricle, Complete atrioventricular canal defect, Abnormality of the left ventricular outflow tract, Interrupted aortic arch | LDLR(606945) | NM_000527.4 | c.2108_2114dupTGCTGGC | p.(Arg706AlafsTer13) | frameshift | het | LP | Mat | AD | HYPERCHOLESTEROLEMIA, FAMILIAL, 1(143890) | Termination | Prospective |
| **365** | male | Cystic hygroma | COL3A1(120180) | NM_000090.3 | c.80-2A>T | - | splice_acceptor | het | LP | Mat | AD | EHLERS-DANLOS SYNDROME, VASCULAR TYPE(130050) | Neonatal death | Prospective |
| **366** | male | Increased nuchal translucency | SDHB(185470) | NM_003000.2 | c.183T>A | p.(Tyr61Ter) | nonsense | het | LP | Mat | AD | PHEOCHROMOCYTOMA(171300);PARAGANGLIOMAS 4(115310) | Live birth | Prospective |
| **367** | female | Right aortic arch with retroesophageal left subclavian artery | ATP7B(606882)* | NM_000053.4 | c.3443T>C | p.(Ile1148Thr) | missense | het | LP | Pat | AR | WILSON DISEASE(277900) | Live birth | Prospective |
|  |  |  |  | NM_000053.4 | c.2294A>G | p.(Asp765Gly) | missense | het | LP | Mat |  |  |  |  |
| **Incidental findings in childhood-onset disease genes** | | | | | | |  |  |  |  |  |  |  |  |
| **368** | male | Talipes equinovarus | EBF3(607407) | NM_001005463.2 | c.1013-1G>A | - | splice_acceptor | het | LP | De novo | AD | HYPOTONIA, ATAXIA, AND DELAYED DEVELOPMENT SYNDROME(617330) | Termination | Retrospective |
| **369** | male | double superior vena cava, Abnormal posturing | FOXP1(605515) | NM_001244810 | c.1146+1G>A | - | splice_donor | het | LP | De novo | AD | MENTAL RETARDATION WITH LANGUAGE IMPAIRMENT AND WITH OR WITHOUT AUTISTIC FEATURES(613670) | Live birth | Retrospective |
| **370** | female | Ventricular septal defect | BSCL2(606158) | NM_001122955.4 | c.974dupG | p.(Ile326HisfsTer12) | frameshift | het | LP | Mat | AR | LIPODYSTROPHY, CONGENITAL GENERALIZED, TYPE 2(269700) | Live birth | Retrospective |
|  |  |  |  | NM_001122955.4 | c.320G>A | p.(Trp107Ter) | nonsense | het | LP | Pat |  |  |  |  |
| **45** | female | Cleft palate | POGZ(614787) | NM_015100.3 | c.3812_3813delCT | p.(Thr1271SerfsTer7) | frameshift | het | LP | De novo | AD | WHITE-SUTTON SYNDROME(616364) | Termination | Prospective |
| **91** | female | Enlarged kidney, Hyperechogenic kidneys | GLUD1(138130) | NM_005271.4 | c.758C>T | p.(Ala253Val) | missense | het | LP | De novo | AD | HYPERINSULINEMIC HYPOGLYCEMIA, FAMILIAL, 6(606762) | Live birth | Prospective |
| **371** | male | Dilation of lateral ventricles, Talipes equinovaru, Abnormality of finger | ADCY5(600293) | NM_183357.2 | c.3193C>T | p.(Gln1065Ter) | nonsense | het | P | De novo | AD | DYSKINESIA, FAMILIAL, WITH FACIAL MYOKYMIA(606703) | Termination | Prospective |
| **372** | male | Increased nuchal translucency | INVS(243305) | NM_014425.5 | c.907-1G>A | - | splice_acceptor | Het | LP | Pat | AR | NEPHRONOPHTHISIS 2(602088) | Live birth | Prospective |
|  |  |  |  | NM_014425.5 | c.2887C>T | p.(Gln963Ter) | nonsense | Het | LP | Mat |  |  |  |  |
| **373** | male | Bilateral talipes equinovarus | ANKRD17(615929) | NM_032217.5 | c.1525C>T | p.(Arg509Ter) | nonsense | Het | LP | De novo | AD | CHOPRA-AMIEL-GORDON SYNDROME(619504) | In pregnancy | Prospective |
|  |  |  |  |  |  |  |  |  |  |  |  |  |  |  |

***** Secondary findings with childhood-onset disease included in the report.

**Additional file 2 Table S10. Candidate genes identified in this study**

| **Case Malformation** | **Candidate gene** | **Chromosome Location** | **Transcript** | **Nucleotide change** | **Amino acid change** | **Origin** | **Variant type** |
| --- | --- | --- | --- | --- | --- | --- | --- |
| Cardiovascular | *ZMYND8* | 20:45855988 | NM_001281775.2 | c.2974T>C | p.(Trp992Arg) | De novo | missense |
| Skeletal | *PLCH2* | 1:2428959 | NM_014638.3 | c.2135C>T | p.(Ser712Leu) | De novo | missense |
| Skeletal | *SEMA4B* | 15:90767102 | NM_001324031.1 | c.1235G>A | p.(Cys412Tyr) | De novo | missense |
| Cardiovascular | *RHBDF1* | 16:114940 | NM_022450.3 | c.74C>T | p.(Pro25Leu) | De novo | missense |
| Cardiovascular | *PIK3IP1* | 22:31685353 | NM_052880.4 | c.535G>T | p.(Val179Leu) | De novo | missense |
| Skeletal | *ENTPD8* | 9:140330583 | NM_001033113.1 | c.932G>A | p.(Gly311Asp) | De novo | missense |
| Cardiovascular | *DDX19A* | 16:70398448 | NM_018332.4 | c.389C>T | p.(Pro130Leu) | De novo | missense |
| Cardiovascular | *TMEM132D* | 12:129558833 | NM_133448.2 | c.2887G>T | p.(Asp963Tyr) | De novo | missense |
| Cardiovascular | *CEACAM19* | 19:45185867 | NM_020219.3 | c.823delG | p.(Glu275SerfsTer12) | De novo | frameshift |
| Cardiovascular | *TMEM88* | 17:7758480 | NM_203411.1 | c.88C>T | p.(Leu30Phe) | De novo | missense |
| Cardiovascular | *ARHGEF25* | 12:58008348 | NM_001111270.2 | c.893C>T | p.(Ser298Leu) | De novo | missense |
| Cardiovascular | *MMP11* | 22:24122840 | NM_005940.4 | c.554C>T | p.(Thr185Ile) | De novo | missense |
| Chest | *ILK* | 11:6631487 | NM_004517.3 | c.1187C>T | p.(Ser396Phe) | De novo | missense |
| Urogenital | *GBGT1* | 9:136029263 | NM_021996.5 | c.745G>A | p.(Ala249Thr) | De novo | missense |
| Skeletal | *JARID2* | 6:15496732 | NM_004973.3 | c.1277dupA | p.(Leu427AlafsTer97) | De novo | frameshift |
| Urogenital | *FBLN2* | 3:13679113 | NM_001004019.1 | c.3391delG | p.(Ala1131ArgfsTer36) | De novo | frameshift |
| Multisystem | *MYO5C* | 15:52488520 | NM_018728.3 | c.4981C>G | p.(Leu1661Val) | De novo | missense |
| Cardiovascular | *STARD9* | 15:42957487 | NM_020759.2 | c.1213G>A | p.(Glu405Lys) | De novo | missense |
| Cardiovascular | *DUSP14* | 17:35872702 | NM_007026.3 | c.328C>T | p.(His110Tyr) | De novo | missense |
| Cardiovascular | *DOCK9* | 13:99500876 | NM_015296.2 | c.4034T>G | p.(Phe1345Cys) | De novo | missense |
| Fetal hydrops | *E4F1* | 16:2282521 | NM_004424.4 | c.674G>T | p.(Cys225Phe) | De novo | missense |
| Central nervous | *WTAP* | 6:160174523 | NM_004906.4 | c.484C>T | p.(Arg162Ter) | De novo | nonsense |
| Multisystem | *ZBTB37* | 1:173839698 | NM_001122770.2 | c.335A>G | p.(Gln112Arg) | De novo | missense |
| Cardiovascular | *RC3H1* | 1:173962039 | NM_001300850.1 | c.84delC | p.(Ile29SerfsTer14) | De novo | frameshift |
| Urogenital | *PEAR1* | 1:156882670 | NM_001080471.1 | c.2318C>G | p.(Ala773Gly) | De novo | missense |
| Cardiovascular | *USP4* | 3:49330027 | NM_003363.3 | c.1888T>G | p.(Tyr630Asp) | De novo | missense |
| Urogenital | *ZBED4* | 22:50279534 | NM_014838.2 | c.2224G>A | p.(Glu742Lys) | De novo | missense |
| Cardiovascular | *FAM167A* | 8:11281887 | NM_053279.2 | c.640T>A | p.(Cys214Ser) | De novo | missense |
| Skeletal | *SETD1B* | 12:122247684 | NM_015048.1 | c.833G>A | p.(Arg278His) | De novo | missense |
| Urogenital | *ASTN1* | 1:177030347 | NM_004319.2 | c.338T>C | p.(Leu113Pro) | De novo | missense |
| Chest | *YWHAZ* | 8:101960871 | NM_003406.3 | c.247A>G | p.(Arg83Gly) | De novo | missense |
| Central nervous | *FRAT1* | 10:99079843 | NM_005479.3 | c.633C>A | p.(Asn211Lys) | De novo | missense |
| Cardiovascular | *SMAD2* | 18:45371799 | NM_005901.5 | c.1192G>T | p.(Val398Phe) | De novo | missense |
| Cardiovascular | *ASXL3** | 18:31319536 | NM_030632.2 | c.2168C>G | p.(Pro723Arg) | Pat | missense |
|  |  | 18:31325261 | NM_030632.2 | c.5449C>G | p.(Pro1817Ala) | Mat | missense |

* Different mode of inheritance proposed than the known gene-disease relationship.

**Additional file 2 Table S11. Pregnancy outcomes of the study cohort**

| **Pregnancy outcome** | **Diagnostic cases** | **Inconclusive cases** | **Secondary findings** | **Incidental findings** | **Negative cases** | **Total cases** |
| --- | --- | --- | --- | --- | --- | --- |
| **Termination** | 163 | 61 | 2 | 2 | 351 | **579** |
| Cases with additional phenotypes | 17 | 7 | 0 | 0 | 40 | 64 |
| **Fetal demise** | 0 | 0 | 0 | 0 | 5 | **5** |
| **Lost to follow-up** | 1 | 2 | 0 | 0 | 92 | **95** |
| **In pregnancy** | 2 | 5 | 0 | 1 | 53 | **61** |
| **Live birth** | 63 | 61 | 7 | 3 | 744 | **878** |
| Neonatal demise | 5 | 2 | 1 | 0 | 11 | 19 |
| Cases with additional phenotypes | 32 | 22 | 2 | 2 | 171 | 229 |
| **Total** | **229** | **129** | **9** | **6** | **1245** | **1618** |
